# Quantum‐Trained AI Enables Inverse Design of Organic Frontier Orbitals at Billion‐Scale

**DOI:** 10.1002/advs.77414

**Published:** 2026-08-24

**Authors:** Yeongnam Ko, Se Jin Kim, Ki Chul Kim

**Affiliations:** ^1^ Computational Materials Design Laboratory School of Chemical Biomolecular and Energy Engineering Konkuk University Seoul The Republic of Korea

**Keywords:** artificial intelligence, density functional theory, frontier orbital, inverse design, organic molecule

## Abstract

Frontier orbital energies govern charge transfer, energy‐level alignment, and optoelectronic performance in organic molecules, yet their prediction across vast chemical spaces remains challenging because they arise from coupled effects of functionality, conjugation, and topology. In this study, we develop an interpretable AI framework for frontier orbital prediction using QM9 quantum‐chemical dataset and validate it across conventional and deep learning models. A direct message passing neural network delivers the best performance for frontier orbital prediction, while interpretability analyses consistently recover chemically meaningful substructures linked to orbital modulation. Extension of the optimized DMPNN model to 908 545 821 molecules in GDB–13 database enables AI‐guided ultra large‐scale donor screening framework development, referencing frontier orbital alignment to a representative ITIC acceptor for solar cells. This identifies only 37 visible region candidates, revealing the rarity of simultaneously satisfying donor‐like level alignment and narrow optical gaps. Scaffold‐resolved analysis further unravels diamino‐triketone class as a privileged donor building‐unit motif, combining small positive frontier orbital offsets with narrow gaps and favorable energy alignment. These show that quantum‐trained AI can unify predictive accuracy, chemical interpretability, and billion‐scale screening, providing a practical route for inverse design of organic optoelectronic molecules.

## Graphical abstract

Quantum‐trained AI links molecular substructures to frontier‐orbital energetics and enables interpretable screening across nearly one billion GDB‐13 molecules. By combining fragment‐ and ring‐level insights with donor–acceptor energy alignment against ITIC, the framework narrows an immense chemical space to a small set of promising candidates and reveals diamino‐triketone scaffolds as privileged donor building units.

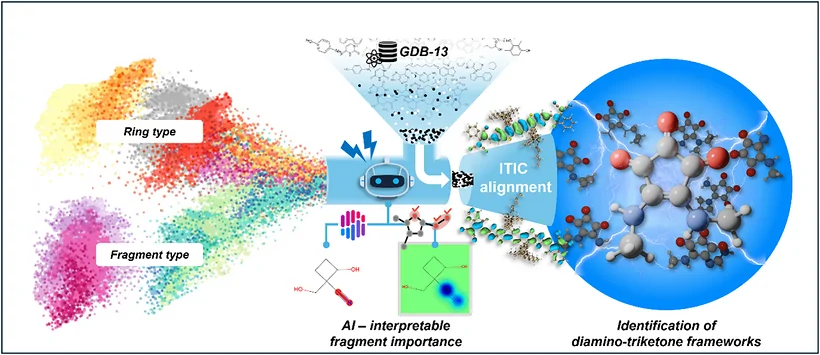

## 1 Introduction

The electronic energy levels of organic semiconductors, particularly the highest occupied molecular orbital (HOMO), the lowest unoccupied molecular orbital (LUMO), and the HOMO–LUMO gap, govern key processes in optoelectronic and energy‐conversion devices, including charge generation, charge transport, and interfacial energy‐level alignment [1, 2, 3, 4, 5, 6, 7]. In organic photovoltaics (OPVs), for example, the donor HOMO and acceptor LUMO largely determine the achievable open‐circuit voltage and the driving force for charge transfer and relevant current density, while the band gap (often approximated by the HOMO–LUMO gap at the molecular level) influences absorption and photophysical losses [8, 9, 10, 11]. Yet, rationally tuning these electronic properties remains difficult because they emerge from a coupled interplay among functional groups, conjugation length, heteroatom chemistry, and molecular topology [12, 13, 14, 15]. For instance, Iwasaki et al. showed that replacing alkyl substituents in a thienobenzobisthiazole‐based donor polymer with ester or acyl groups deepened the HOMO level and improved OPV performance, highlighting the sensitivity of frontier orbitals to local functionality [16]. Gao et al. demonstrated that varying terminal functional groups in carbazole‐based self‐assembled monolayers modulated energy levels, dipole moments, and molecular packing, thereby altering interfacial charge extraction and OPV performance [17]. Liu et al. reported that asymmetric end‐group design, side‐chain engineering, and halogenation in Y6‐based acceptors modulated electronic energy‐level distribution, molecular stacking, crystallinity, compatibility, ultimately affecting OPV performance. These studies emphasize that frontier orbital energetics can evolve in a highly coupled manner with molecular functionality, conjugation, and topology [18].

A central bottleneck in exploring organic molecules across interactive networks of such multiple factors is the size of chemical space [19, 20]. Even when restricted to small, synthetically plausible organic molecules, the number of candidates quickly becomes astronomical, making exhaustive quantum‐chemical evaluation impractical. While high‐fidelity electronic‐structure calculations (e.g., density functional theory (DFT) method) provide valuable targets for data‐driven modeling, the computational cost prevents their direct deployment as a screening engine at electronic scale [21]. For instance, Cheng et al. showed that the substitution space of aza‐substituted pentacenes was too vast for exhaustive screening, motivating the use of a confined chemical space and an evolutionary search to reduce computational cost [22]. This challenge creates a clear opportunity for artificial intelligence (AI) models that can learn structure–property relationships from reliable datasets and then generalize to much larger molecular universes, enabling fast exploration and targeted molecular discovery [23]. At the same time, AI‐driven molecular screening is often constrained by the limited size of experimentally available labeled datasets, highlighting the essential importance of large‐scale quantum‐chemical datasets. For example, Wu et al. constructed AI models from only 565 literature‐derived experimental donor/acceptor pairs for non‐fullerene organic solar cells and applied them to screen candidate combinations [24]. Among available quantum datasets, QM9 has become a widely used benchmark because it offers a large collection of equilibrium organic molecules with consistently computed properties, including HOMO, LUMO, and the HOMO–LUMO gap. Importantly, the QM9‐based molecular set spans broad electronic and structural diversity: the distributions of HOMO energies and gaps cover wide ranges, and the dataset contains a rich mixture of functional groups and ring topologies [25, 26]. Such diversity is essential for training robust predictors and for probing how chemical motifs relate to frontier orbital energetics [27]. However, simple “counting” descriptors (e.g., fragment frequencies) typically explain only a limited fraction of the variance in HOMO/LUMO/gap, indicating that frontier orbital energetics depend on higher‐order combinations of motifs, their connectivity, and local electronic environments rather than isolated functional groups [23].

Recent progress in molecular machine learning suggests that representation choice is decisive [28, 29]. Conventional pipelines (fingerprints or fragment descriptors + boosting/regression) can achieve reasonable accuracy and offer interpretability tools, such as Shapley additive explanations (SHAP), but may struggle to fully encode conjugation patterns and context‐dependent electronic effects [30, 31]. In contrast, deep learning models that operate on graphs or tokenized molecular sequences can learn richer structure‐aware representations, often yielding near‐DFT fidelity for frontier orbital prediction [32, 33, 34]. Furthermore, a successful inverse design not only requires prediction accuracy but also must provide *chemical insight* that can guide the construction of promising candidates and justify screening rules [35, 36]. A practical strategy, therefore, is to combine high‐performing AI predictors with interpretable analyses across multiple representations, so that recurring “important substructures” can be triangulated from both conventional machine learning explanations and deep‐learning attribution methods.

In this study, we develop an AI‐driven framework to predict HOMO, LUMO, and the HOMO–LUMO gap from molecular structure using QM9 quantum dataset as the training and analysis foundation. We leverage the learned structure–property relationships to propose an inverse design strategy aimed at identifying candidates that satisfy target energy‐level windows relevant to optoelectronic materials. The workflow proceeds from (i) a statistical and chemical‐diversity analysis of QM9 to (ii) systematic construction of graph/sequence‐based deep‐learning model platforms as well as non‐linear tree‐based gradient‐boosting models across fragment‐based descriptors and fingerprint‐based descriptors, and to (iii) interpretable structure–property correlation analyses that identify chemically meaningful motifs influencing frontier orbital energetics. Finally, we demonstrate how the best‐performing AI model can be scaled to ultra‐large chemical spaces by screening the QM9‐based molecular universe (hundreds of millions to near‐billion scale) under target‐driven criteria, enabling rapid identification and prioritization of promising molecular families for downstream materials design.

## 2 Results and Discussion

### 2.1 Dataset Analysis: Extension Capability of Computationally Available QM9 Dataset

QM9 dataset is a curated subset of the much larger virtual chemical universe, GDB‐17, which systematically enumerates 166.4 billion synthetically plausible organic molecules [25]. Notably, GDB‐17 comprises structures containing up to 17 non‐hydrogen atoms drawn from C, N, O, S, and halogens, generated under rules that enforce chemical stability and basic synthetic feasibility. Owing to this breath, GDB‐17 has been widely used in cheminformatics, molecular property prediction, and drug discovery as a benchmarking chemical space [27, 32]. However, because only a tiny fraction of its enumerated molecules is associated with experimentally measured or computationally evaluated properties, it remains challenging to establish application‐specific design strategies from large‐scale structure‐property analyses to pinpoint optimal candidates without expensive downstream calculations.

In contrast, its subset, QM9, provides a systematically organized library of small organic molecules with consistently computed DFT properties, offering a practical foundation for learning structure‐property relationships relevant to molecular electronics [26]. We particularly focus on frontier orbital descriptors directly available in QM9, HOMO and HOMO–LUMO gap, that are central to organic solar cell design. Notably, these quantities span broad ranges from −11.66 to −2.77 (median value: −6.56) eV for HOMO, from −4.76 to 5.27 (median value: 0.33) eV for LUMO, and from 0.67 to 16.93 (median value: 6.79) eV for HOMO–LUMO gap, indicating that QM9 samples diverse electronic regimes (Figure 1a–c). Moreover, molecules randomly drawn from nine high/medium/low combinations of HOMO and LUMO levels exhibit distinguishable structural motifs, suggesting that specific substructures (functionalities) and connectivity patterns may systematically modulate frontier orbital energies (Figure 1d,e). For instance, simple chain‐like molecules, such as methane and HCN, tend to exhibit relatively deep HOMO levels, whereas ring‐containing molecules are more frequently associated with higher HOMO levels. Likewise, introducing carbonyl moiety into pyrrole to form maleimide shifts both HOMO and LUMO levels to lower energies, whereas incorporating an amine functionality into furan shifts them in the opposite direction (Figure 1f).

**FIGURE 1:**
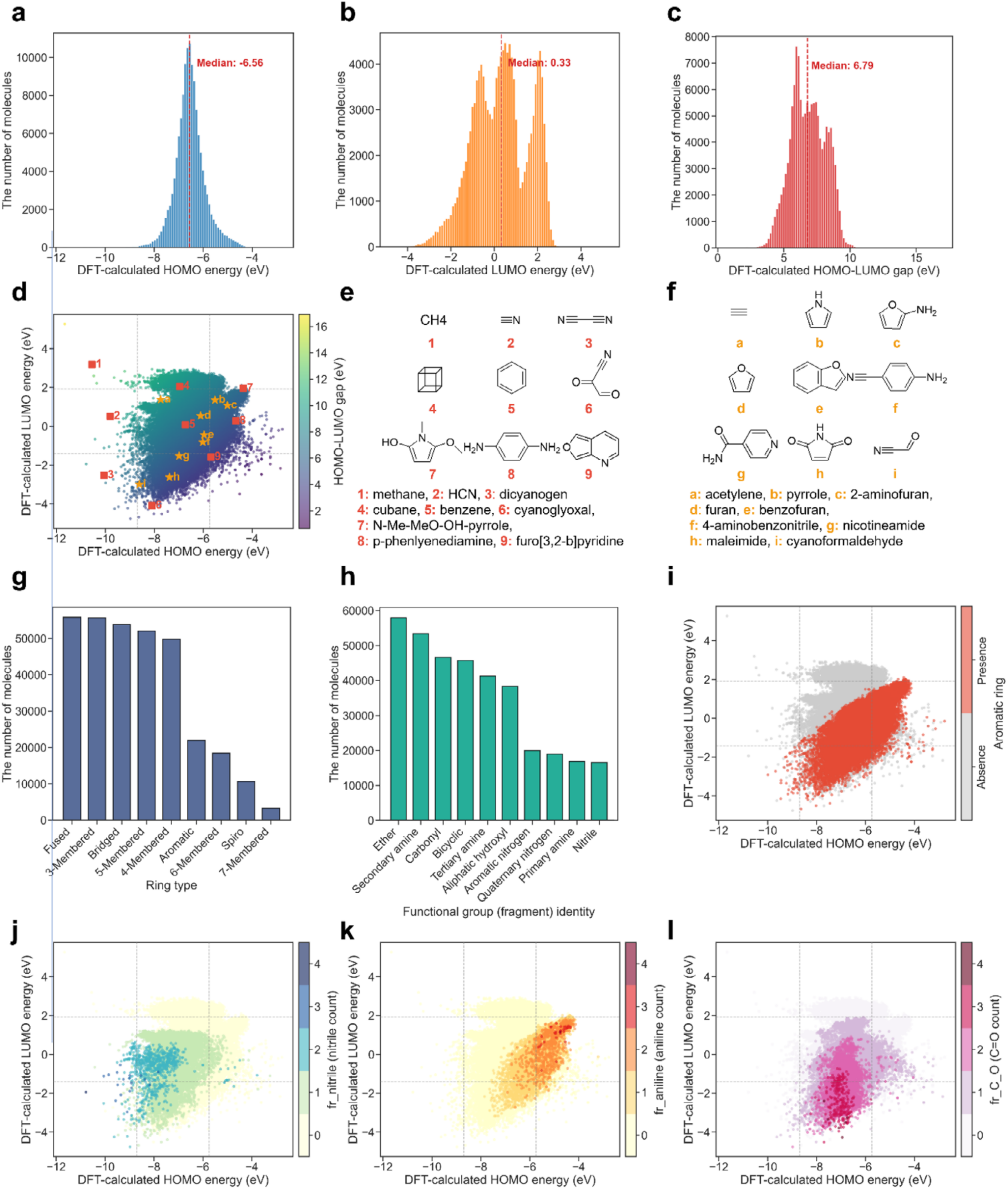
Statistical Diversity of QM9 Dataset. Statistical distributions of 133,018 organic molecules in QM9 dataset with median values indicated by red‐dotted lines: DFT‐calculated (a) HOMO and (b) LUMO levels as well as (c) HOMO–LUMO gap. (d) Correlation between DFT‐calculated HOMO and LUMO levels in QM9 dataset color‐coded by HOMO–LUMO gap. (e) Representative molecules belonging to nine high‐medium‐low HOMO/LUMO regions in the correlation distribution and (f) benchmark molecules. Statistical distributions of 133,018 organic molecules in QM9 dataset: (g) major functional group (fragment) identities and (h) ring type occurrences. Correlation between DFT‐calculated HOMO and LUMO levels in QM9 dataset color‐coded by (i) the presence or absence of an aromatic ring and by the counts of (j) nitrile, (k) aniline, and (l) carbonyl moieties.

For selected fragments and ring identities, these initially discrete and seemingly limited structure‐property relationships become much clearer through a deeper analysis of structural diversity across the large‐scale molecular space in QM9, followed by systematic mapping of their associated electronic properties with statistical correlation metrics (Figure 1g–l, Figures S1–s10) [37]. Regarding ring identity, molecules containing aromatic rings show a clear tendency toward lower LUMO and higher HOMO energies with reduced HOMO–LUMO gaps than those lacking aromatic rings, whereas other ring‐based descriptors exhibit much more diffuse and ambiguous distributions (Figure 1i and Figure S11). The distinct trend arises from the fact that aromaticity is an electronically meaningful descriptor rather than a purely structural one. Aromatic rings directly define π‐electron delocalization through cyclic conjugation, planarity, and resonance stabilization across molecular structures [4, 38]. In contrast, other ring descriptors, such as ring size or fused topology, are structurally broader and electronically heterogeneous, as they do not uniquely distinguish between saturated, unsaturated, aromatic, heteroatom‐containing motifs. As a result, aromaticity serves as a more robust and electronically relevant descriptor of frontier orbital behaviors than ring identity alone. Similarly, fragment‐resolved property mapping on the frontier orbital landscape reveals chemically intuitive trends, particularly for nitrile, aniline, and carbonyl moieties (Figure 1j–l). Increasing the number of nitrile or carbonyl functionalities progressively shifts molecules toward more negative LUMO energies, accompanied by a concurrent stabilization of HOMO, consistent with their electron‐withdrawing characteristics (Figure 1j,l). The number of aniline substituents correlates strongly with reduced LUMO energies reflecting the electronically donating amino nature and elevated HOMO energies arising from electronically delocalized conjugation of aromatic π‐system (Figure 1k).

Consistent with the structural motif‐level trends, correlation analysis across the full QM9 property set reveals the intrinsic coupling between frontier orbital‐based energies and other computed molecular descriptors (Figure 2a). Most importantly, HOMO–LUMO gap shows a strong positive correlation with LUMO energy, exhibiting Pearson correlation coefficient of 0.9, indicating that shifting LUMO within QM9 is rarely an isolated tuning knob but is typically accompanied by a concomitant change in the gap (Figure 2b and Figure S12). Beyond purely electronic descriptors, LUMO exhibits a moderate correlation with zero‐point vibrational energy (Pearson correlation coefficient: 0.7), suggesting that frontier orbital distributions in QM9 are partially intertwined with variations in molecular size and vibrational degrees of freedom (Figure 2c). Moreover, LUMO is negatively correlated with the per‐atom energetics with Pearson correlation coefficients of −0.6, implying that molecules with deeper LUMO levels tend to be statistically associated with less stabilized atomic‐scale energetics (Figure 2d). Notably, LUMO also shows non‐negligible correlations with other molecular descriptors, such as dipole moment and heat capacity. Consequently, these observations emphasize that reliable inverse design for target HOMO/LUMO windows requires models that can capture multivariate, non‐additive structure–property couplings without relying on single structural motif heuristics. This motivates AI framework developed in this study to generalize design rules learned from QM9 toward much larger molecular libraries.

**FIGURE 2:**
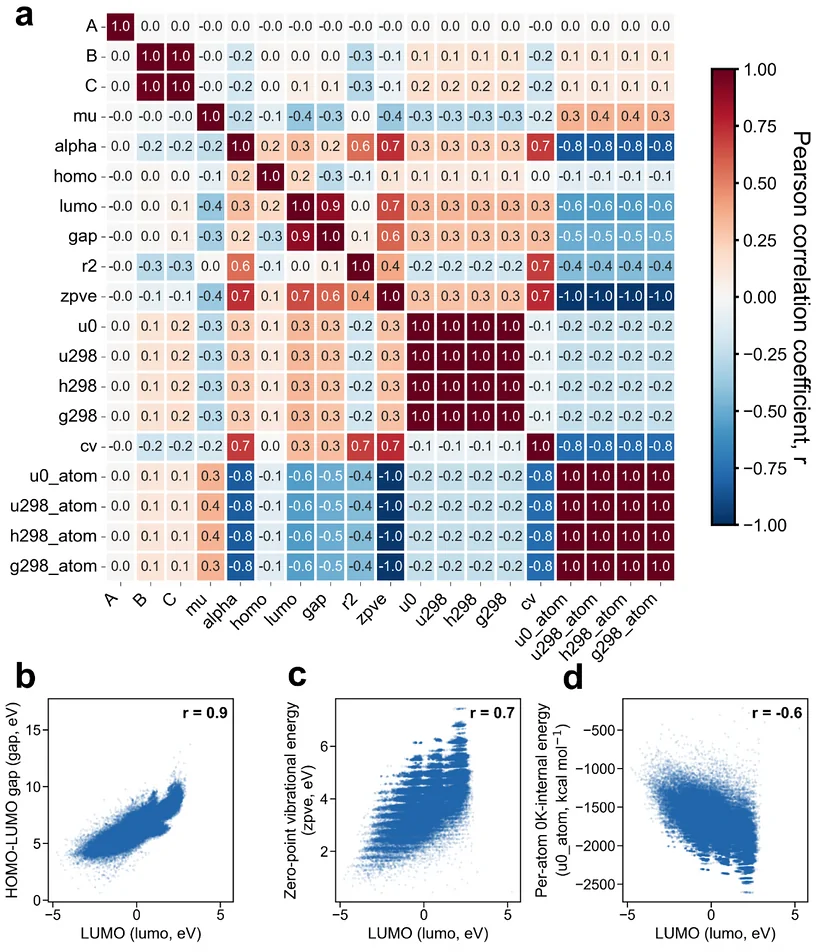
Full Heatmap of Descriptor Correlations in QM9 Dataset. (a) Pairwise Pearson correlation coefficients of 19 DFT‐calculated descriptors embedded in QM9 dataset. Distributions of (b) HOMO–LUMO gap, (c) zero‐point vibrational energy, and (d) per‐atom 0 K‐internal energy highly correlated with LUMO for 133,018 organic molecules in QM9 dataset. Notation of labels: rotational constants (A, B, C), dipole moment (mu), isotropic polarizability (alpha), HOMO (homo), LUMO (lumo), HOMO–LUMO gap (gap), electronic spatial extent (r2), zero‐point vibrational energy (zpve), internal energy at 0 K (u0), internal energy at 298 K (u298), enthalpy at 298 K (h298), Gibbs free energy at 298 K (g298), heat capacity at 298 K (cv), and corresponding per‐atom energies at 0 K (u0_atom) and 298 K (u298_atom, h298_atom, g298_atom).

### 2.2 Toward Scalable Frontier Orbital Prediction: AI Model Training and Validation

Building on this dataset foundation, we develop and benchmark a selected set of AI models for predicting HOMO and HOMO–LUMO gaps directly from molecular structures with no computational calculations. Notably, LUMO value is indirectly determined from the AI‐predicted values of HOMO and HOMO–LUMO gap. We systematically evaluate multiple molecular representations (e.g., RDKit‐based fragment descriptors; Tables S1–S4) and learning paradigms from conventional descriptor‐based regressors to deep‐learning models under consistent training/validation protocols and quantify their predictive accuracy and generalization. A best‐performing model is finally used as an engine for large‐scale inference, enabling inverse design and rapid candidate prioritization in ultra‐large virtual libraries.

The curated QM9 dataset comprising 133 018 molecules was randomly divided using a fixed random seed of 42 into a training subset of 120 000 molecules (90.2%) and a validating subset of 13 018 molecules (9.8%) (Figure 3). This approximately 90:10 partition was selected to maximize the amount of data available for learning the diverse molecular structure–property relationships represented in QM9, while retaining a substantial number of molecules (13 000 molecules), that were not used for parameter optimization during model fitting and could therefore be used consistently for model selection, early stopping where applicable, and performance comparison across the different model architectures. The identical partition was applied to all models, ensuring that differences in predictive performance arose from the model architecture and molecular representations rather than from differences in the sampled data. Moreover, the training and validating subsets exhibit closely matched distributions of HOMO, LUMO, and HOMO–LUMO gap relative to the complete curated QM9 dataset, indicating that the random partition preserves the overall electronic‐property distribution of QM9.

**FIGURE 3:**
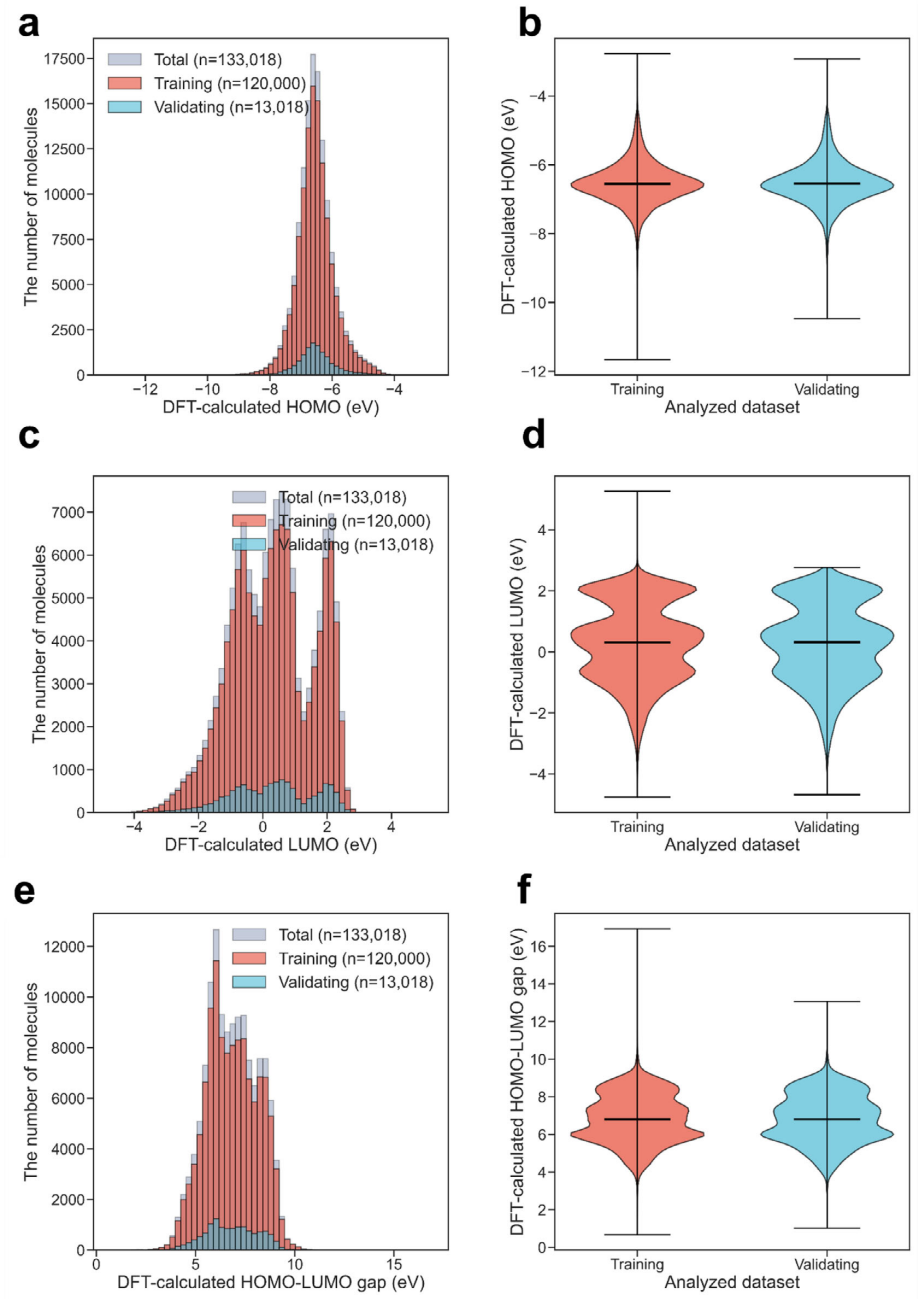
Statistically Reasonable Diversity of Training and Validating Subsets. Statistical distributions of DFT‐calculated (a, b) HOMO, (c, d) LUMO, and (e, f) HOMO–LUMO gap values for 133,018 organic molecules in QM9 dataset and its subsets (i.e., training and validating subsets).

Comparative training and validation of five different models (directed message passing neutral network (DMPNN), chemical bidirectional encoder representations from transformers (ChemBERTa), light gradient boosting machine (LightGBM), extreme gradient boosting (XGBoost), and categorical boosting (CatBoost)) identify DMPNN as the best‐performing model for frontier orbital prediction, delivering the most favorable metrics for both the training subset (coefficient of determination (*R^2^*) = 0.99; root mean squared error (RMSE) < 0.12; mean absolute error (MAE) < 0.06) and validating subset (*R^2^* > 0.95; RMSE < 0.22; MAE < 0.12) (Figure 4 and Figures S13–S21) [33, 34, 39, 40, 41, 42]. Notably, deep‐learning model training is terminated by early stopping when the validation loss function (MSE in this study) fails to decrease further for consecutive epochs after reaching its minimum value (Figure S22). In contrast, gradient‐boosting models follow decision tree‐based regression processes instead of involving epoch‐wise optimization. Each model is trained in over 50 hyperparameter optimization trials, where a tree‐like structure is formed recursively splitting data according to threshold values of input features, with a final parameter set selected based on the validation performance.

**FIGURE 4:**
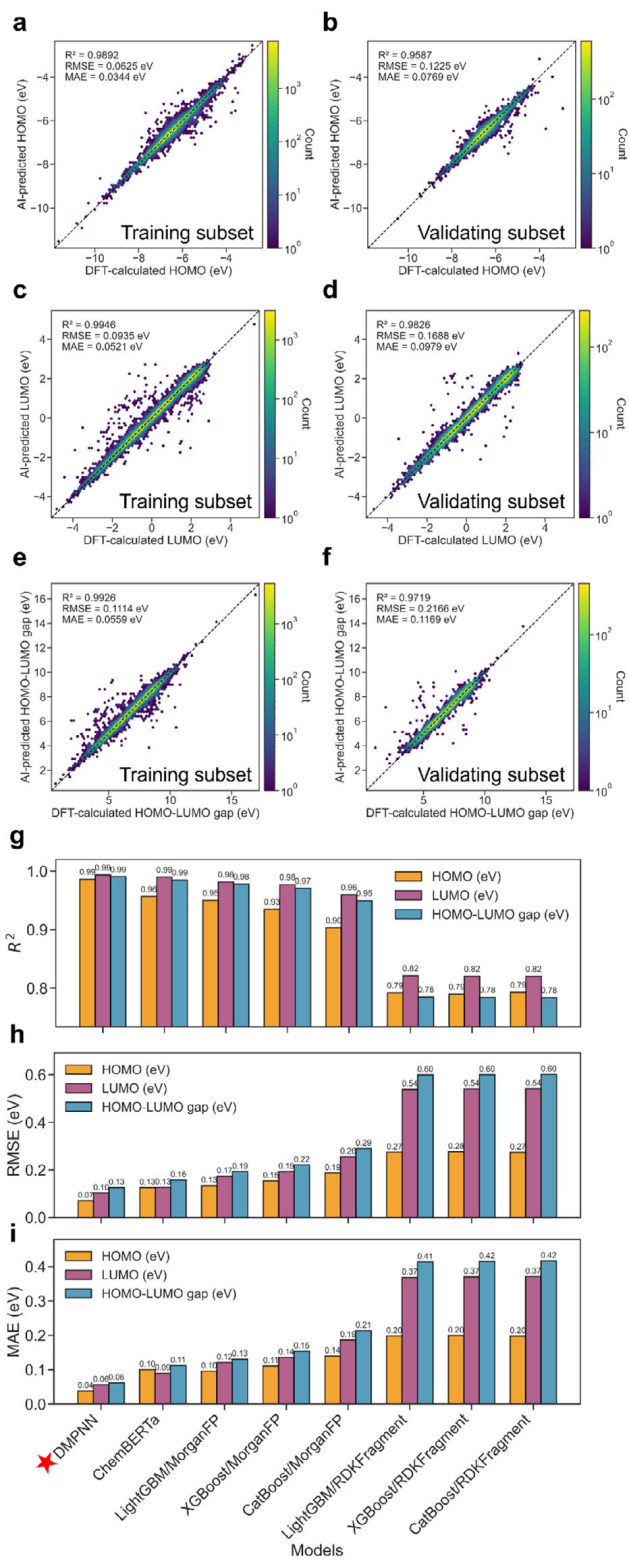
Performance Analyses of Graph‐Based DMPNN Model. AI‐predicted values of (a, b) HOMO, (c, d) LUMO, and (e, f) HOMO–LUMO gap compared with corresponding DFT‐calculated values for 133,018 organic molecules in (a, c, e) training and (b, d, f) validating subsets. The dashed line represents the ideal party (*y* = *x*). Model performance is quantified by three evaluation parameters: Coefficient of determination (R^2^), root mean squared error (RMSE), and mean absolute error (MAE). Performance comparison of QM9 dataset for all the models employed in this study based on the evaluation parameters: (g) R^2^, (h) RMSE, and (i) MAE, with red‐filled star symbol for top‐performing DMPNN model.

To evaluate the transferability of the optimized DMPNN model beyond the internal QM9 validation, we further test it using three independent quantum‐chemical datasets: PubChem Quantum Chemistry (PubChemQC), Organic Electronics 62k (OE62), and Quantum‐Mechanical Properties of Drug‐like Molecules (QMugs) [43, 44, 45]. Molecules overlapping with QM9 are excluded, and molecules composed of C, H, N, O, and F are retained because these are the atom types represented during model training. This elemental restriction allows us to evaluate transferability to previously unseen molecular structures without introducing additional extrapolation to unlearned atom types and their associated bonding environments. For PubChemQC, direct comparison with the dataset‐native values yields MAEs of 0.289, 0.394, and 0.450 eV, RMSEs of 0.465, 0.646, and 0.727, and R^2^ values of 0.622, 0.748, and 0.687 for HOMO, LUMO, and the HOMO–LUMO gap, respectively (Figure S23a–c). Because OE62 and QMugs are generated using exchange–correlation functionals different from that employed for QM9, 110 molecules from each dataset are recalculated at the B3LYP/6‐31G(2df,p) level to enable method‐consistent evaluation. The resulting comparisons yield MAEs of 0.260–0.314 eV for HOMO, 0.426–0.486 eV for LUMO, and 0.572–0.596 eV for the HOMO–LUMO gap, with corresponding RMSEs of 0.360–0.455, 0.667–0.838, and 0.818–0.908, respectively (Figure S23d–i). The QM9‐trained DMPNN model is additionally confirmed to reliably predict the DFT‐calculated frontier orbital energies of experimentally reported organic donor units, yielding MAEs of 0.132, 0.144, and 0.150 eV, RMSEs of 0.188, 0.190, and 0.192, and R^2^ of 0.862, 0.843, and 0.884 for HOMO, LUMO, and the HOMO–LUMO gap, respectively (Figure S24). These results demonstrate that the model retains meaningful predictive capability for molecular structures absent from QM9 when evaluated using a consistent quantum‐chemical reference protocol.

To further assess the experimental relevance of the predicted frontier orbital trends, the AI predictions are compared with experimentally reported ionization energy (IE) and electron affinity (EA) obtained from the NIST Chemistry WebBook [46]. Frontier orbital energies are physically related to molecular electron‐removal and electron‐addition energetics, although exact correspondence between HOMO and –IE or between LUMO and –EA is not generally expected because Kohn–Sham orbital energies and experimental charged excitation energies are not strictly identical physical quantities [47, 48]. Nevertheless, the AI‐predicted HOMO energies exhibit a strong correlation with –IE for 60 experimentally characterized molecules (R^2^ of 0.918; Spearman rank coefficient of 0.926), while the AI‐predicted LUMO energies show a meaningful correlation with –EA for 48 molecules (R^2^ of 0.705; Spearman rank coefficient of 0.878) (Figure S25). Notably, substantial fractions of these molecules are absent from QM9, further supporting the transferability of the learned frontier‐orbital trends beyond the training chemical space. These results provide an experimentally anchored assessment showing that the AI model reproduces the relative molecular trends observed in experimental electron‐removal and electron‐addition energetics. Direct experimental realization of the screened candidates through molecular synthesis, device fabrication, and comprehensive photovoltaic evaluation represents an important subsequent stage following this work.

To move beyond prediction accuracy and extract chemically interpretable design rules, we next compare model explanations across conventional boosting and deep learning frameworks using a unified interpretability workflow (Figure 5 and Figures S26–S34; Table S5). In the boosting models, SHAP analysis is applied to RDKit fragment descriptors and Morgan fingerprints, whereas in the DMPNN and ChemBERTa models, fragment‐importance maps are obtained from graph‐ and sequence‐based perturbation analyses, respectively. Notably, SHAP and fragment values quantify how much each input feature contributes, positively or negatively, to shifting a model prediction away from the average prediction. Despite the clear differences in the molecular representation, these approaches reveal notably convergent trends in the resulting importance patterns. For AI‐assisted HOMO prediction, carbonyl‐, nitrile, aniline‐, halogen‐, halide‐, and imidazole‐related environments repeatedly emerge as influential motifs mostly for a negative shift, indicating that HOMO modulation is governed by a context‐dependent interplay of electron‐withdrawing effects, donor character, conjugation, and local connectivity. This is well aligned with the earlier statistical analysis of QM9, showing that frontier orbital energies cannot be rationalized by a single descriptor or fragment alone. A similarly coherent but more polarized trend is observed for AI‐assisted HOMO–LUMO gap prediction. Across the descriptor‐, fingerprint‐ graph‐, and sequence‐based analyses, the dominant contributors shift more clearly toward strongly polarizing and electron‐withdrawing motifs, including carbonyl‐, nitrile‐, tetrazole‐, ketone‐, and halide‐related substructures, mostly leading to a negative shift. Importantly, the recurrence of chemically similar and meaningful motifs across all the interpretability schemes indicates that the learned structure–property relationships are intrinsic to the chemical space of QM9 dataset, not artifacts of a specific model class.

**FIGURE 5:**
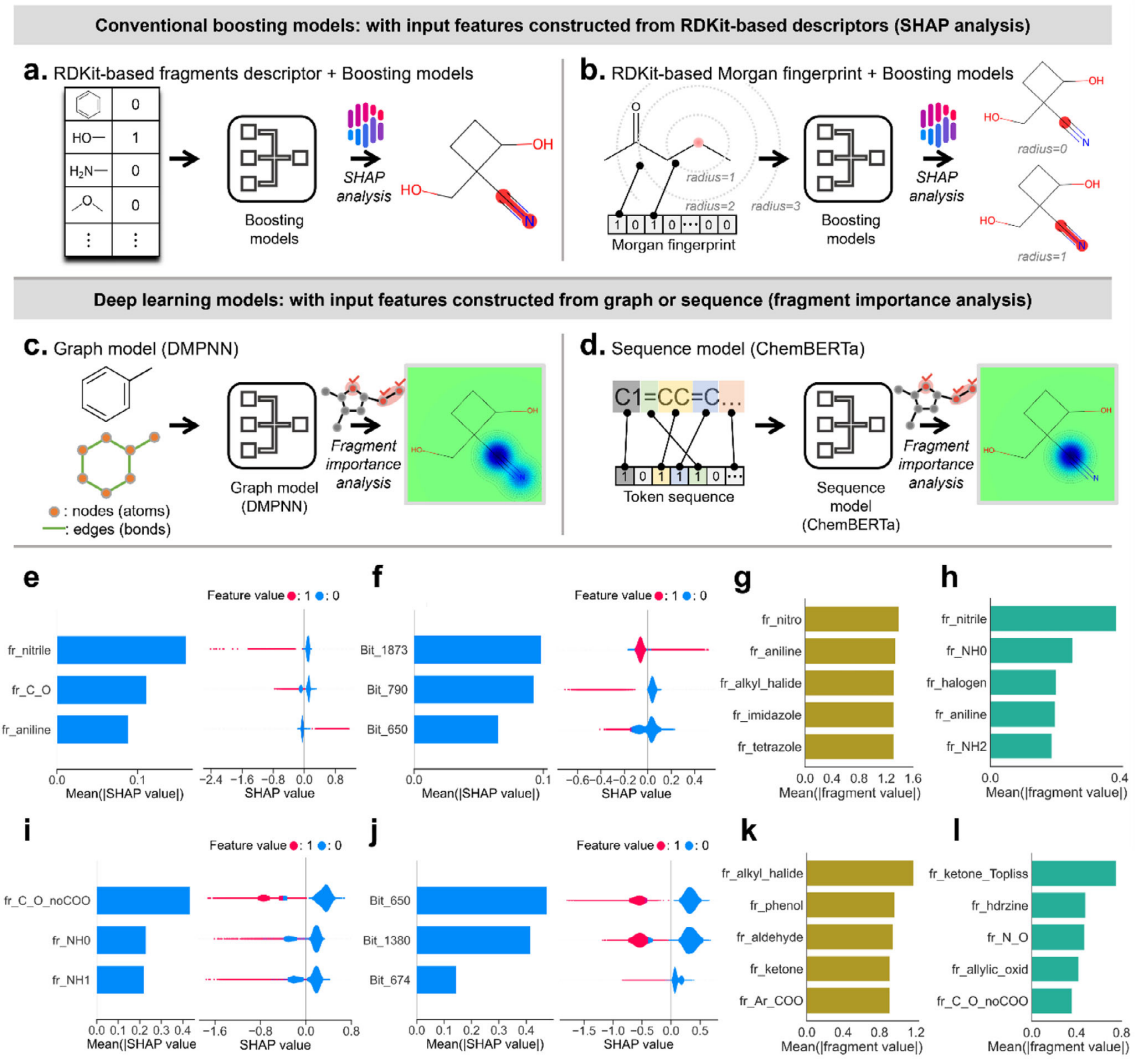
Unified Model Framework with Interpretability Analyses. Schematic illustration of each model framework: (a, b) Conventional boosting models trained with input features constructed from (a) RDKit‐based fragment descriptors or (b) RDKit‐based Morgan fingerprint, followed by SHAP analysis. (c, d) Deep learning models trained with input features constructed from (a) chemical graph or (b) sequence, followed by fragment importance analysis. Interpretability analyses of (e‐h) HOMO and (i‐l) HOMO–LUMO gap for QM9 dataset: SHAP analysis derived from (e, i) RDKit‐based fragment descriptors or (f, j) Morgan fingerprint‐based bit IDs (Table S5) for LightGBM as well as fragment importance analysis derived from (g, k) chemical graph for DMPNN or (h, l) sequence for ChemBERTa. Mean absolute SHAP values of the top three functional group (fragment) descriptors or Morgan fingerprint‐based bit IDs contributing to (e, f) HOMO or (i, j) HOMO–LUMO gap prediction, along with the corresponding SHAP value distributions for individual molecules. Mean absolute fragment values of the top five functional group (fragment) descriptors contributing to (g, h) HOMO or (k, l) HOMO–LUMO gap prediction.

### 2.3 Extension of DMPNN‐Based Ultra Large‐Scale Screening to GDB‐13

The top‐performing optimized DMPNN model is further employed to predict HOMO values and HOMO–LUMO gaps for organic molecules in GDB‐13 database, a systematically enumerated chemical universe database comprising 977,468,314 synthetically feasible small organic molecules containing up to 13 non‐hydrogen atoms of C, N, O, S, and Cl. To remain within the elemental domain represented in the QM dataset, molecules containing S or Cl were excluded, leaving 908 545 821 molecules for AI‐guided screening. The overall workflow for identifying donor building units is aimed at energetically matching them with ITIC, selected here as a representative benchmark non‐fullerene acceptor because its frontier orbital energies are well characterized and provide a well‐defined reference for evaluating donor–acceptor energy‐level alignment. The screening is based on the following criteria for optoelectronic applications: (i) ΔHOMO = HOMO (donor) – HOMO (ITIC) > 0, (ii) ΔLUMO = LUMO (donor) – LUMO (ITIC) > 0, (iii) donor HOMO–donor LUMO gap minimization, and (iv) donor HOMO–ITIC acceptor LUMO gap maximization for open‐circuit voltage improvement (Figure 6a). For each molecule, HOMO and HOMO–LUMO gap values are predicted using the optimized DMPNN model, and the corresponding LUMO value is obtained from their difference‐based relation. The resulting frontier orbital landscape is projected onto a correlation map between ΔHOMO and ΔLUMO (Figure 6b). This large‐scale projection reveals that most GDB–13 molecules occupy far from the desired donor–acceptor alignment window, whereas only a small subset satisfies simultaneously positive ΔHOMO and ΔLUMO values together with absorption‐relevant gaps (*E*_gap_ < 3.9 eV), implying the difficulty of achieving donor‐like energy alignment. In particular, 37 molecules fall within the visible region regime (*E*_gap_ < 3.1 eV), while 80,591 molecules candidates lie in the ultraviolet regime (3.1 eV < *E*_gap_ < 3.9 eV).

**FIGURE 6:**
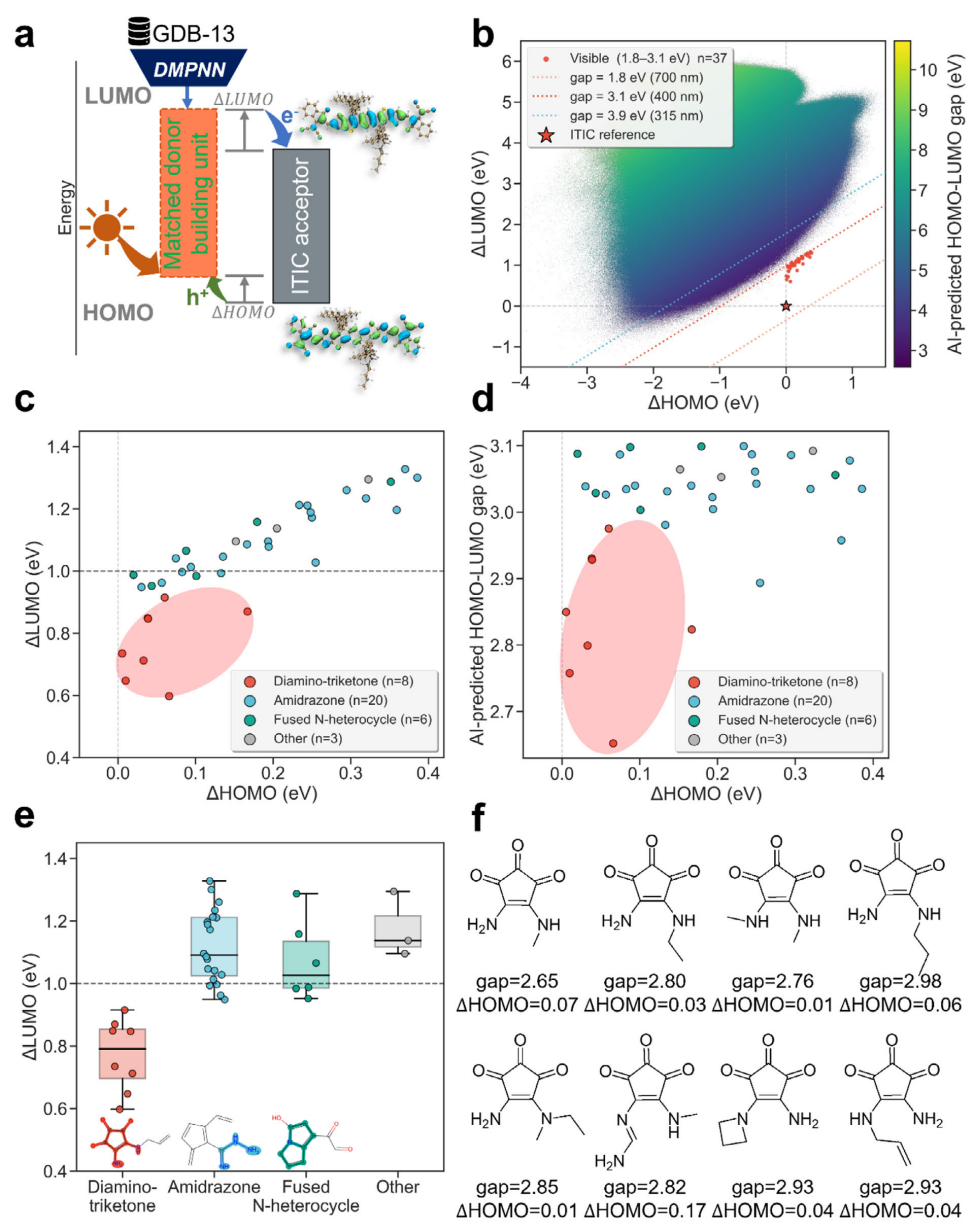
Extension of DMPNN‐Based Ultra Large‐Scale Screening to GDB–13. (a) Schematic illustration of DMPNN‐optimized energy alignment between donor candidates and ITIC acceptor, extended to the GDB–13 database. (b) Distributions of AI‐predicted frontier orbital energies of organic molecules in the GDB–13 database relative to ITIC, defined as ΔHOMO = HOMO (GDB–13) – HOMO (ITIC) and ΔLUMO = LUMO (GDB–13) – LUMO (ITIC). Dotted lines with different colors indicate HOMO–LUMO gap regions corresponding to wavelengths of 315, 400, and 700 nm, representing ultraviolet, visible, and infrared spectral regions, respectively. Correlations of ΔHOMO with (c) ΔLUMO and (d) HOMO–LUMO gap for 37 selected molecules with energy gaps corresponding to the visible light region (400 – 700 nm). These molecules are categorized into four structural classes, namely diamino‐triketone, amidrazone, fused N‐heterocycle, and others, based on structural and energetic similarities. (e) Chemical structures of eight diamino‐triketone molecules exhibiting the lowest HOMO–LUMO gaps.

To elucidate the structural origins of the 37 promising visible region candidates, we classify them into four scaffold families: diamino‐triketone, amidrazone, fused N‐heterocycle, and others (Figure 6c–e). A clear scaffold‐dependent separation emerges in the visible region‐focused correlation map between ΔHOMO and ΔLUMO (Figure 6c). Most notably, diamino‐triketone candidates cluster in the low‐ΔHOMO–low‐ΔLUMO region relative to the other families, indicating closer energetic matching to ITIC. This distinction is further supported by the gap‐ and HOMO offset‐resolved analyses. In the ΔHOMO‐gap space, diamino‐triketone candidates preferentially populate the low gap region while retaining only small positive ΔHOMO offsets (Figure 6d). This indicates that diamino‐triketone candidates satisfy both key energetic features: donor HOMO–donor LUMO gap minimization and donor HOMO–ITIC acceptor LUMO gap maximization of donor HOMO and ITIC acceptor LUMO. Likewise, the box‐plot comparison of ΔLUMO values exhibits that all eight diamino‐triketone candidates remain below ΔLUMO = 1.0 eV threshold with ΔLUMO values of 0.60 – 0.92 eV and a mean AI‐predicted gap of 2.84 eV (Figure 6e). In contrast, amidrazone class is shifted toward substantially larger ΔLUMO values, with 16 of 20 molecules located above this threshold and a mean gap of 3.03 eV. These trends indicate that the diamino‐triketone scaffold provides the most favorable compromise between narrow optical gap and acceptor‐matched energy‐level alignment among all the molecules in GDB–13 database. Further structural deconvolution highlights that the favorable compromise of the diamino‐triketone scaffold arises from a balanced hybridization of electron‐withdrawing ketone character and electron‐donating amino substituents (Figure 6f).

To directly assess the reliability of the AI‐driven predictions for GDB‐13 screening, we further perform DFT calculations at the B3LYP/6‐31G(2df,p) level for 200 candidates selected from each of the predicted HOMO energy, LUMO energy, and HOMO–LUMO gap. The DFT‐calculated values show meaningful agreement with the corresponding AI predictions, yielding MAEs of 0.254, 0.430, and 0.489 for HOMO, LUMO, and the HOMO–LUMO gap, respectively (Figure S35). These results directly confirm that the DMPNN model retains useful predictive and ranking capability for the highest‐priority molecules identified through the large‐scale screening.

To assess the DFT functional dependence of the frontier orbital energies, the 50 screened candidates are additionally evaluated using the PBE0, TPSSh, and M06 hybrid functionals with the same 6–31G(2df,p) basis set (Figure S36). Although systematic functional‐dependent shifts were limitedly observed in the absolute frontier orbital energies, strong linear and rank correlations with the B3LYP functional results are retained from the evaluation. The values range from 0.979 to 0.996 for HOMO and from 0.994 to 0.997 for LUMO, with corresponding Spearman rank coefficients of 0.987–0.997 and 0.994–0.997, respectively. These results demonstrate that the relative energetic trends underlying candidate prioritization are robust against the choice of hybrid functional, although the absolute orbital energies remain slightly functional‐dependent.

Because QM9 contains molecules with up to nine heavy atoms, the molecular‐size applicability of the model to GDB–13 molecules containing 10–13 heavy atoms is further evaluated through independent B3LYP/6‐31G(2df,p) calculations (Figures S37 and S38). Comparison between nine‐heavy‐atom molecules and a pooled 10–13‐heavy‐atom set shows only slight increases in prediction error, with MAEs changing from 0.234 to 0.234 eV for HOMO, from 0.299 to 0.340 eV for LUMO, and from 0.342 to 0.422 eV for the HOMO–LUMO gap (Figure S37). Further size‐resolved validation yields R^2^ values of 0.650–0.879, Spearman rank coefficients of 0.722–0.937, and MAEs of 0.240–0.481 eV across molecules containing 10–13 heavy atoms (Figure S38). Although limited property‐dependent variations are observed, no abrupt deterioration occurs with increasing molecular size. Screened candidates therefore extend beyond the strict molecular‐size range of QM9 but remain within an empirically validated 10–13‐heavy‐atom extrapolation range. The corresponding size‐ and property‐dependent errors provide empirical estimates of the expected prediction accuracy within this range.

Taken together, these results show that billion‐scale AI screening framework not only isolates extremely rare candidates satisfying stringent orbital alignment criteria but also resolve privileged scaffold classes that recur within the selected subset (Figures S39 and S40).

## 3 Conclusions

We developed an interpretable AI framework for predicting and inversely designing frontier orbital properties of organic molecules by integrating QM9‐based learning, model interpretability, and ultra large‐scale screening. Analysis of the curated QM9 dataset showed that frontier orbital energies would be shaped by coupled effects of functionality, conjugation, and molecular topology, with chemically meaningful contributions from motifs, such as nitrile, aniline, and carbonyl environments, while being more polarized into polarizing and electron‐withdrawing motifs for HOMO–LUMO gap. These highlight that reliable design rules cannot be established from isolated descriptors alone, but instead require models that capture multivariate, non‐additive structure–property relationships.

Among the models examined, DMPNN exhibited the best predictive performance for HOMO and HOMO–LUMO gap, while interpretability analyses across descriptor‐, fingerprint‐, graph‐, and sequence‐based models consistently recovered chemically meaningful substructures. The recurrence of similar motifs across distinct model classes indicates that the learned structure–property relationships are intrinsic to the chemical space of QM9 rather than artifacts of a single representation. The optimized DMPNN model was further extended to GDB–13 database containing 977,468,314 molecules for AI‐guided screening at unprecedented scale. This screening framework enables rapid identification and prioritization of candidates through the evaluation of donor‐acceptor alignment criteria relative to representative ITIC acceptors. Only 37 molecules satisfied a complicate screening criteria across the billion‐scale chemical space, revealing the extreme rarity of simultaneously achieving favorable donor‐like energy alignment and narrow optical gaps, and identifying diamino‐triketone family as a privileged donor building unit.

Taken together, this study demonstrates that quantum‐trained AI can provide predictive accuracy, interpretable chemical insight, and practical scalability within a single framework. Beyond accelerating frontier‐orbital prediction, the present workflow shows how billion‐scale AI screening can reveal privileged scaffold classes from enormous virtual chemical spaces. The framework can also be extended to other solar‐cell acceptors by replacing the reference frontier orbital energies and redefining the corresponding donor–acceptor energy‐alignment criteria. More broadly, the integrated strategy of quantum‐chemical data, interpretable AI, and ultra‐large‐scale screening can be adapted to other organic functional materials, including organic electrode materials, organic electrolytes, and organic semiconductors, through application‐specific training subsets and screening criteria. These capabilities provide a realistic and generalizable route toward the inverse design of organic functional molecules.

## Author Contributions

**Yeongnam Ko**: conceptualization, investigation, formal analysis. **Se Jin Kim**: visualization. **Ki Chul Kim**: conceptualization, supervision, writing – review and editing, writing – original draft, funding acquisition.

## Conflicts of Interest

The authors declare no conflicts of interest.

## Supporting information




**Supporting File**: advs77414‐sup‐0001‐SuppMat.pdf.

## Data Availability

The data that support the findings of this study are available from the corresponding author upon reasonable request.
