# Supplementary material for: Quantum‐Trained AI Enables Inverse Design of Organic Frontier Orbitals at Billion‐Scale
Source: Adv Sci (Weinh). 2026 Aug 24;13(63):e77414. doi: 10.1002/advs.77414 (PMC13502361; doi:10.1002/advs.77414)
Supplement: Supplementary file 1 [file ADVS-13-e77414-s001.pdf]

# Supporting Information

***for***

Quantum-Trained AI Enables Inverse Design of Organic Frontier Orbitals at Billion-Scale

*Yeongnam Ko, Se Jin Kim and Ki Chul Kim**

Computational Materials Design Laboratory, School of Chemical, Biomolecular and Energy Engineering, Konkuk University, 120 Neungdong-ro, Gwangjin-gu, Seoul 05029, The Republic of Korea

*Corresponding authors

Emails: kich2018@konkuk.ac.kr (K. C. Kim)

# Table of Contents

*1. COMPUTATIONAL METHODS*


1.1 Dataset Construction ……………………………………………………………….....………4

1.2 Artificial Intelligence (AI) Models ……………………………..……..……...…………………….…4

1.3 Interpretability ……………………………………..……..……..……...…………………….…5

1.4 Screening-Based Inverse Design ………………..…………….……..……...…………………….…6


*2. SUPPLEMENTARY FIGURES AND TABLES*


Figure S1. Statistical Diversity of RDKit-Based Functionality Descriptors ………………..……..…8

Figure S2. Pearson Correlation Coefficient of Functionality & HOMO ……………………..…...…9

Figure S3. Determination Coefficient of Functionality & HOMO …………………………...….....10

Figure S4. Pearson Correlation Coefficient of Functionality & LUMO ..............……………………..11

Figure S5. Determination Coefficient of Functionality & LUMO …………………......................…12

Figure S6. Pearson Correlation Coefficient of Functionality & HOMO–LUMO Gap ……………..…13

Figure S7. Determination Coefficient of Functionality & HOMO–LUMO Gap ………………………14

Figure S8. Correlation Coefficients of Ring Type & HOMO ……………………………………...…15

Figure S9. Correlation Coefficients of Ring Type & LUMO …………………………………….......16

Figure S10. Correlation Coefficients of Ring Type & HOMO–LUMO Gap ………..............................17

Figure S11. Statistical Diversity of QM9 Dataset …………………………………..……………18

Figure S12. Correlations of Frontier Orbitals with Descriptors ………………………...………...…19

Figure S13. HOMO Prediction Performance I of Boosting Models ……………………....…………20

Figure S14. HOMO Prediction Performance II of Boosting Models ……………………....……...…21

Figure S15. HOMO Prediction Performance of ChemBERTa Model ……………………....……..…22

Figure S16. LUMO Prediction Performance I of Boosting Models ……………………….……..…23

Figure S17. LUMO Prediction Performance II of Boosting Models …….…………………...…..…24

Figure S18. LUMO Prediction Performance of ChemBERTa Model ……….………………..…..…25

Figure S19. HOMO–LUMO Gap Prediction Performance I of Boosting Models ………………......…26

Figure S20. HOMO–LUMO Gap Prediction Performance II of Boosting Models ………………....…27

Figure S21. HOMO–LUMO Gap Prediction Performance of ChemBERTa Model ………………....…28

Figure S22. Learning Curves of DMPNN and ChemBERTa Models ………………….………..…29

Figure S23. Transferability of QM9-Trained DMPNN Model to External Datasets ………………..…30

Figure S24. Transferability of QM9-Trained DMPNN Model to Experimental Donors …...…………31

Figure S25. IE/EA-Based Experimental Consistency of AI-Predicted Frontier Orbital Energies …...…32

Figure S26. Interpretability Analyses of Boosting Models: HOMO …………………..……………33

Figure S27. Interpretability Analyses of Boosting Models: HOMO ……………………..……………34
Figure S28. Interpretability Analyses of Deep Learning Models: HOMO ………………..………..…35
Figure S29. Interpretability Analyses of Boosting Models: LUMO ……………………...…………36
Figure S30. Interpretability Analyses of Boosting Models: LUMO ……………………...…………37
Figure S31. Interpretability Analyses of Deep Learning Models: LUMO …………………...……...…38
Figure S32. Interpretability Analyses of Boosting Models: HOMO–LUMO Gap ……………….……39
Figure S33. Interpretability Analyses of Boosting Models: HOMO–LUMO Gap ……………….……40
Figure S34. Interpretability Analyses of Deep Learning Models: HOMO–LUMO Gap ……………....41
Figure S35. Distributions of AI Prediction Errors Relative to DFT …………………………………....42
Figure S36. DFT Functional Dependence Analysis of 50 Screened Candidates ……………………....43
Figure S37. Molecular-Size Dependence Analysis …………………………………………………....44
Figure S38. Molecular-Size Dependence Analysis …………………………………………………....45
Figure S39. AI-Driven Prediction of Frontier Orbitals for GDB-13 ……………………..…………46
Figure S40. 37 Donor Candidates Identified from GDB–13 ……………...………………...…………47
Table S1. RDKit-Based Functional Group (Fragment) Descriptors ……………………..…………48
Table S2. RDKit-Based Functional Group (Fragment) Descriptors ……………………..…………50
Table S3. RDKit-Based Functional Group (Fragment) Descriptors ……………………...…………52
Table S4. RDKit-Based Functional Group (Fragment) Descriptors ……………………...…………54
Table S5. Fragment Composition of Morgan Fingerprint Bits ……………………………..………56
REFERENCES………………………………………………… ………………….…………58

# 1. COMPUTATIONAL METHODS

## 1.1 Dataset Construction

The QM9 dataset was used as the computational reference for frontier orbital learning. The raw dataset (structural information of 133,885 molecules in structure database file (SDF) format with the density functional theory (DFT)-computed properties) was curated by requiring RDKit compatibility, successful fingerprint generation, valid molecular graphs, absence of disconnected fragments, and molecular weight < 1,000, yielding 133,018 validated molecules (99.35% retention). The validated dataset was split into training (120,000) and validating (13,018) subsets.

## 1.2 Artificial Intelligence (AI) Models

We compared conventional and deep-learning pipelines using multiple molecular representations. RDKit fragment descriptors (85 functional-group descriptor features; prefix of fr_) and RDKit-based Morgan fingerprints fragments (2048-bit binary vectors; encoding hashed circular atom environments centered on each atom in the molecular graph; Morgan fingerprint radius up to 3) with a multi-output regression wrapper were used for conventional gradient-boosting regressors (light gradient boosting machine (LightGBM), extreme gradient boosting (XGBoost), categorical boosting (CatBoost)).[1-4] In contrast, graph and sequence representations were used for deep learning schemes: (i) a directed message passing neural network (DMPNN; Chemprop v1.6.1) trained on directed molecular graphs, and (ii) a chemical bidirectional encoder representations from transformers (ChemBERTa; RoBERTa-based encoder) model fine-tuned on tokenized SMILES sequences (maximum length 512).[5-8] For DMPNN model, each SMILES string was converted to a directed molecular graph through

treating atoms as nodes with atom-level features and bonds as directed edges with bond-level features for message passing. Messages are therefore propagated along directed bonds for a configurable number of steps (depth), converted to atom-level representations, aggregated into a molecule-level embedding, and decoded by a feed-forward network. All models were trained by minimizing mean squared error (MSE) and evaluated on both splits (training and validating subsets) using coefficient of determination ($R^2$), MSE, root mean squared error (RMSE), and mean absolute error (MAE). Hyperparameters were optimized via 50 Optuna Bayesian optimization trials per algorithm, with the final configuration selected based on validation performance. The AI pipeline was designed to predict the highest occupied molecular orbital (HOMO), the lowest unoccupied molecular orbital (LUMO), and HOMO–LUMO gap values of molecules in the QM9 dataset, recognizing the intrinsic coupling among HOMO, LUMO, and the gap. Accordingly, LUMO was estimated as LUMO = HOMO + (HOMO–LUMO gap), using AI-predicted quantities.

### 1.3 Interpretability

To extract chemically interpretable structure–property relationships across heterogeneous model classes, we employed a unified attribution workflow. For conventional gradient-boosting models (LightGBM, XGBoost, and CatBoost), Shapley additive explanations (SHAP) were applied to both the fragment-based descriptors and Morgan fingerprint-based features to quantify feature contributions (i.e., SHAP values) to HOMO and HOMO–LUMO gap predictions. For DMPNN, atom-level occlusion was used by masking each atom (and incident directed bond-edge features) and defining the attribution as the prediction shift relative to the unmasked baseline; atom attributions were aggregated to functional groups (fragments) using curated RDKit SMARTS fragment patterns. Notably, when an atom matched multiple

SMARTS fragment patterns, it was exclusively assigned to the highest priority pattern. For ChemBERTa, fragment-level masking was performed by removing SMARTS-defined fragments, regenerating truncated SMILES strings, and computing prediction shifts as attributions; invalid SMILES strings or disconnected molecular graphs after fragment removal were excluded from the evaluation.

**1.4 Screening-Based Inverse Design**

The top-performing DMPNN model was deployed for large-scale inference over the GDB-13 database (977,468,314 enumerated organic molecules) to predict HOMO and HOMO–LUMO gaps without additional DFT calculations. Inference was parallelized across two NVIDIA A100 GPUs. LUMO levels were estimated as the sum of the AI-predicted HOMO and HOMO–LUMO gap values. The resulting frontier orbital landscape of GDB-13 was benchmarked against ITIC, a referenced non-fullerene acceptor, to identify donor candidates with favorable energy level alignment for organic photovoltaic (OPV) donor–acceptor architectures. To ensure consistency of the reference energy scale, the frontier orbital energies of ITIC were evaluated using different basis sets, namely B3LYP/6-31G(d,p) and B3LYP/6-31G(2df,p). The latter basis set (consistent with the DFT setting of QM9 dataset) was adopted as the final reference for the large-scale computational screening process of GDB-13 database.

Based on the computed frontier orbital values of ITIC (HOMO = –5.43 eV, LUMO = –3.31 eV), we defined the relative offsets as $\Delta HOMO = HOMO_{donor} - HOMO_{ITIC}$ and $\Delta LUMO = LUMO_{donor} - LUMO_{ITIC}$. Candidate donor building units were required to satisfy $\Delta$HOMO > 0 and $\Delta$LUMO > 0, ensuring that candidate donor HOMO would lie above the acceptor HOMO (supporting open-circuit voltage) and candidate donor LUMO would lie above the acceptor LUMO (providing a driving force for charge transfer. Candidates were then

categorized into light absorption-relevant regimes based on their HOMO–LUMO gaps ($E_{gap}$): a visible-range regime ($E_{gap}$ < 3.1 eV, corresponding to λ > 400 nm) and a UV–A regime (3.1 < $E_{gap}$ < 3.9 eV, corresponding to 315 – 400 nm). This initial electronic screening yielded 37 visible-range and 80,591 UV-A-range donor building unit candidates.

## 2. SUPPLEMENTARY FIGURES AND TABLES

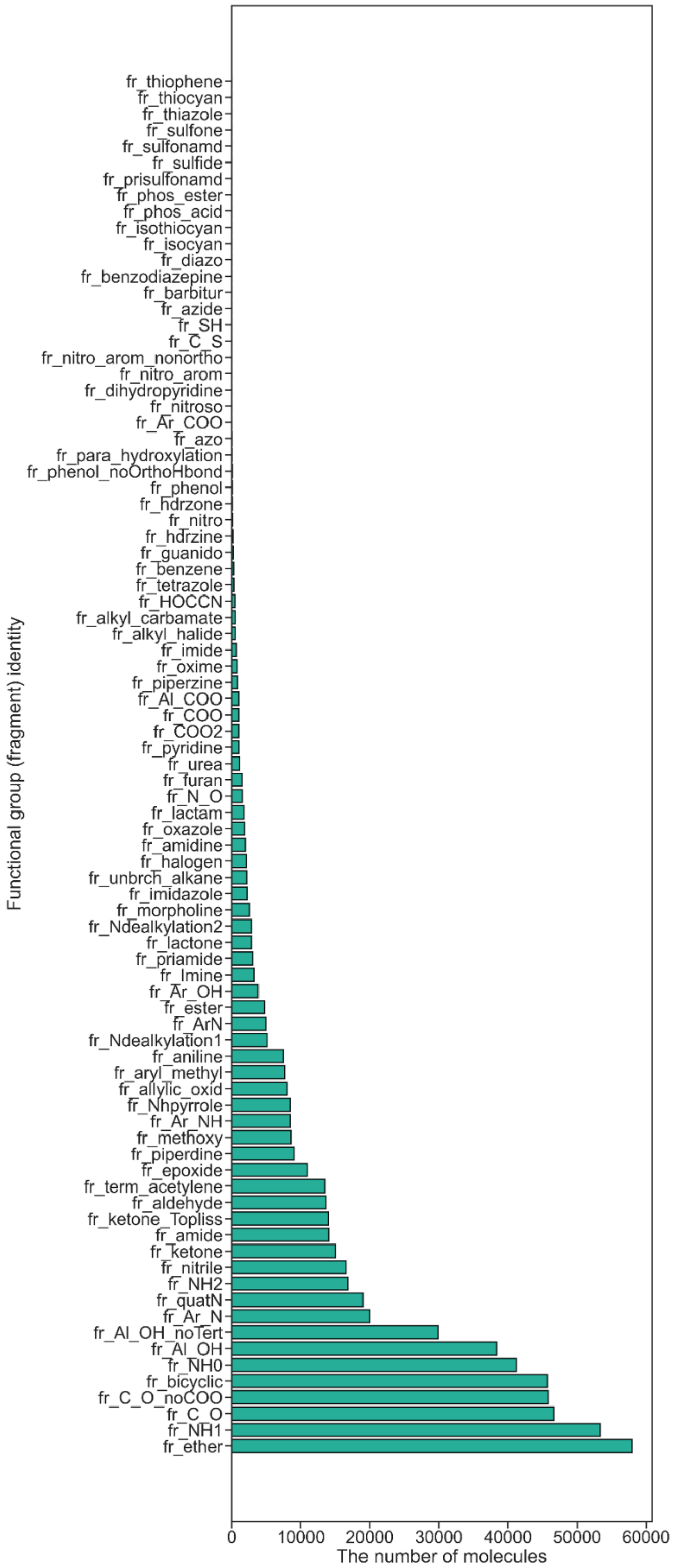


**Figure S1. Statistical Diversity of RDKit-Based Functionality Descriptors**

Statistical distributions of 133,018 organic molecules in QM9 dataset; RDKit-based functional group (fragment) descriptors.

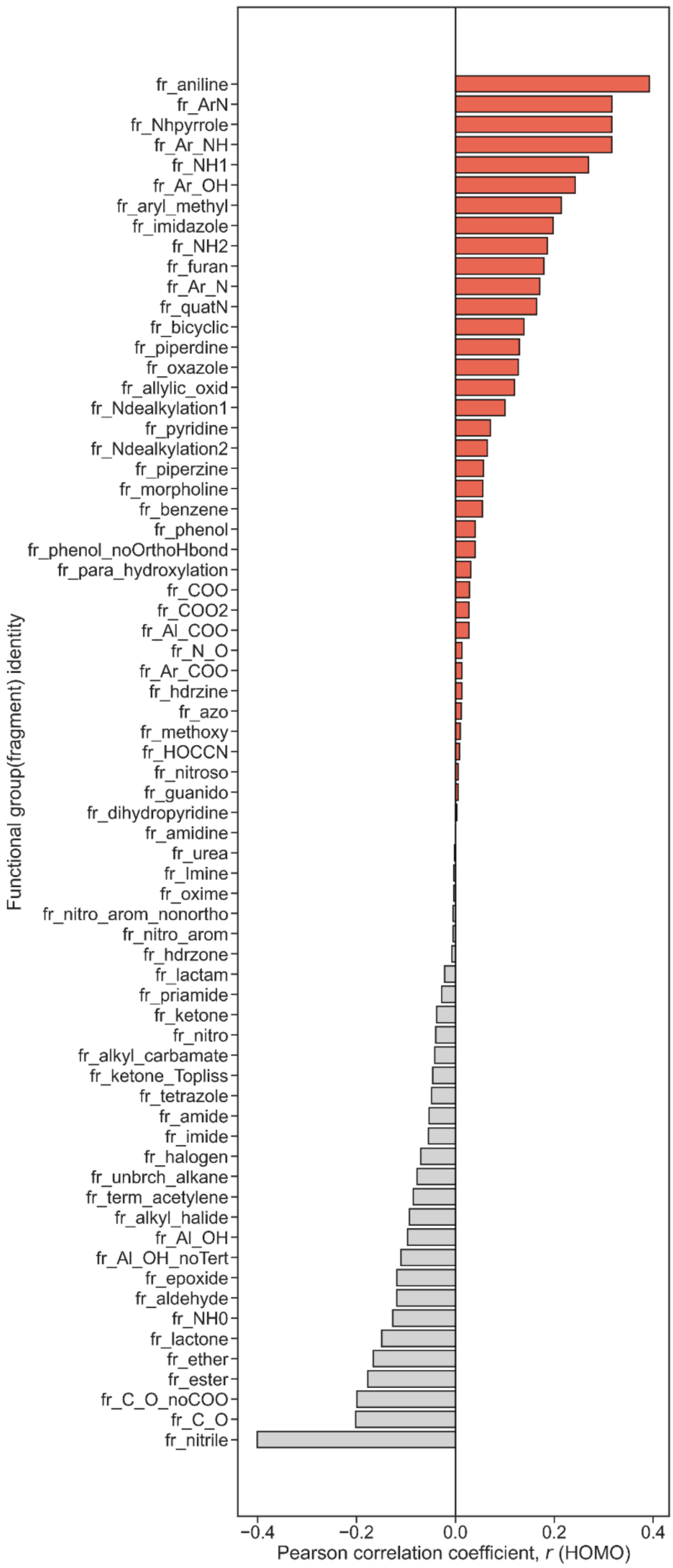


**Figure S2. Pearson Correlation Coefficient of Functionality & HOMO**

Pearson correlation coefficient of each RDKit-based functional group (fragment) descriptor correlated with HOMO levels of 133,018 organic molecules in QM9 dataset.

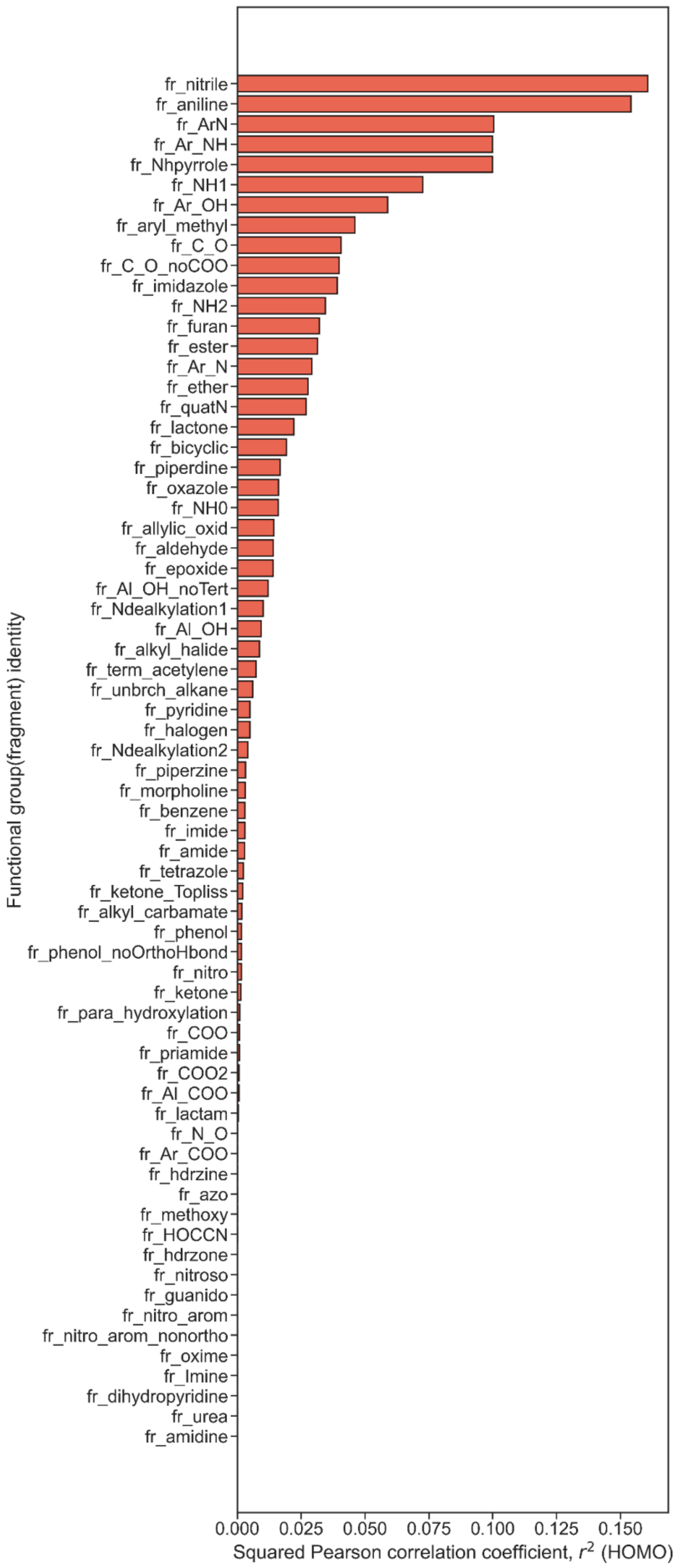


**Figure S3. Determination Coefficient of Functionality & HOMO**

Determination Coefficient of each RDKit-based functional group (fragment) descriptor correlated with HOMO levels of 133,018 organic molecules in QM9 dataset.

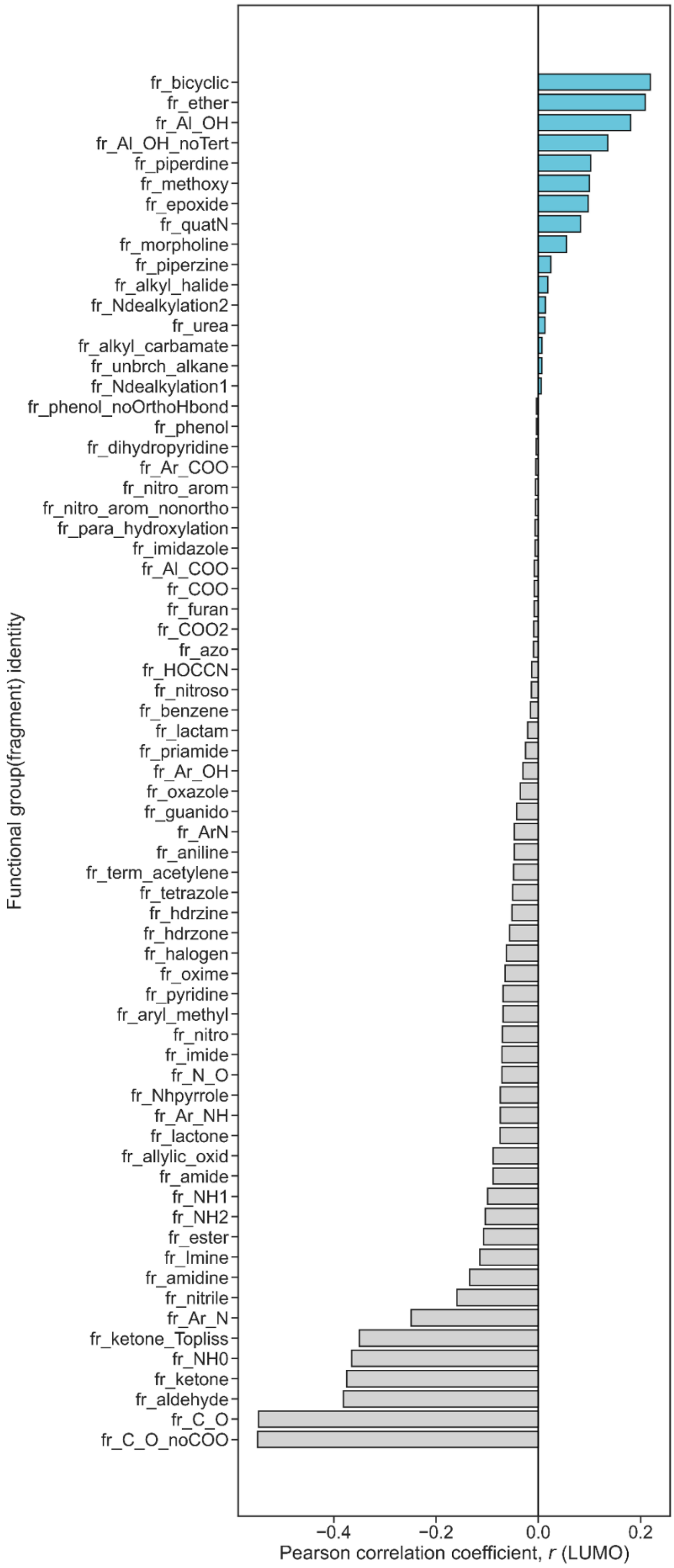


**Figure S4. Pearson Correlation Coefficient of Functionality & LUMO**

Pearson correlation coefficient of each RDKit-based functional group (fragment) descriptor correlated with LUMO levels of 133,018 organic molecules in QM9 dataset.

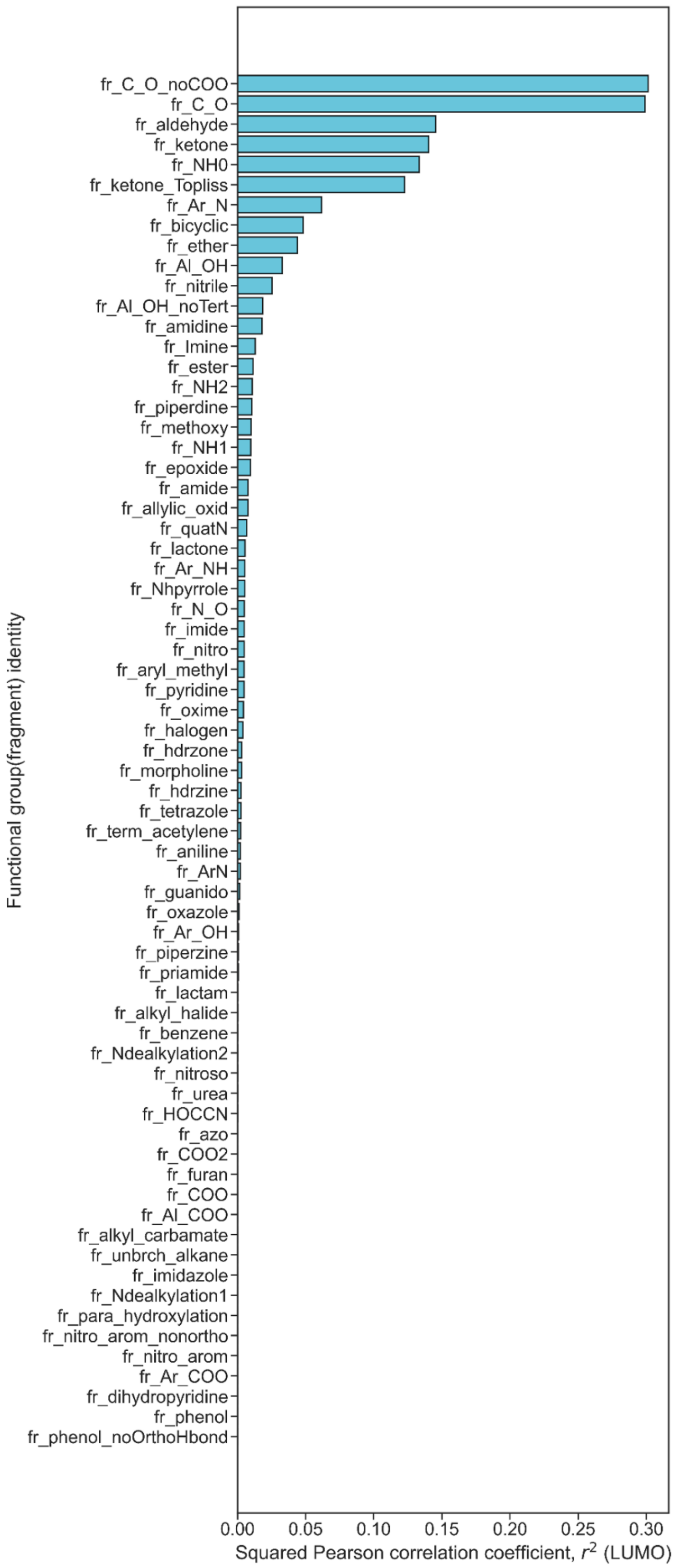


**Figure S5. Determination Coefficient of Functionality & LUMO**

Determination Coefficient of each RDKit-based functional group (fragment) descriptor correlated with LUMO levels of 133,018 organic molecules in QM9 dataset.

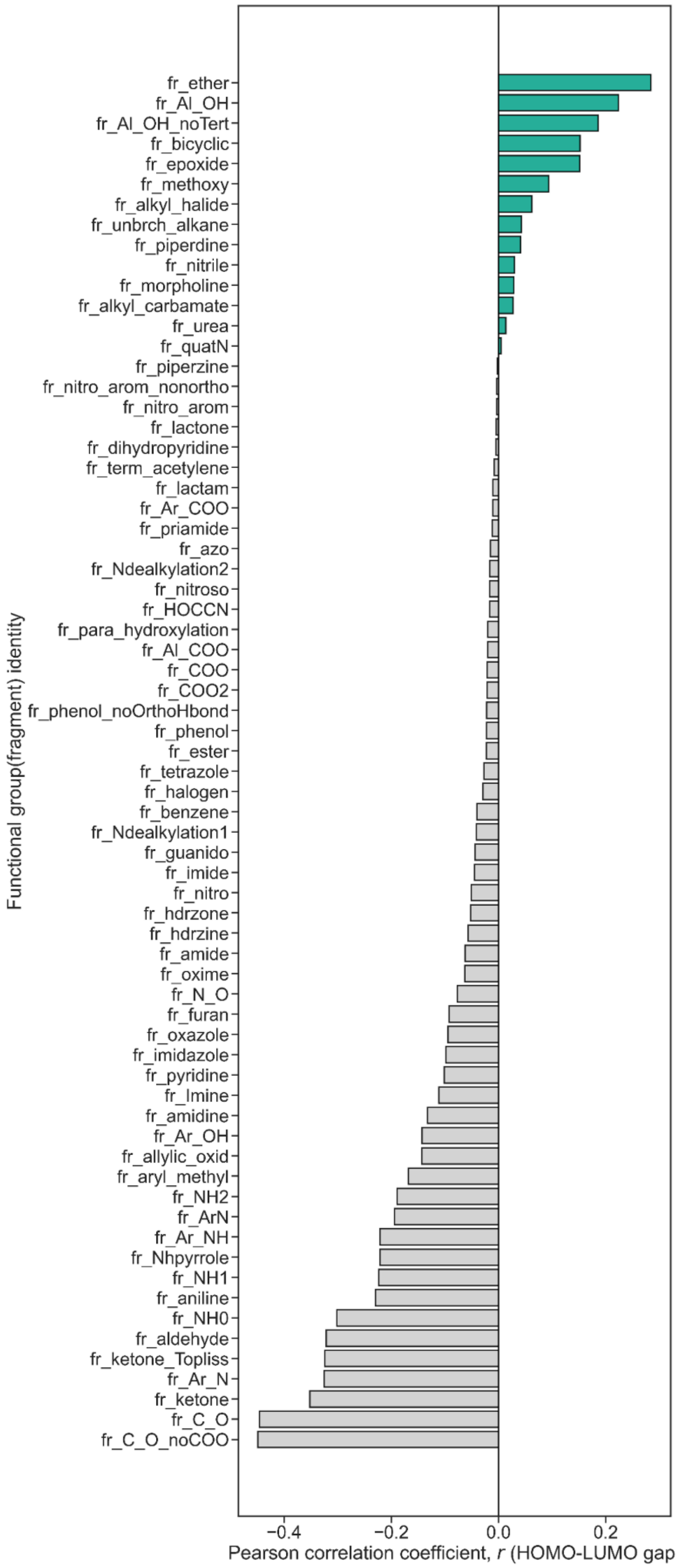


**Figure S6. Pearson Correlation Coefficient of Functionality & HOMO–LUMO Gap**

Pearson correlation coefficient of each RDKit-based functional group (fragment) descriptor correlated with HOMO–LUMO gaps of 133,018 organic molecules in QM9 dataset.

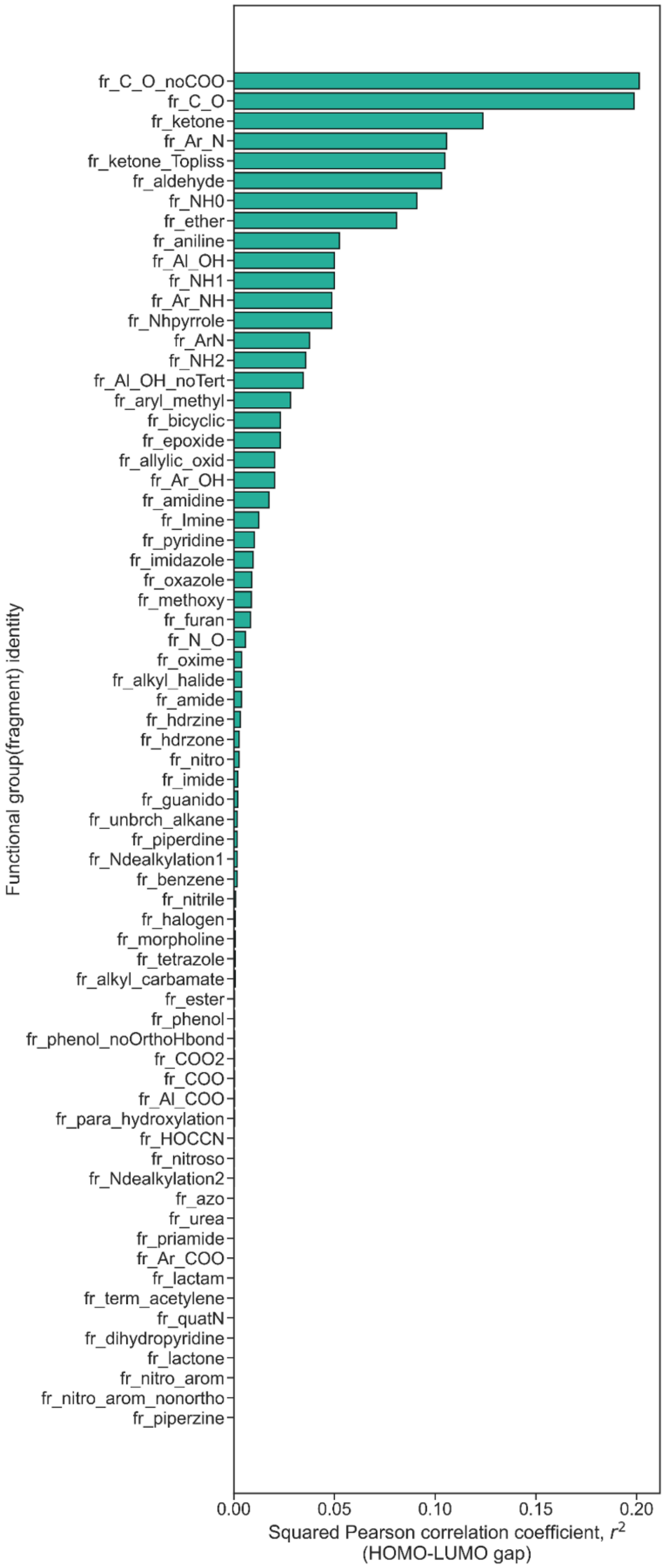


**Figure S7. Determination Coefficient of Functionality & HOMO–LUMO Gap**

Determination Coefficient of each RDKit-based functional group (fragment) descriptor correlated with HOMO–LUMO gaps of 133,018 organic molecules in QM9 dataset.

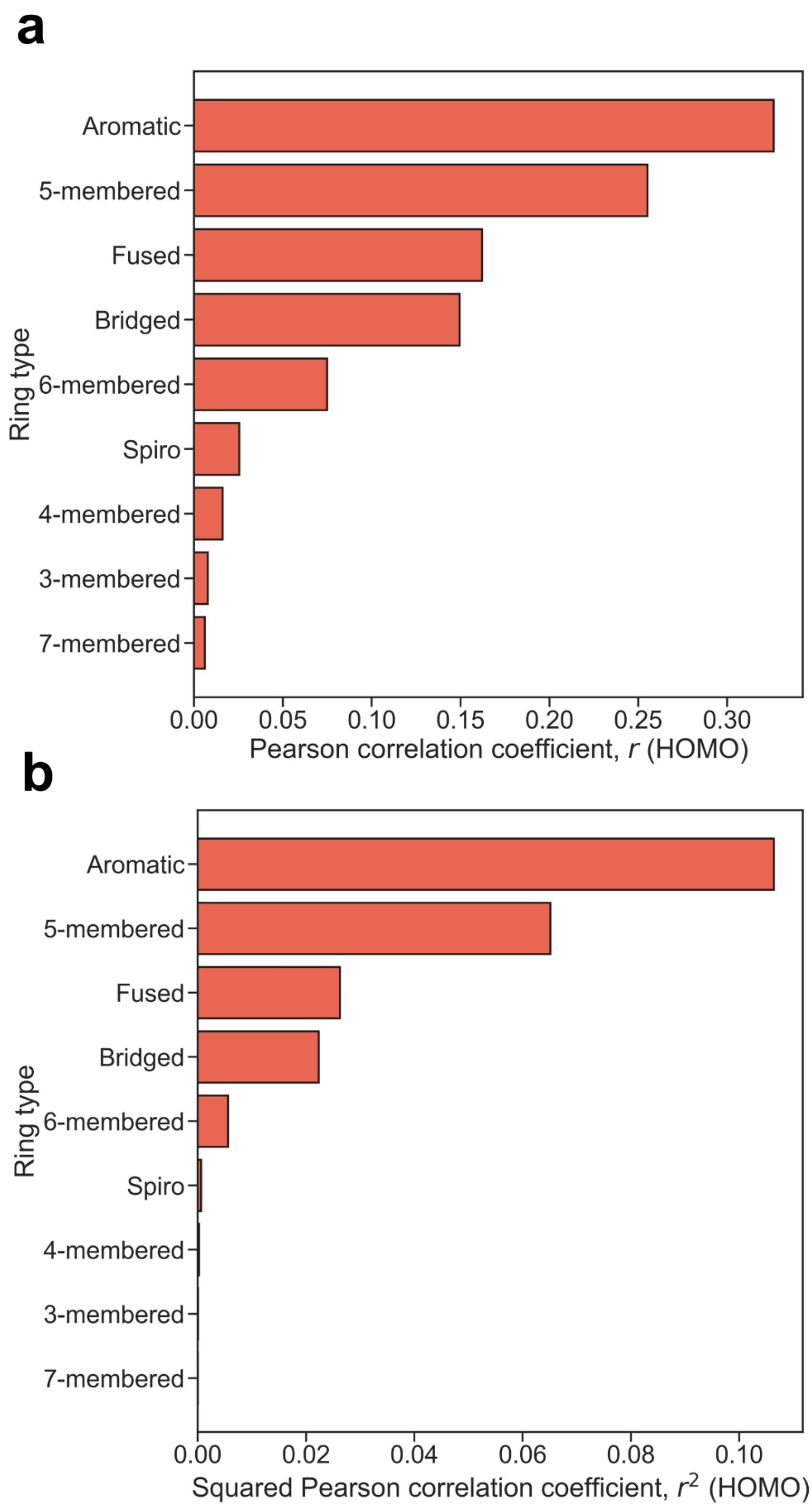


**Figure S8. Correlation Coefficients of Ring Type & HOMO**

(a) Pearson correlation coefficient and (b) determination coefficient of each RDKit-based ring type descriptor correlated with HOMO levels of 133,018 organic molecules in QM9 dataset.

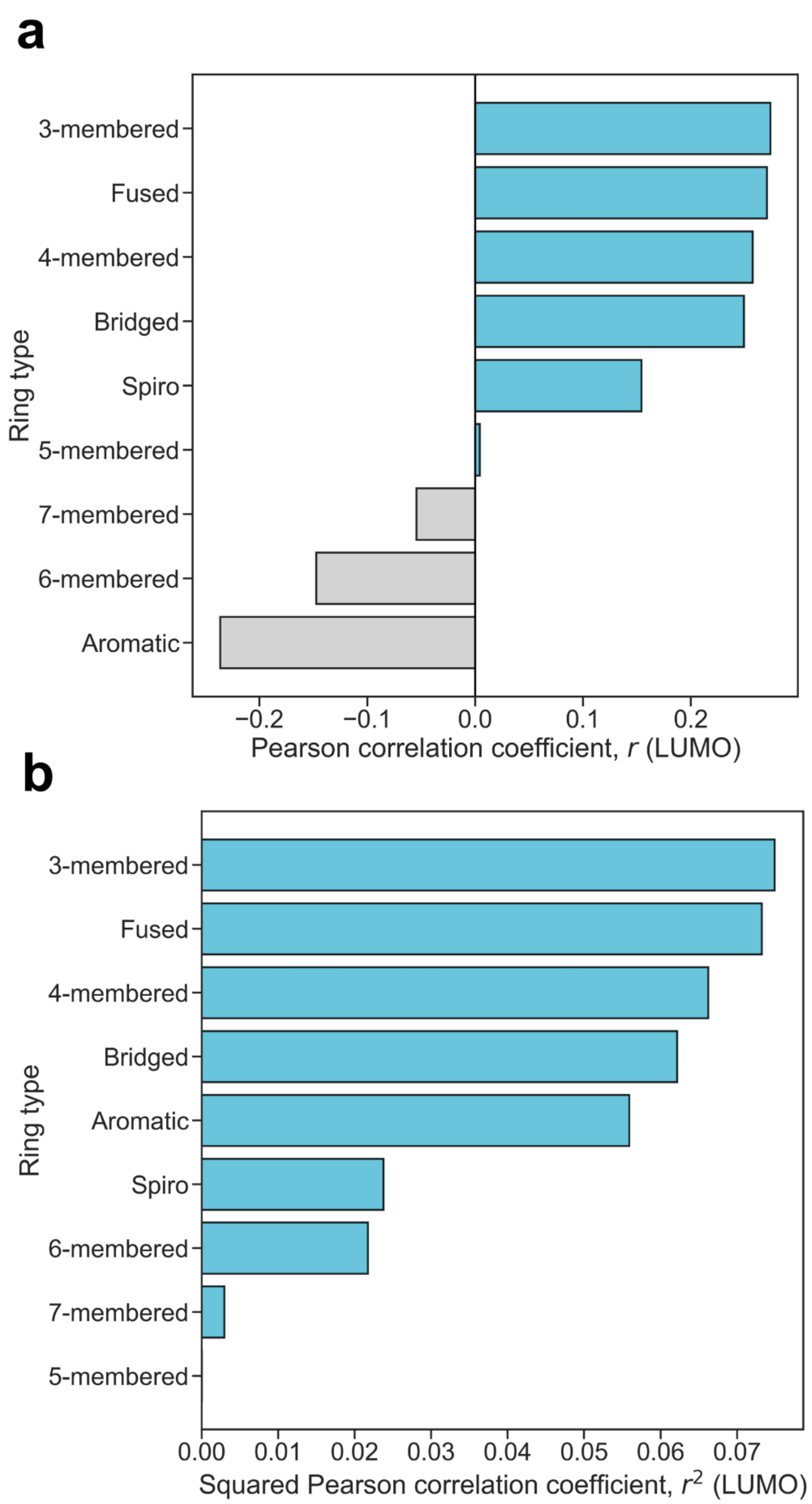


**Figure S9. Correlation Coefficients of Ring Type & LUMO**

(a) Pearson correlation coefficient and (b) determination coefficient of each RDKit-based ring type descriptor correlated with LUMO levels of 133,018 organic molecules in QM9 dataset.

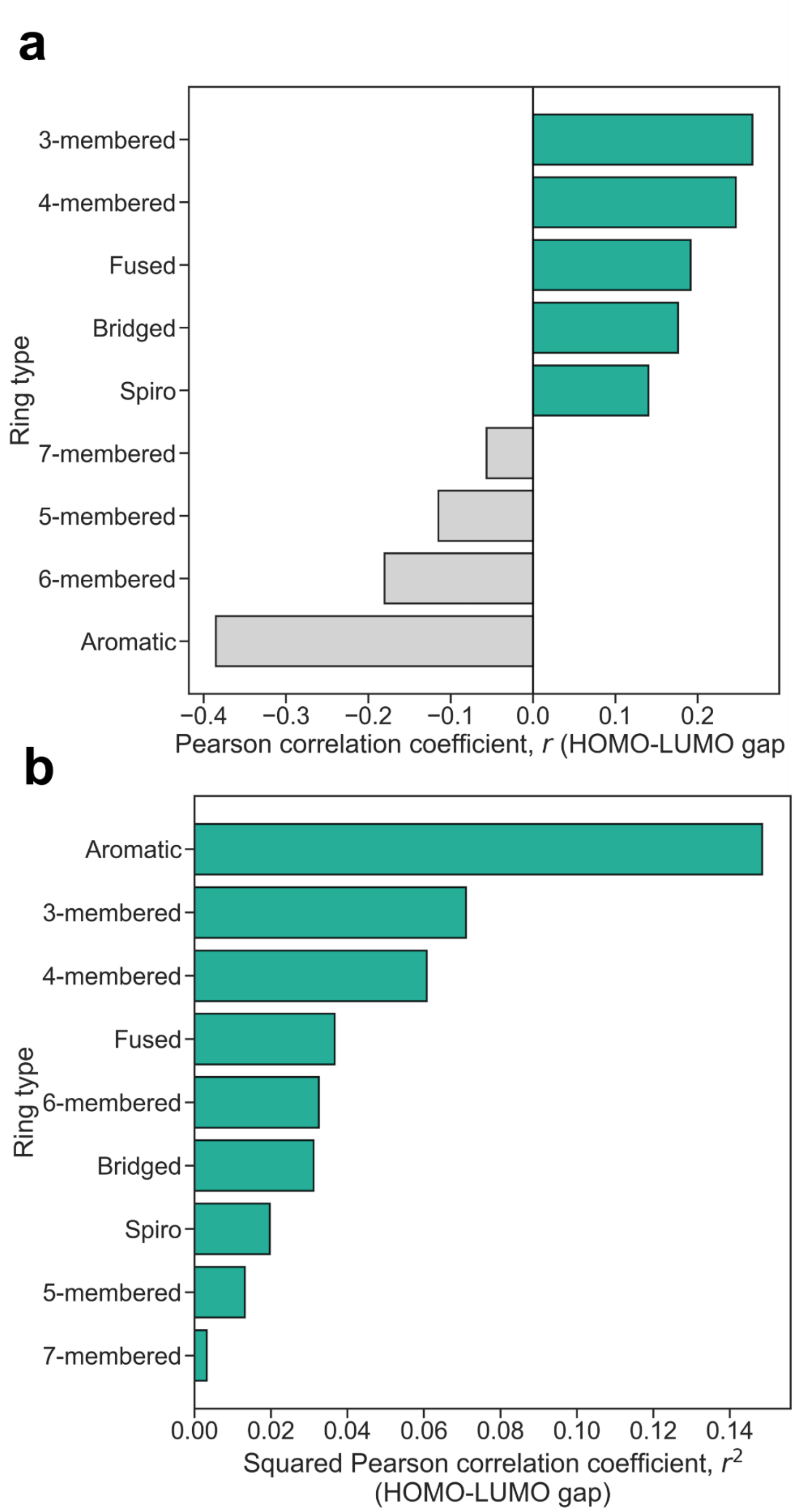


**Figure S10. Correlation Coefficients of Ring Type & HOMO–LUMO Gap**

(a) Pearson correlation coefficient and (b) determination coefficient of each RDKit-based ring type descriptor correlated with HOMO–LUMO gaps of 133,018 organic molecules in QM9 dataset.

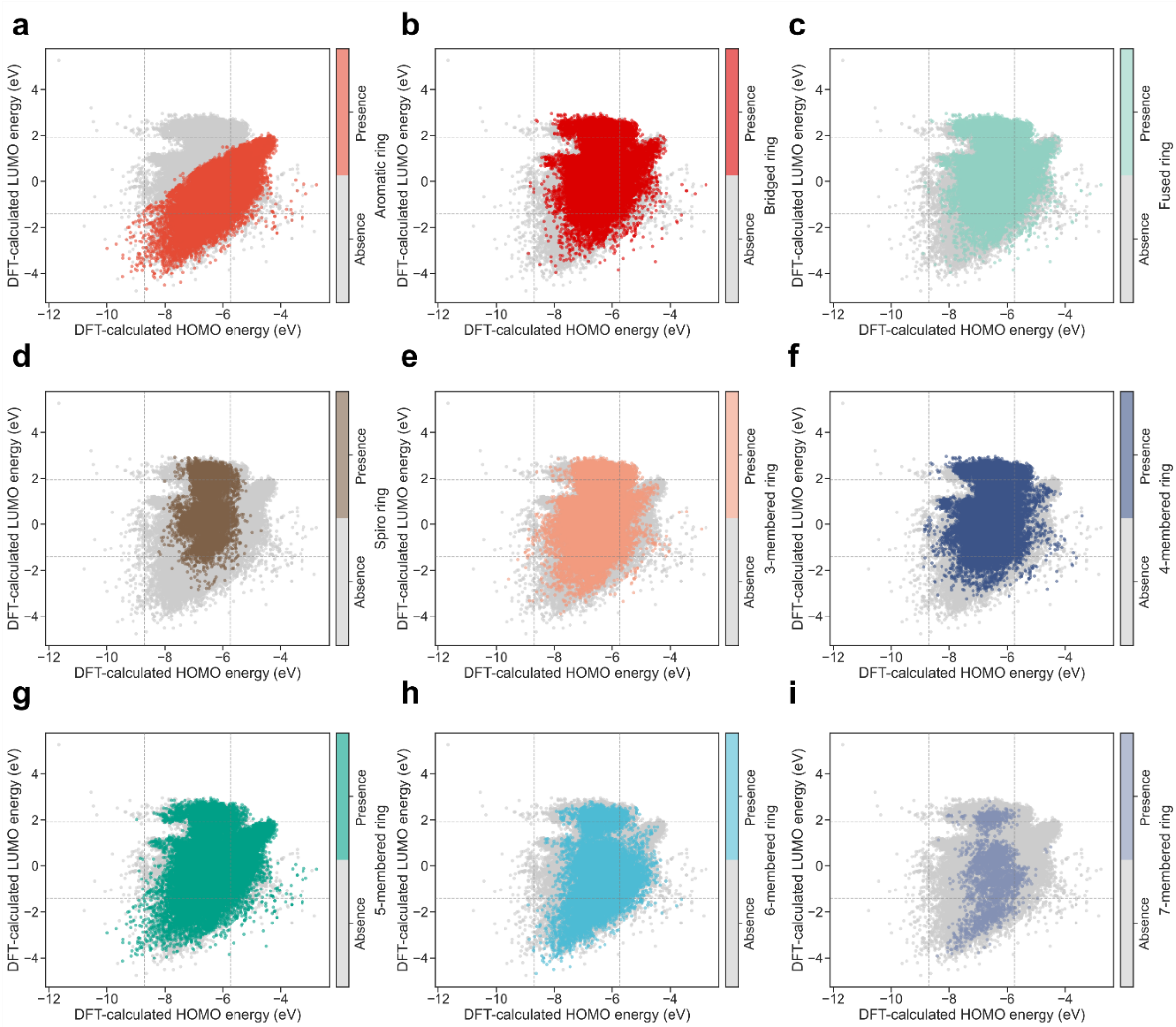


**Figure S11. Statistical Diversity of QM9 Dataset**

Correlations between DFT-calculated HOMO and LUMO levels of 133,018 organic molecules in QM9 dataset color-coded by various ring identities: (a) aromatic ring (b) bridged ring, (c) fused ring, (d) spiro ring, (e) three-membered ring, (f) four-membered ring, (g) five-membered ring, (h) six-membered ring, and (i) seven-membered ring.

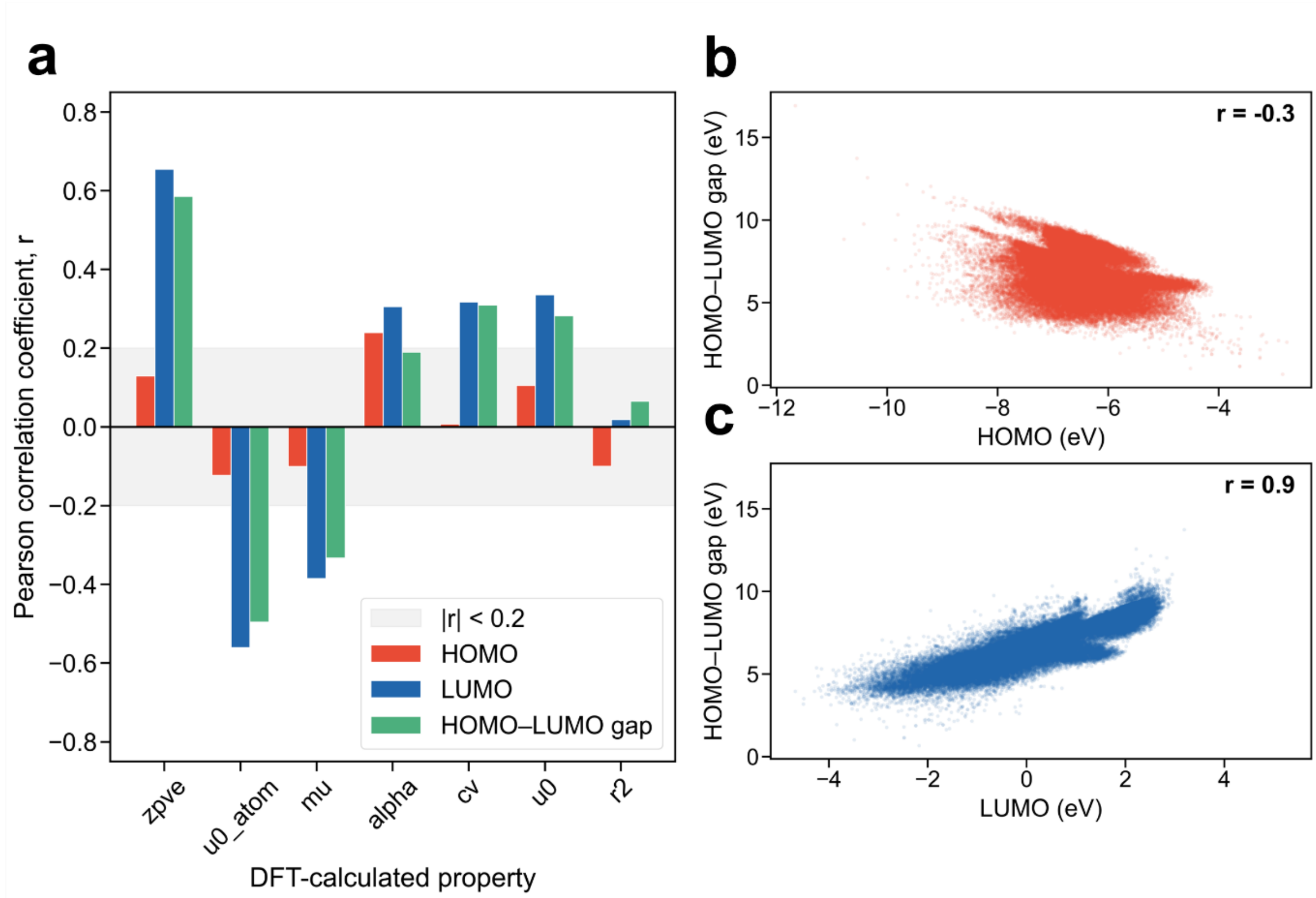


**Figure S12. Correlations of Frontier Orbitals with Descriptors**

(a) Pearson correlations coefficients of HOMO, LUMO, and HOMO–LUMO gap with other descriptors: zero-point vibrational energy (zpve), per-atom internal energy at 0 K (u0_atom), dipole moment (mu), isotropic polarizability (alpha), heat capacity at 298 K (cv), internal energy at 0 K (u0), and electronic spatial extent (r2). (b) Scatter plot showing the relationship between HOMO and HOMO–LUMO gap ($r$ = –0.3). (c) Scatter plot showing the strong positive correlation between HOMO and HOMO-LUMO gap ($r$ = 0.9).

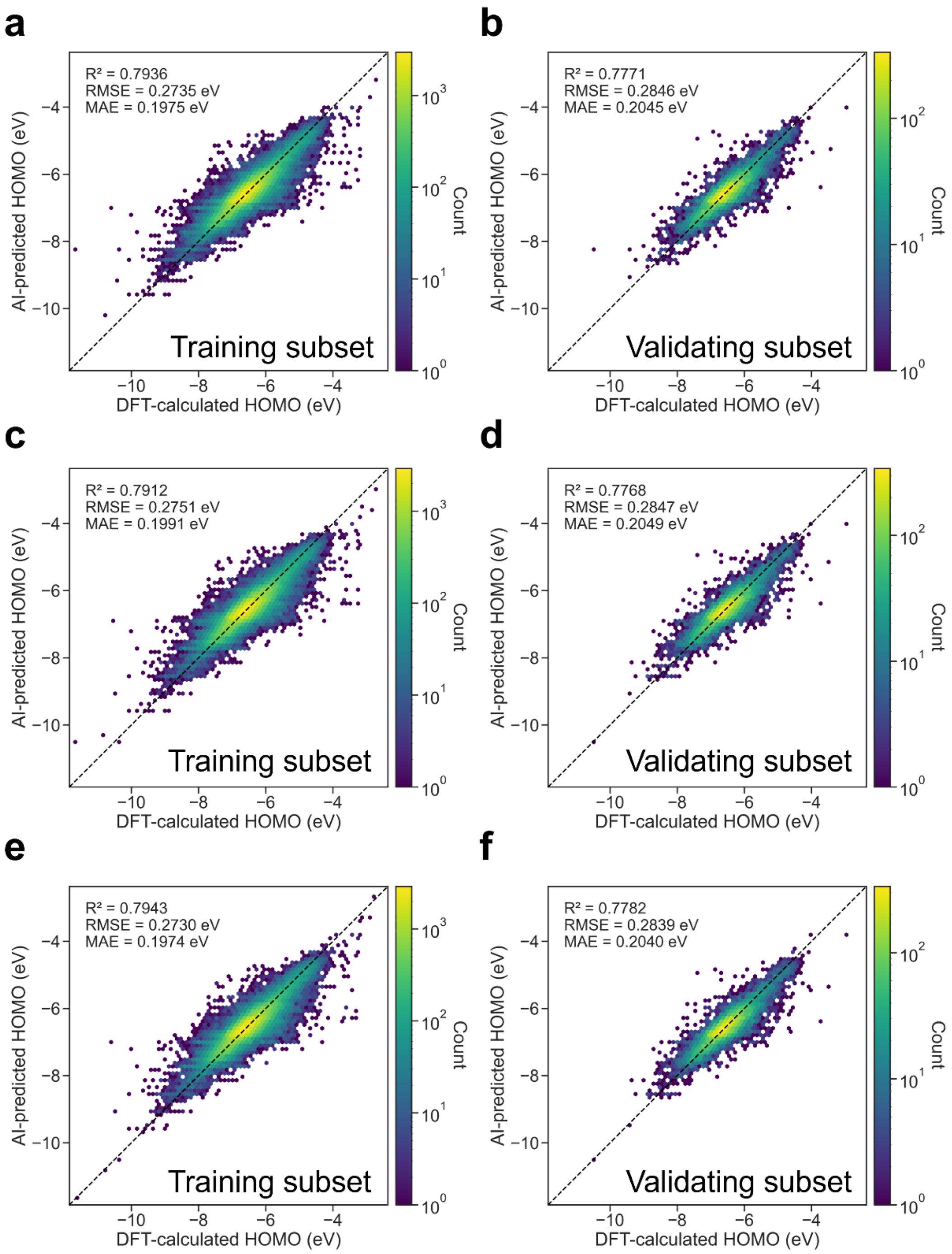


**Figure S13. HOMO Prediction Performance I of Boosting Models**

AI-predicted HOMO values of (a, b) LightGBM, (c, d) XGBoost, and (e, f) CatBoost models compared with corresponding DFT-calculated values for 133,018 organic molecules in (a, c, e) training and (b, d, f) validating subsets. *Input features:* RDKit-based fragment descriptors. The dashed line represents the ideal party ($y = x$). Model performance is quantified by three core parameters: $R^2$, RMSE, and MAE.

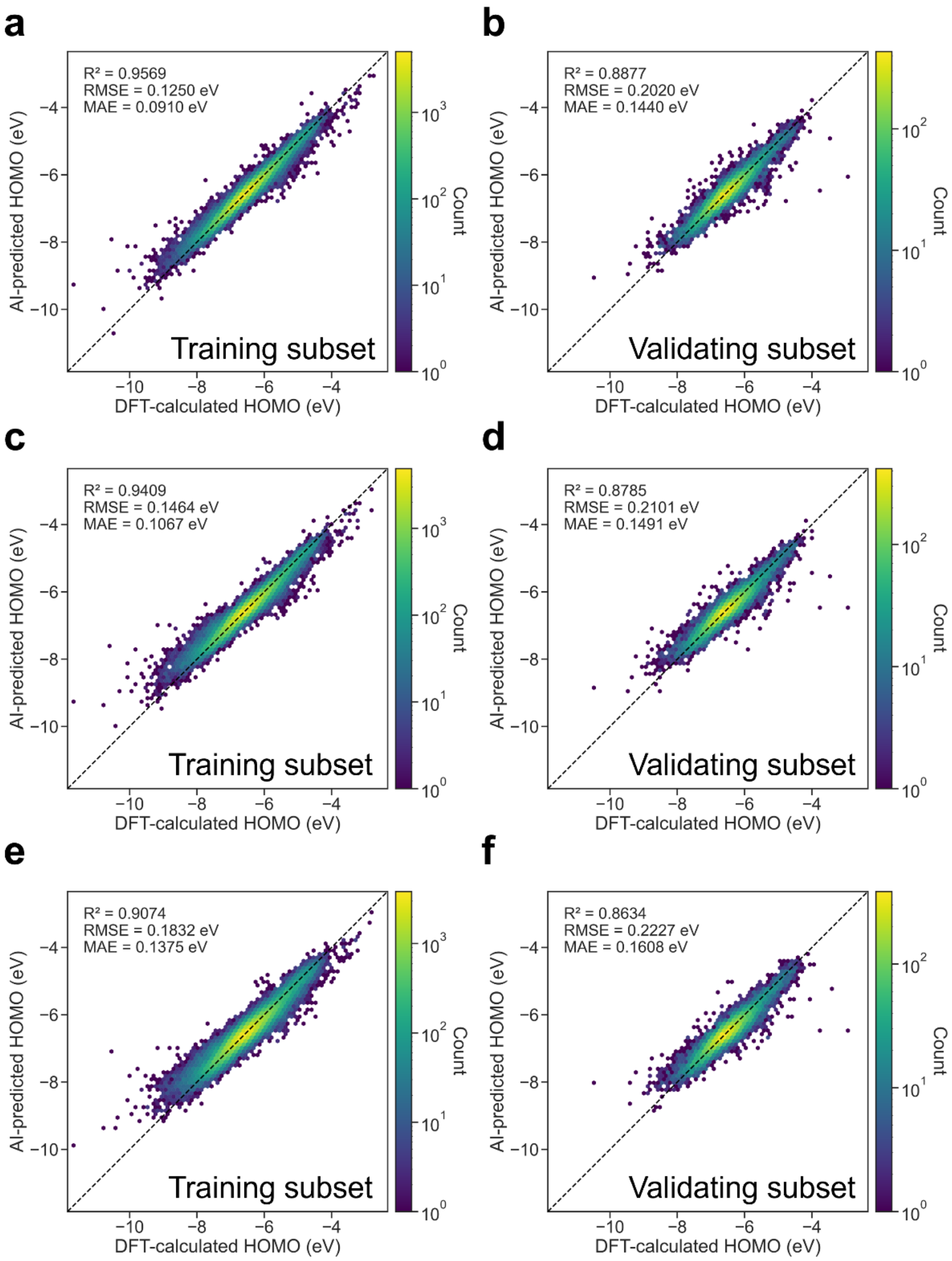


**Figure S14. HOMO Prediction Performance II of Boosting Models**

AI-predicted HOMO values of (a, b) LightGBM, (c, d) XGBoost, and (e, f) CatBoost models compared with corresponding DFT-calculated values for 133,018 organic molecules in (a, c, e) training and (b, d, f) validating subsets. *Input features:* Morgan fingerprints. The dashed line represents the ideal party ($y = x$). Model performance is quantified by three core parameters: $R^2$, RMSE, and MAE.

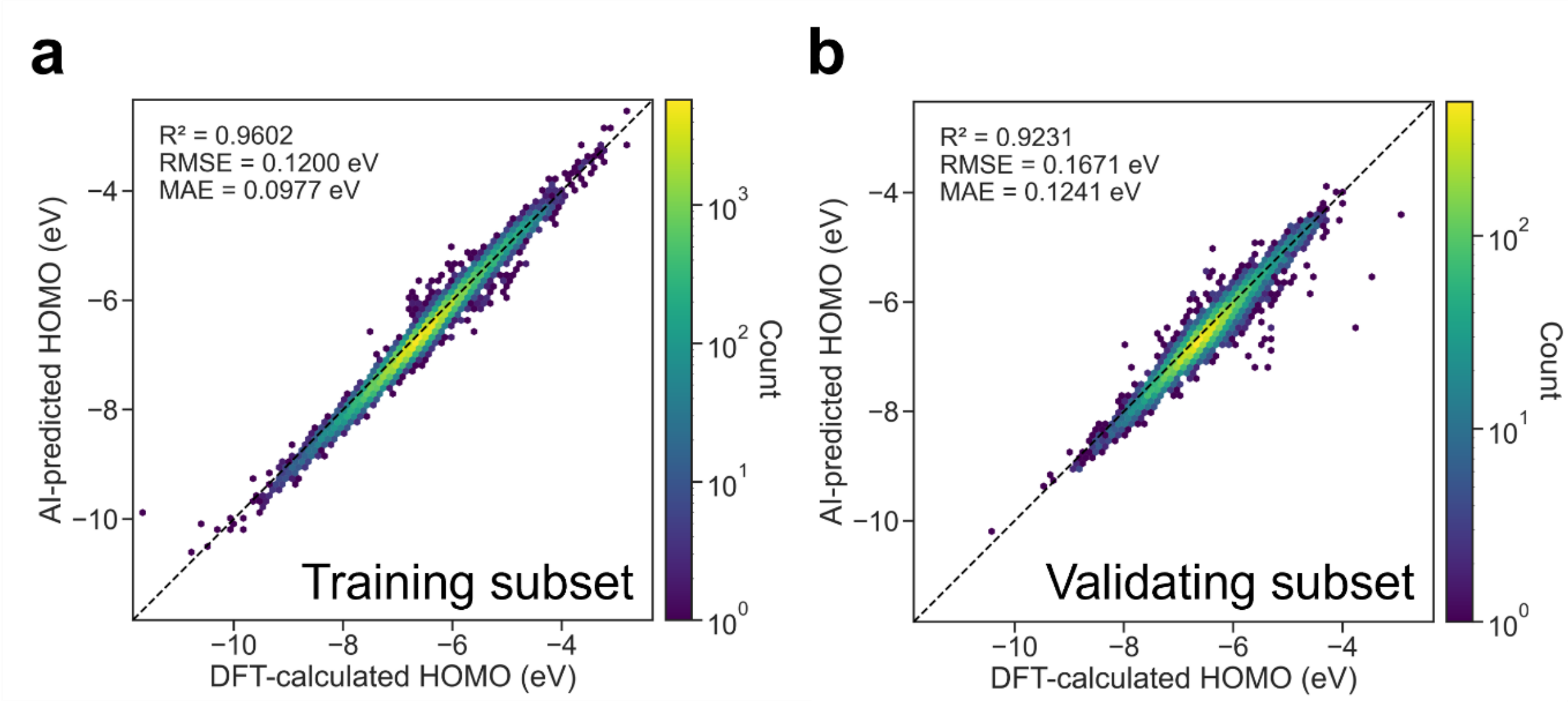


**Figure S15. HOMO Prediction Performance of ChemBERTa Model**

AI-predicted HOMO values of sequence-based ChemBERTa model, compared with corresponding DFT-calculated values for 133,018 organic molecules in (a) training and (b) validating subsets. The dashed line represents the ideal party ($y = x$). Model performance is quantified by three core parameters: $R^2$, RMSE, and MAE.

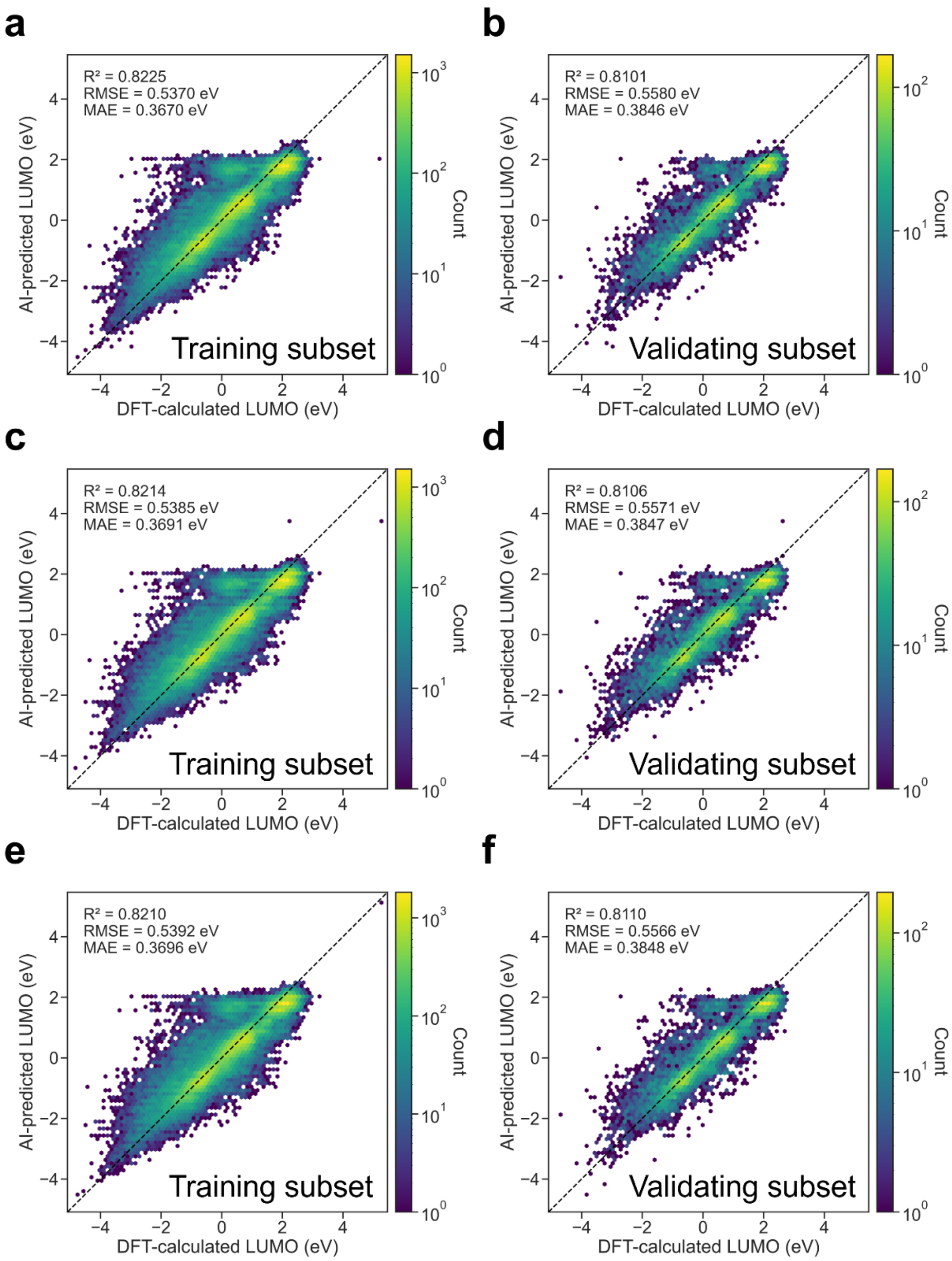


**Figure S16. LUMO Prediction Performance I of Boosting Models**

AI-predicted LUMO values of (a, b) LightGBM, (c, d) XGBoost, and (e, f) CatBoost models compared with corresponding DFT-calculated values for 133,018 organic molecules in (a, c, e) training and (b, d, f) validating subsets. *Input features:* RDKit-based fragment descriptors. The dashed line represents the ideal party ($y = x$). Model performance is quantified by three core parameters: $R^2$, RMSE, and MAE.

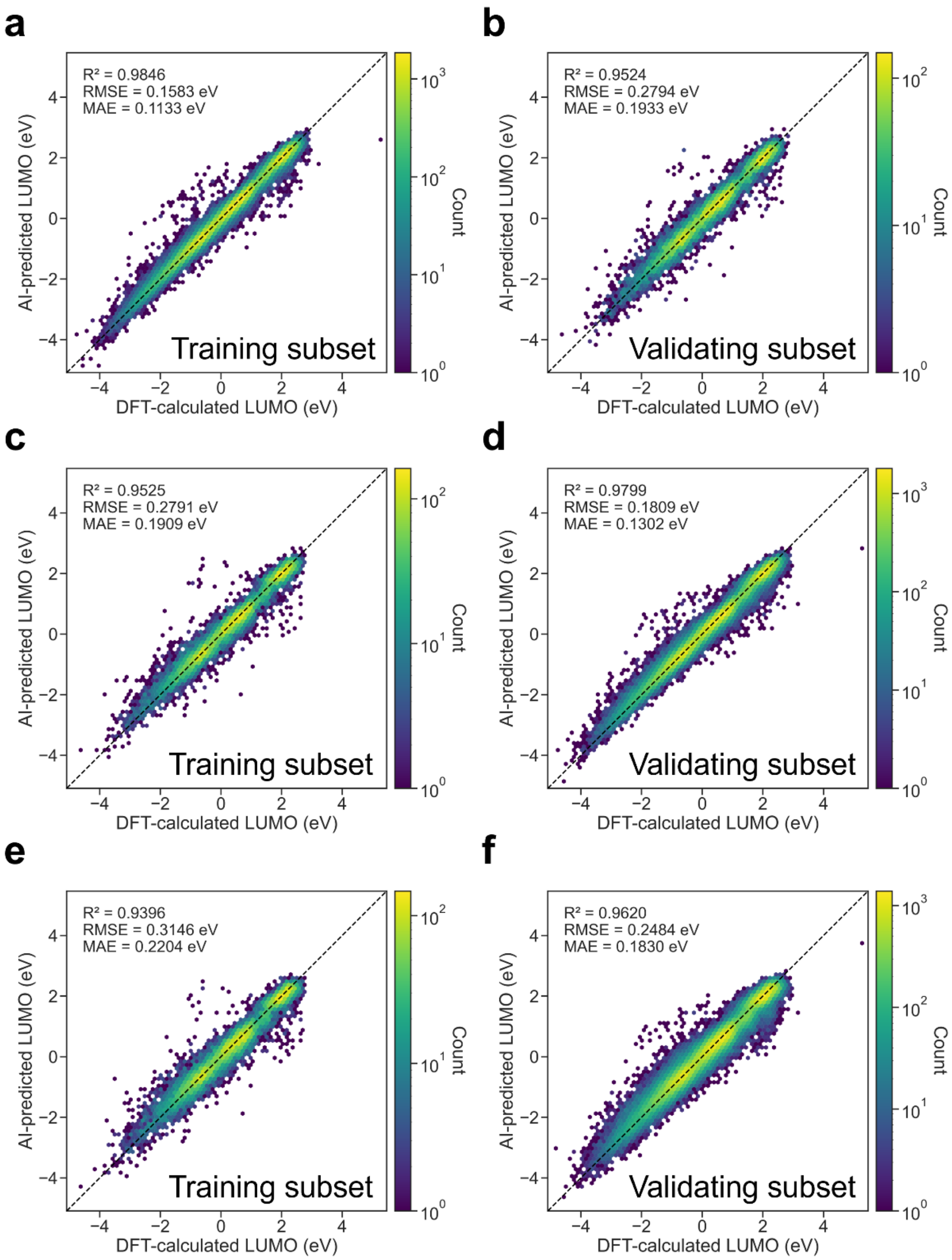


**Figure S17. LUMO Prediction Performance II of Boosting Models**

AI-predicted LUMO values of (a, b) LightGBM, (c, d) XGBoost, and (e, f) CatBoost models compared with corresponding DFT-calculated values for 133,018 organic molecules in (a, c, e) training and (b, d, f) validating subsets. *Input features:* Morgan fingerprints. The dashed line represents the ideal party ($y = x$). Model performance is quantified by three core parameters: $R^2$, RMSE, and MAE.

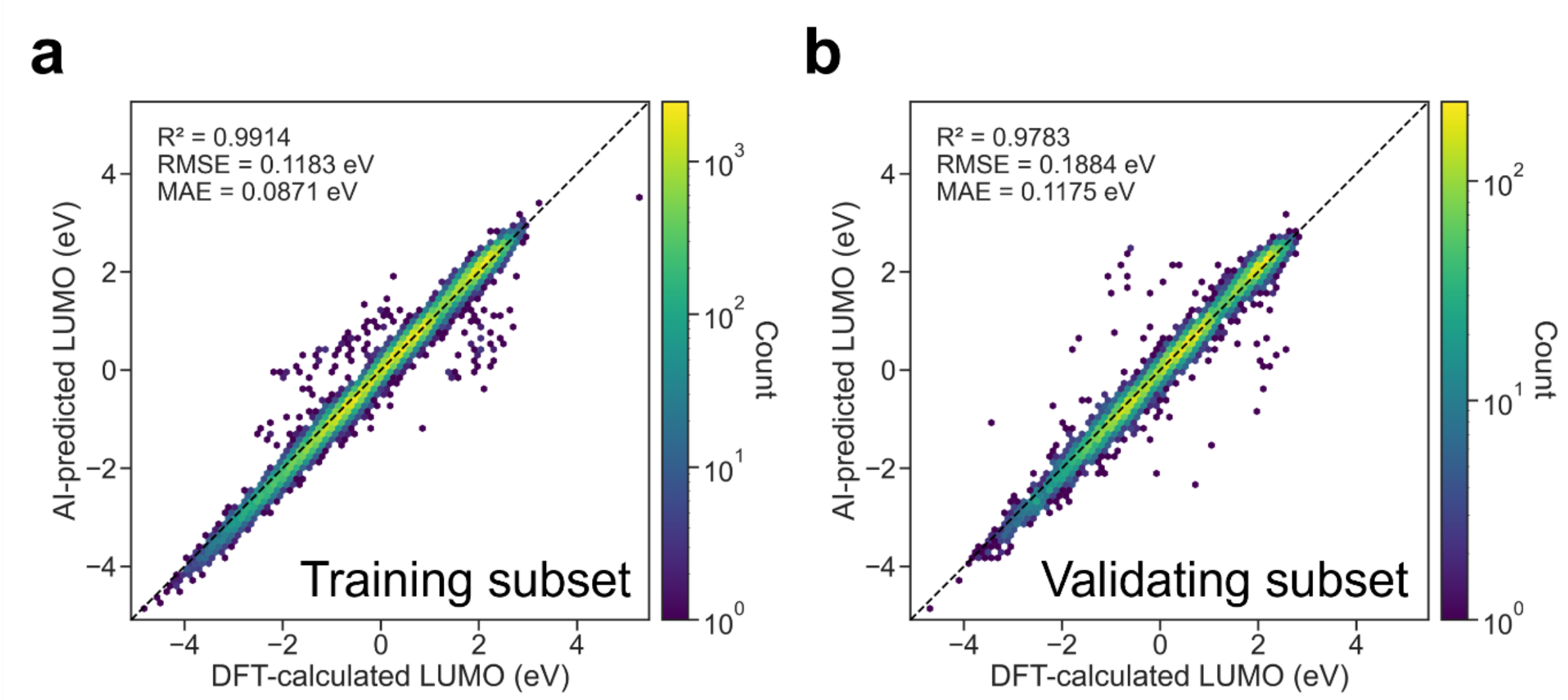


**Figure S18. LUMO Prediction Performance of ChemBERTa Model**

AI-predicted LUMO values of sequence-based ChemBERTa model, compared with corresponding DFT-calculated values for 133,018 organic molecules in (a) training and (b) validating subsets. The dashed line represents the ideal party ($y = x$). Model performance is quantified by three core parameters: $R^2$, RMSE, and MAE.

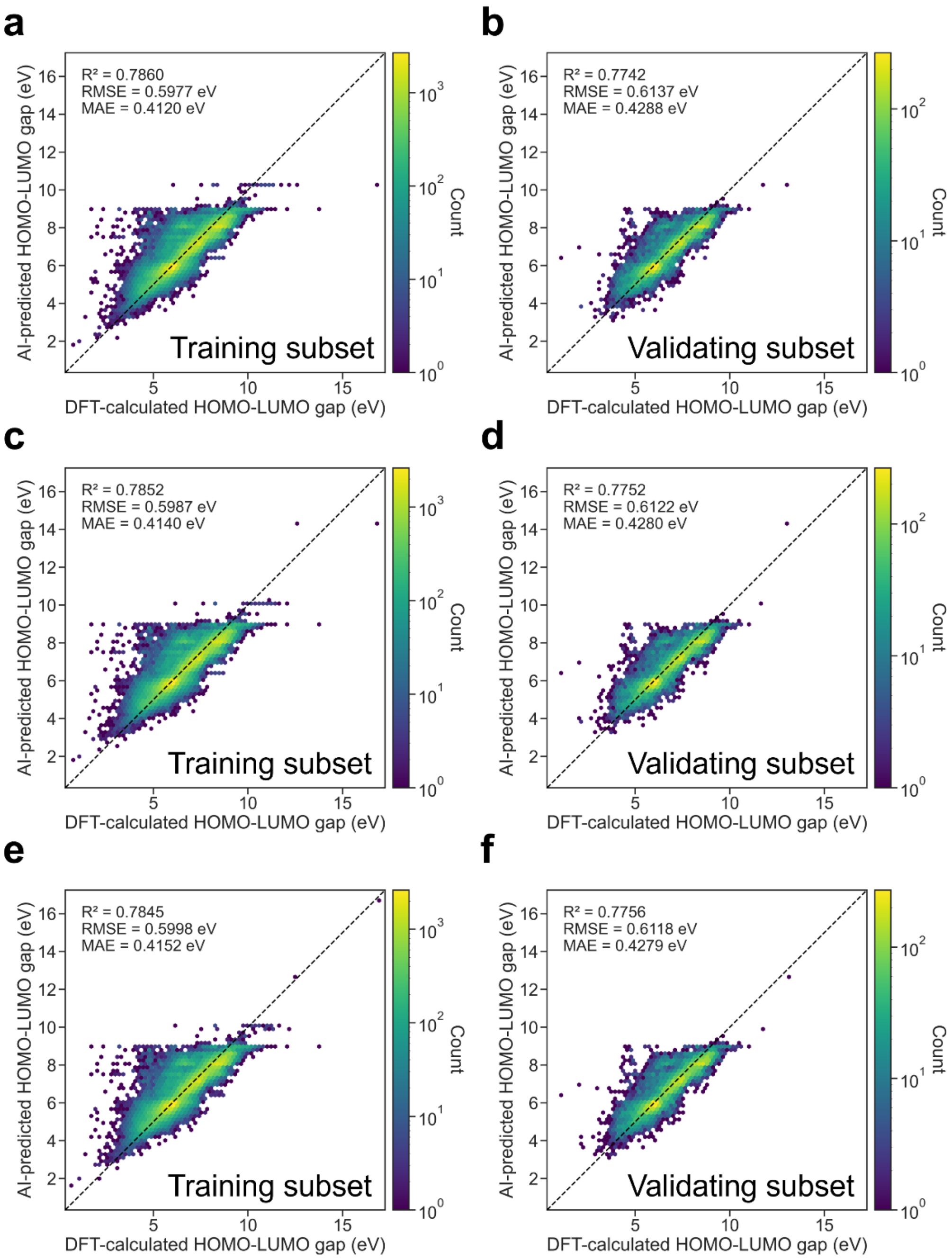


**Figure S19. HOMO–LUMO Gap Prediction Performance I of Boosting Models**

AI-predicted HOMO–LUMO gap values of (a, b) LightGBM, (c, d) XGBoost, and (e, f) CatBoost models compared with corresponding DFT-calculated values for 133,018 organic molecules in (a, c, e) training and (b, d, f) validating subsets. *Input features:* RDKit-based fragment descriptors. The dashed line represents the ideal party ($y = x$). Model performance is quantified by three core parameters: $R^2$, RMSE, and MAE.

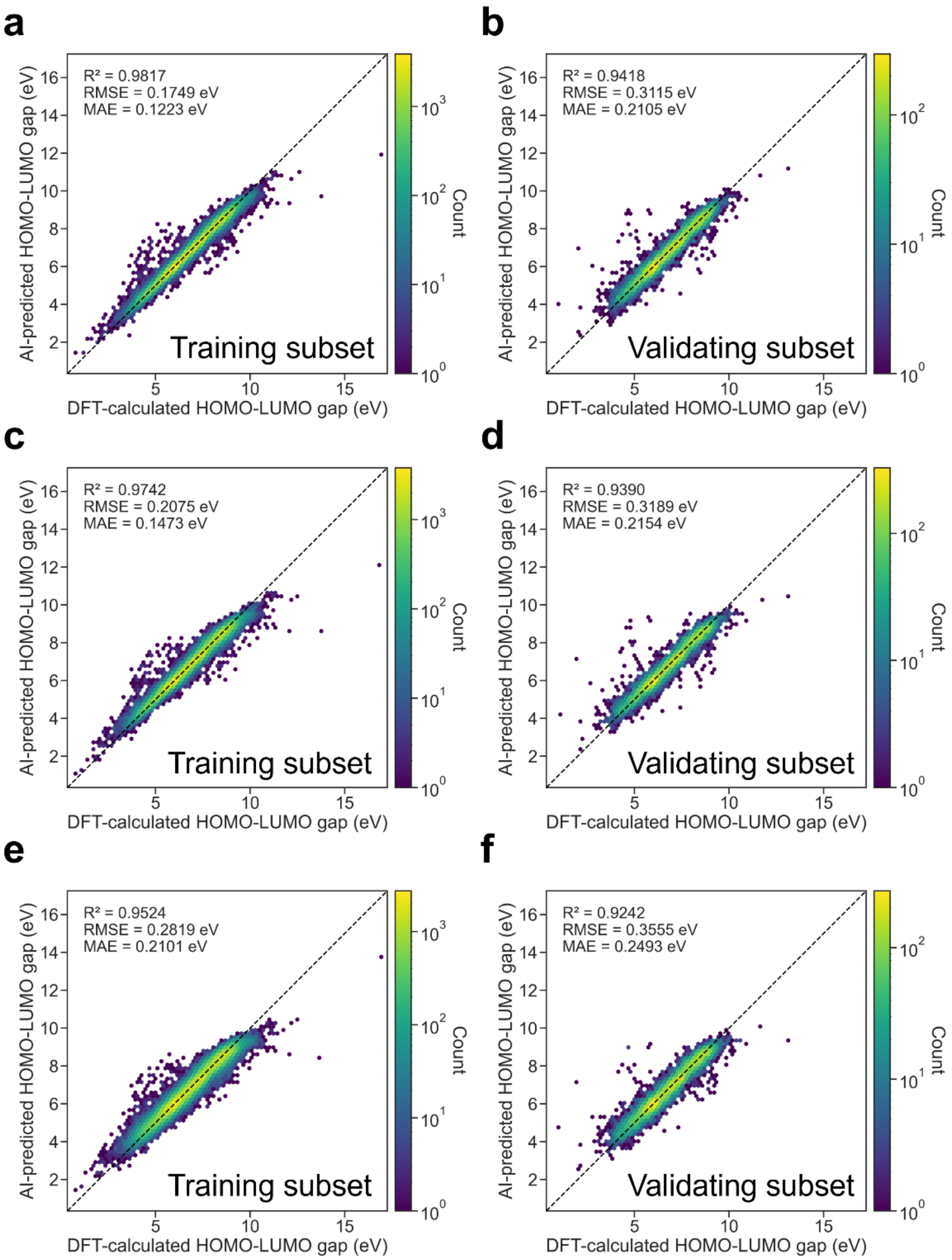


**Figure S20. HOMO–LUMO Gap Prediction Performance II of Boosting Models**

AI-predicted HOMO–LUMO gap values of (a, b) LightGBM, (c, d) XGBoost, and (e, f) CatBoost models compared with corresponding DFT-calculated values for 133,018 organic molecules in (a, c, e) training and (b, d, f) validating subsets. *Input features:* Morgan fingerprints. The dashed line represents the ideal party ($y = x$). Model performance is quantified by three core parameters: $R^2$, RMSE, and MAE.

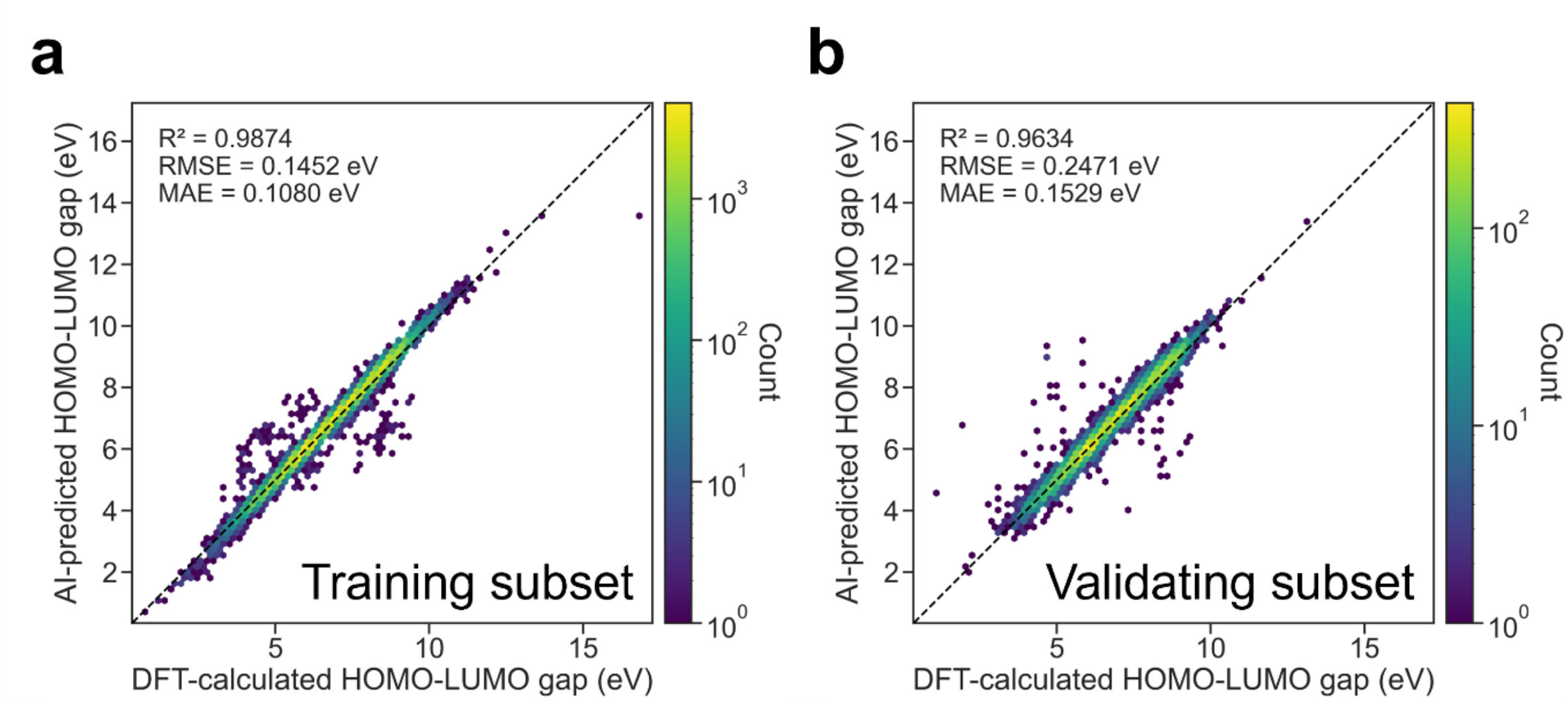


**Figure S21. HOMO–LUMO Gap Prediction Performance of ChemBERTa Model**

AI-predicted HOMO–LUMO gap values of sequence-based ChemBERTa model, compared with corresponding DFT-calculated values for 133,018 organic molecules in (a) training and (b) validating subsets. The dashed line represents the ideal party ($y = x$). Model performance is quantified by three evaluation parameters: $R^2$, RMSE, and MAE.

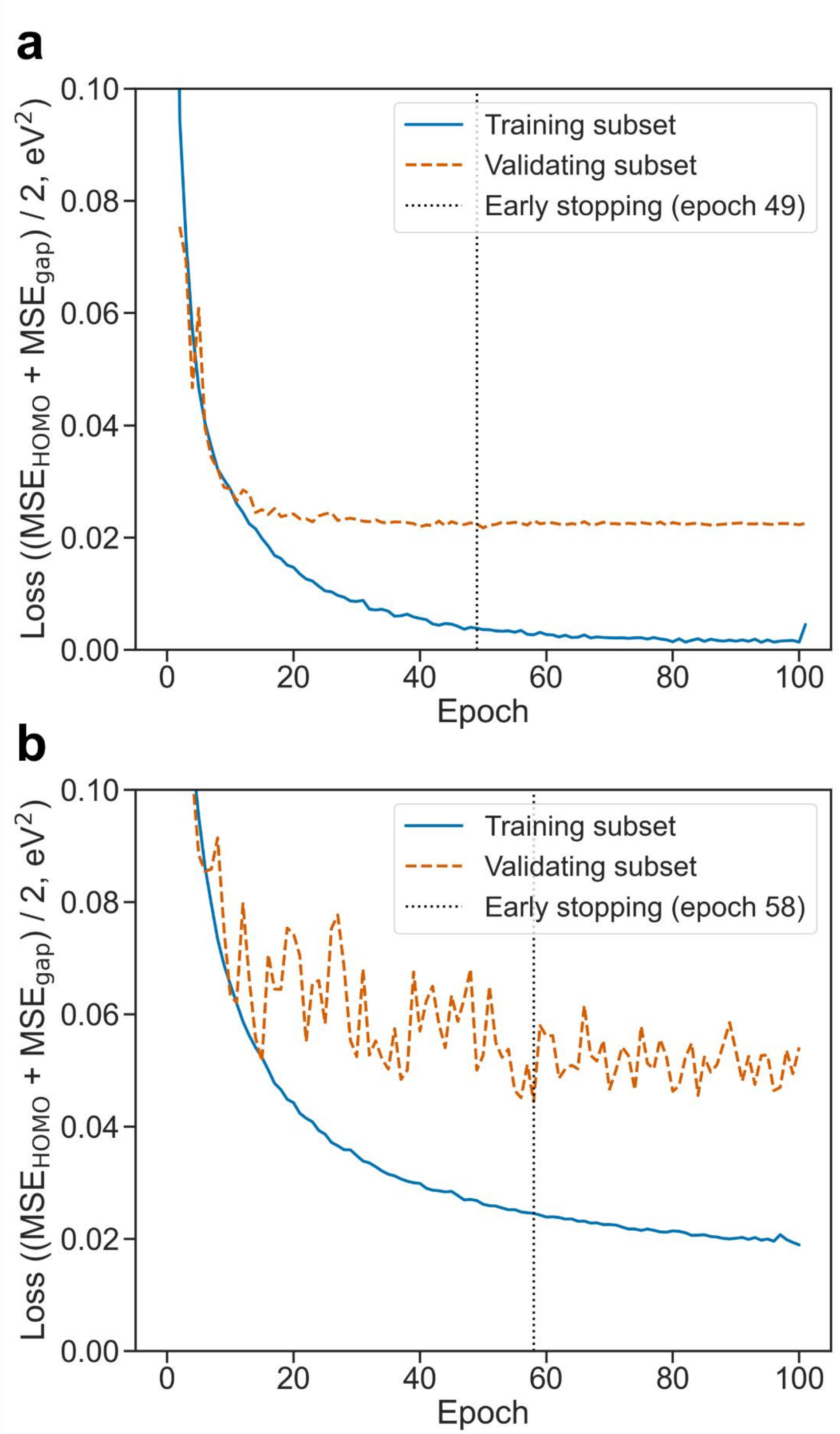


**Figure S22. Learning Curves of DMPNN and ChemBERTa Models**

Change in mean squared error tracked during learning process for each of (a) DMPNN, (b) ChemBERTa-finetuned, and (c) ChemBERTa-pretrained models.

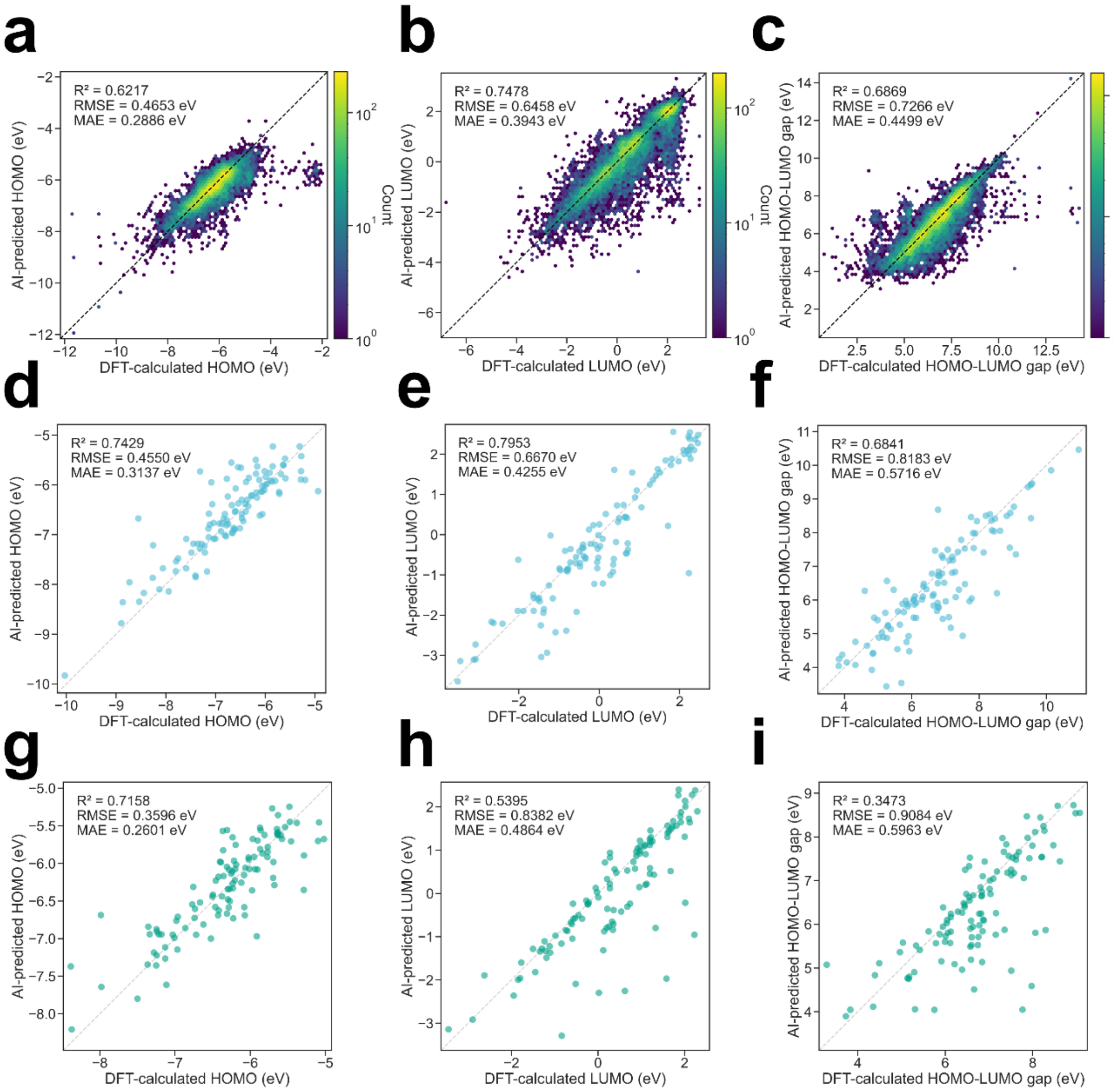


**Figure S23. Transferability of QM9-Trained DMPNN Model to External Datasets**

Comparisons between DMPNN-predicted and dataset-native DFT-calculated values of (a) HOMO energy, (b) LUMO energy, and (c) HOMO–LUMO gap for PubChemQC dataset. (d-f) Corresponding comparisons for 110 molecules selected from OE62 dataset and recalculated at the B3LYP/6-31G(2df,p) level. (g-i) Corresponding comparisons for 110 molecules selected from QMugs dataset and recalculated at the B3LYP/6-31G(2df,p) level. The dashed lines indicate perfect agreement between the AI predictions and DFT calculations. In (a-c), Color indicates the number of data overlapping. Evaluation metrics, including $R^2$, RMSE, and MAE are shown in each panel.

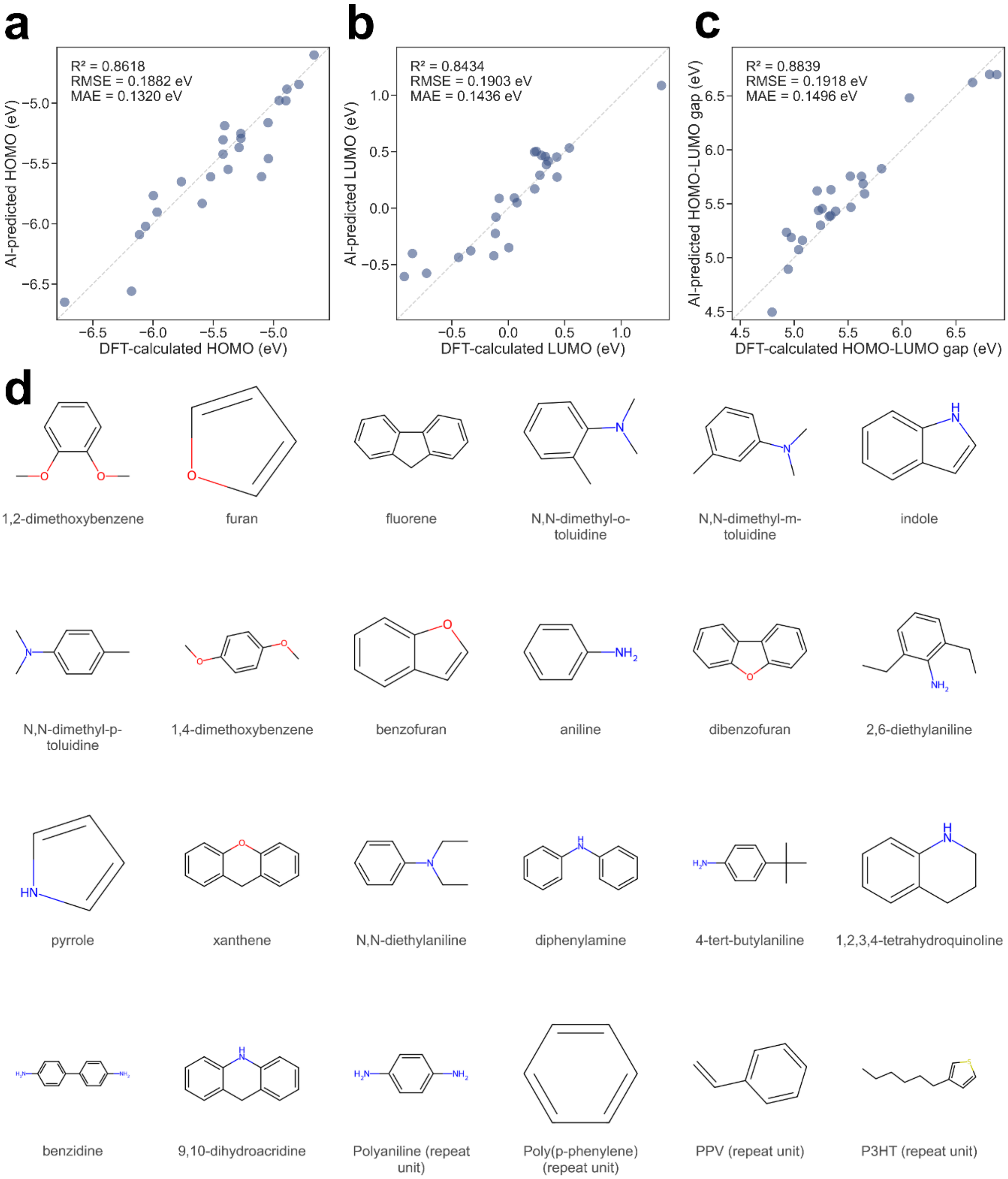


**Figure S24. Transferability of QM9-Trained DMPNN Model to Experimental Donors**

Comparisons between DMPNN-predicted and DFT (B3LYP/6-31G(2df,p) level)-calculated values of (a) HOMO energy, (b) LUMO energy, and (c) HOMO–LUMO gap for (d) 24 experimentally reported organic donor units. The dashed lines indicate perfect agreement between the AI predictions and DFT calculations. Evaluation metrics, including $R^2$, RMSE, and MAE are shown in each panel.

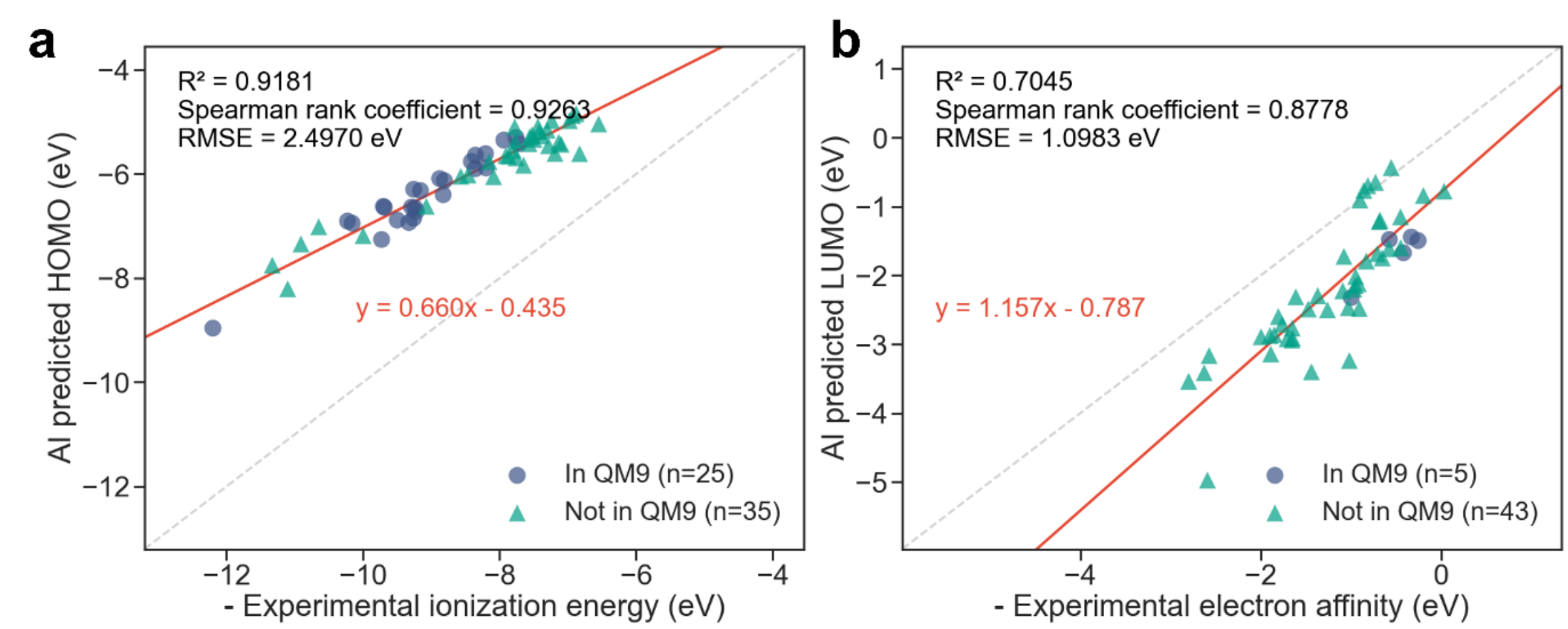


**Figure S25. IE/EA-Based Experimental Consistency of AI-Predicted Frontier Orbital Energies**

Comparisons of (a) AI-predicted HOMO energies with negative experimental IEs and AI-predicted LUMO energies with the negative experimental EAs. Data points are categorized according to whether the corresponding molecule is included in the QM9 dataset. The solid lines represent linear regression fits with trend equations, while the dashed lines indicate ideal parity ($y = x$). Evaluation metrics, including $R^2$, Spearman rank coefficient ρ, and RMSE, are shown in each panel.

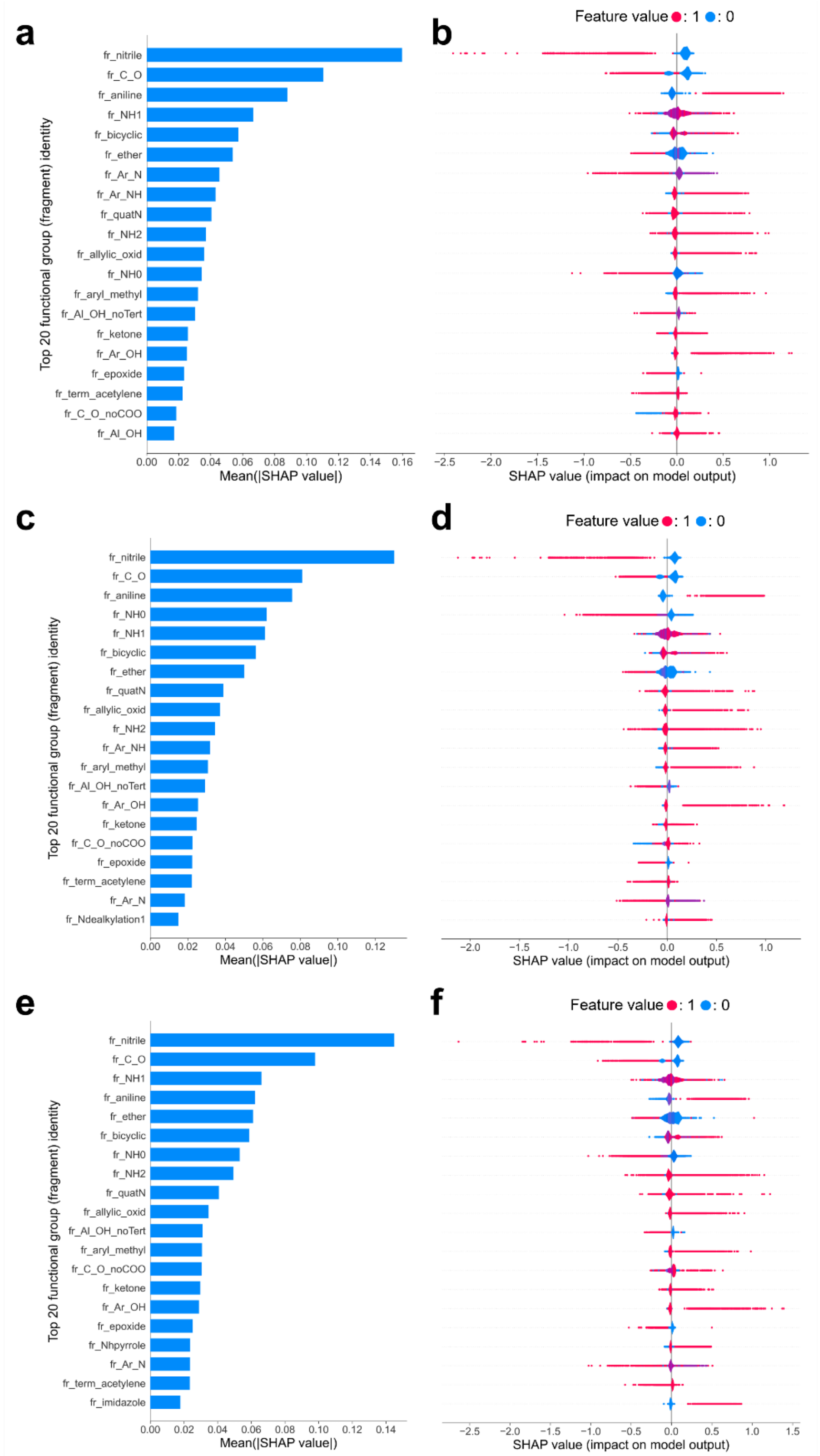


**Figure S26. Interpretability Analyses of Boosting Models: HOMO**

Mean absolute SHAP values of the top 20 functional group (fragment) descriptors contributing to HOMO prediction in QM9 dataset for (a) LightGBM, (c) XGBoost, and (e) CatBoost models, along with the corresponding SHAP value distributions for individual molecules in (b), (d), and (f), respectively.

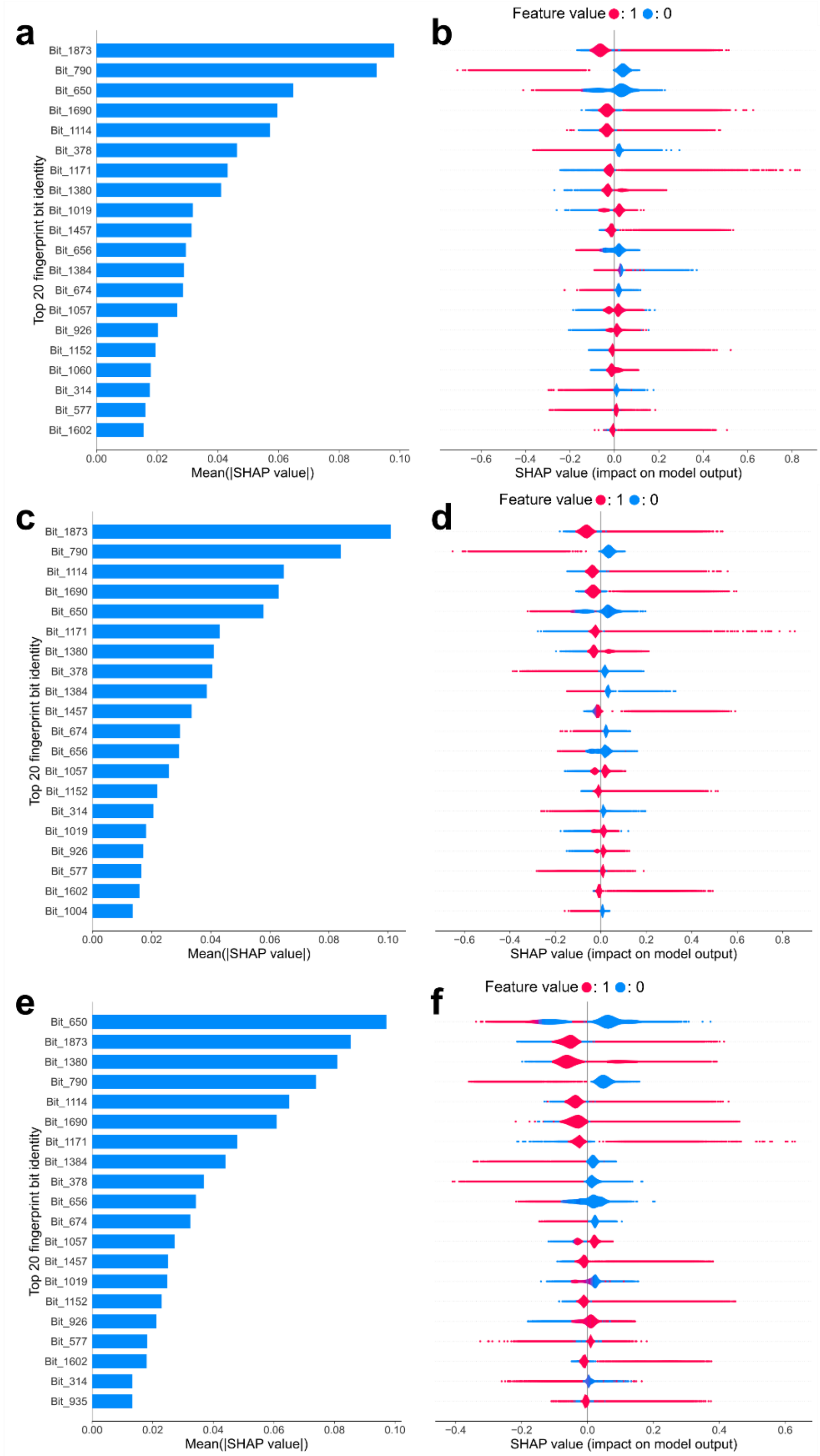


**Figure S27. Interpretability Analyses of Boosting Models: HOMO**

Mean absolute SHAP values of the top 20 Morgan fingerprint-based bit IDs contributing to HOMO prediction in QM9 dataset for (a) LightGBM, (c) XGBoost, and (e) CatBoost models, along with the corresponding SHAP value distributions for individual molecules in (b), (d), and (f), respectively.

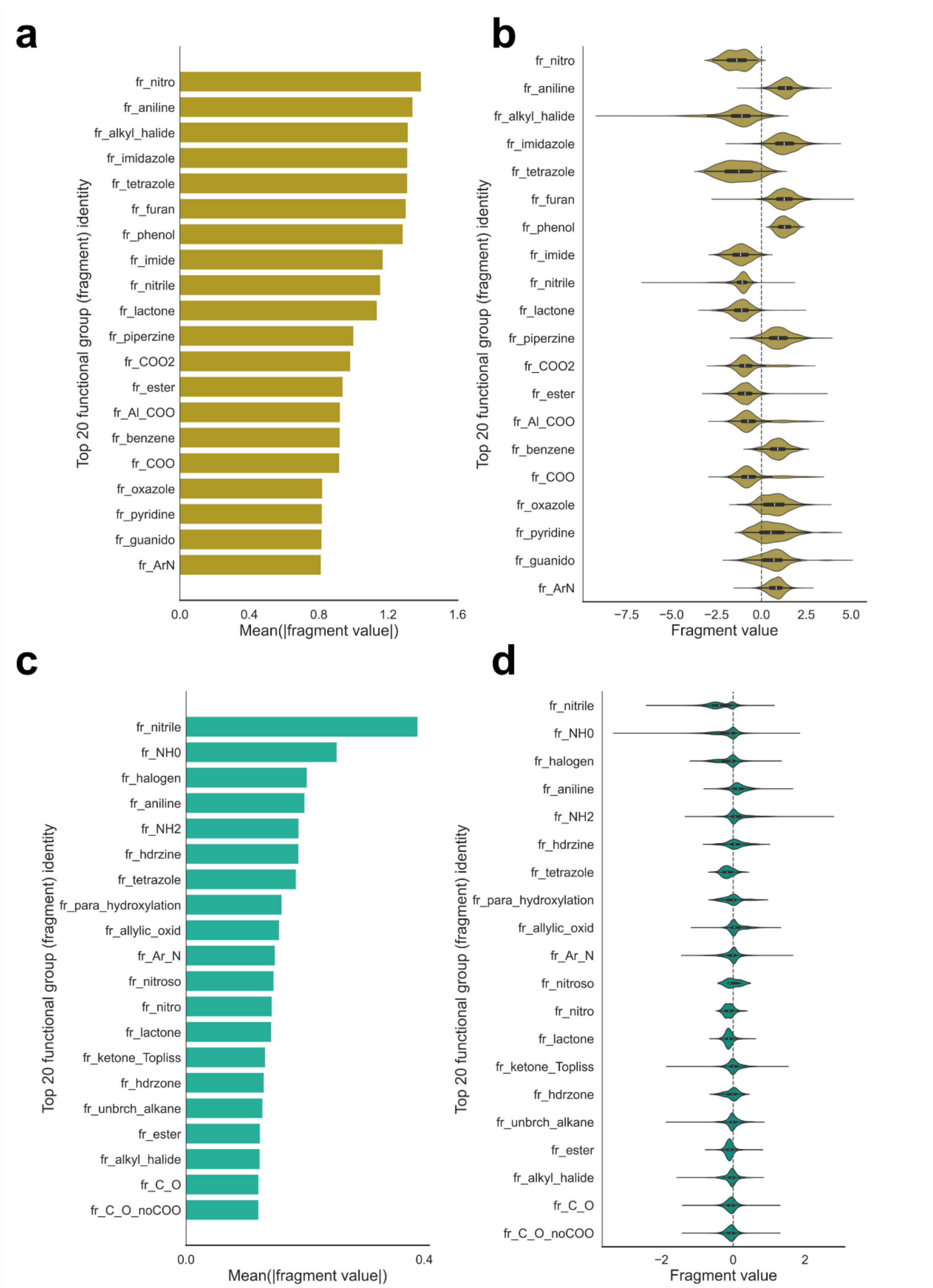


**Figure S28. Interpretability Analyses of Deep Learning Models: HOMO**

Mean absolute fragment values of the top 20 functional group (fragment) descriptors contributing to HOMO prediction in QM9 dataset for (a) DMPNN and (c) ChemBERTa models, along with the corresponding fragment value distributions for individual molecules in (b) and (d), respectively.

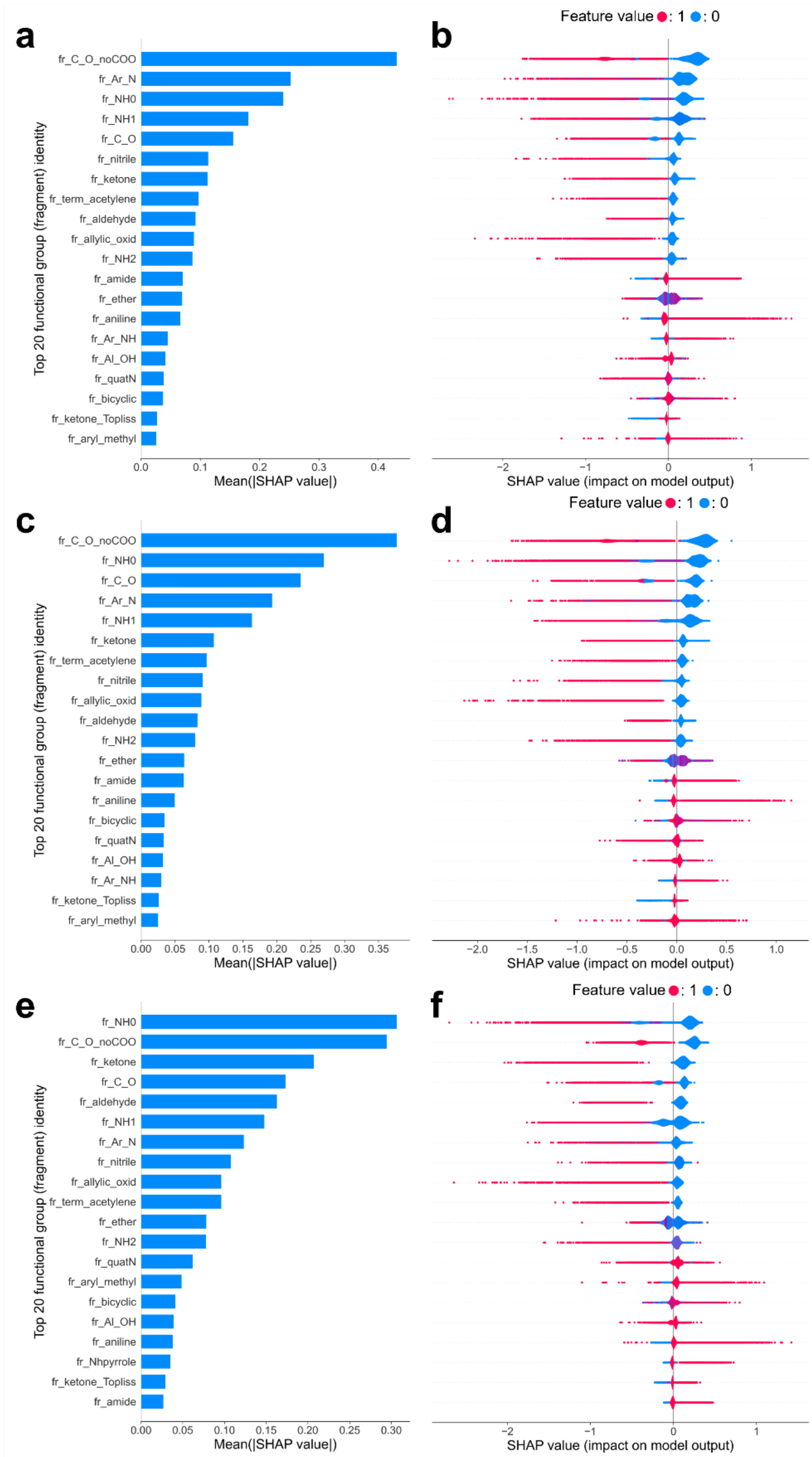


**Figure S29. Interpretability Analyses of Boosting Models: LUMO**

Mean absolute SHAP values of the top 20 functional group (fragment) descriptors contributing to LUMO prediction in QM9 dataset for (a) LightGBM, (c) XGBoost, and (e) CatBoost models, along with the corresponding SHAP value distributions for individual molecules in (b), (d), and (f), respectively.

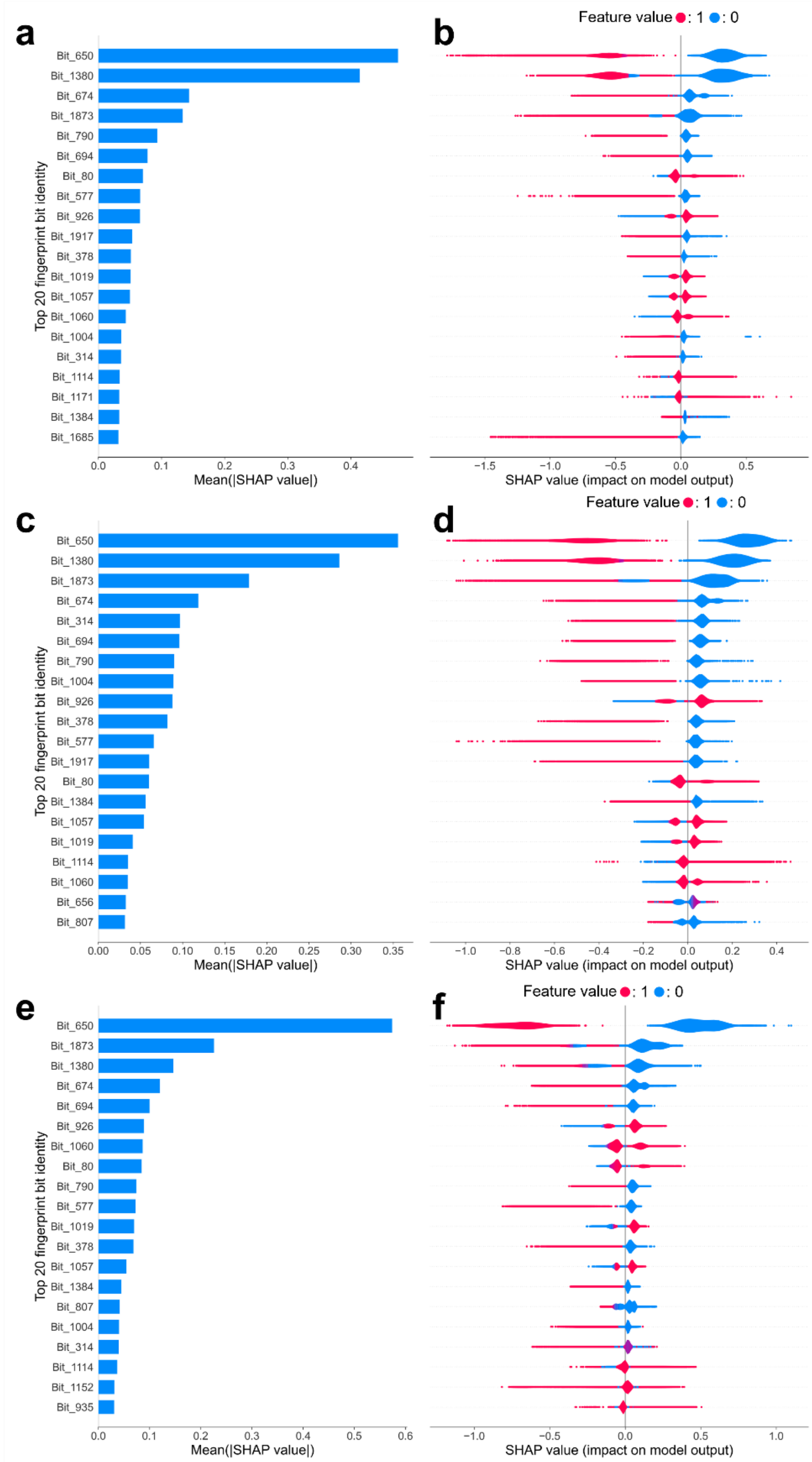


**Figure S30. Interpretability Analyses of Boosting Models: LUMO**

Mean absolute SHAP values of the top 20 Morgan fingerprint-based bit IDs contributing to LUMO prediction in QM9 dataset for (a) LightGBM, (c) XGBoost, and (e) CatBoost models, along with the corresponding SHAP value distributions for individual molecules in (b), (d), and (f), respectively.

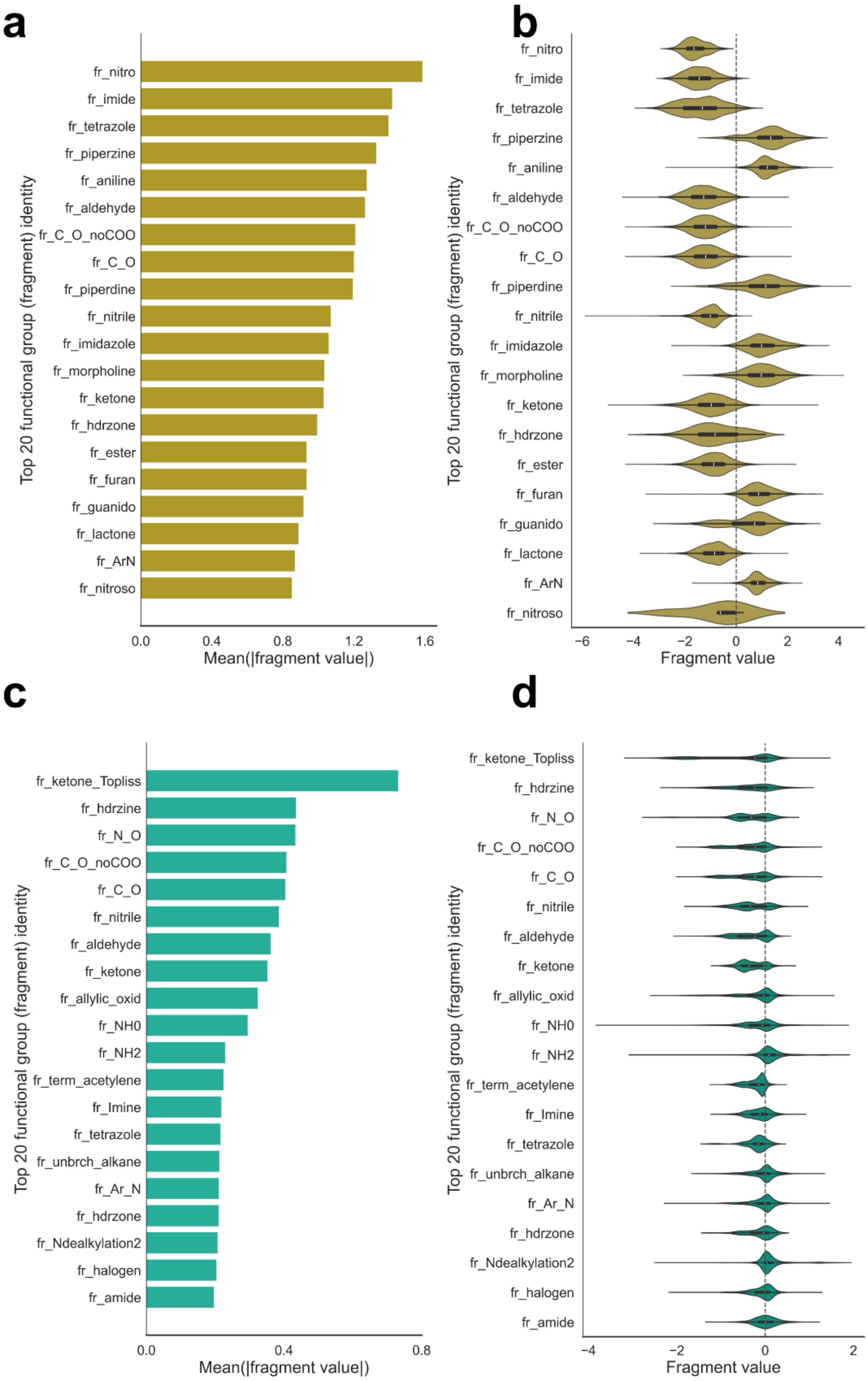


**Figure S31. Interpretability Analyses of Deep Learning Models: LUMO**

Mean absolute fragment values of the top 20 functional group (fragment) descriptors contributing to LUMO prediction in QM9 dataset for (a) DMPNN and (c) ChemBERTa models, along with the corresponding fragment value distributions for individual molecules in (b) and (d), respectively.

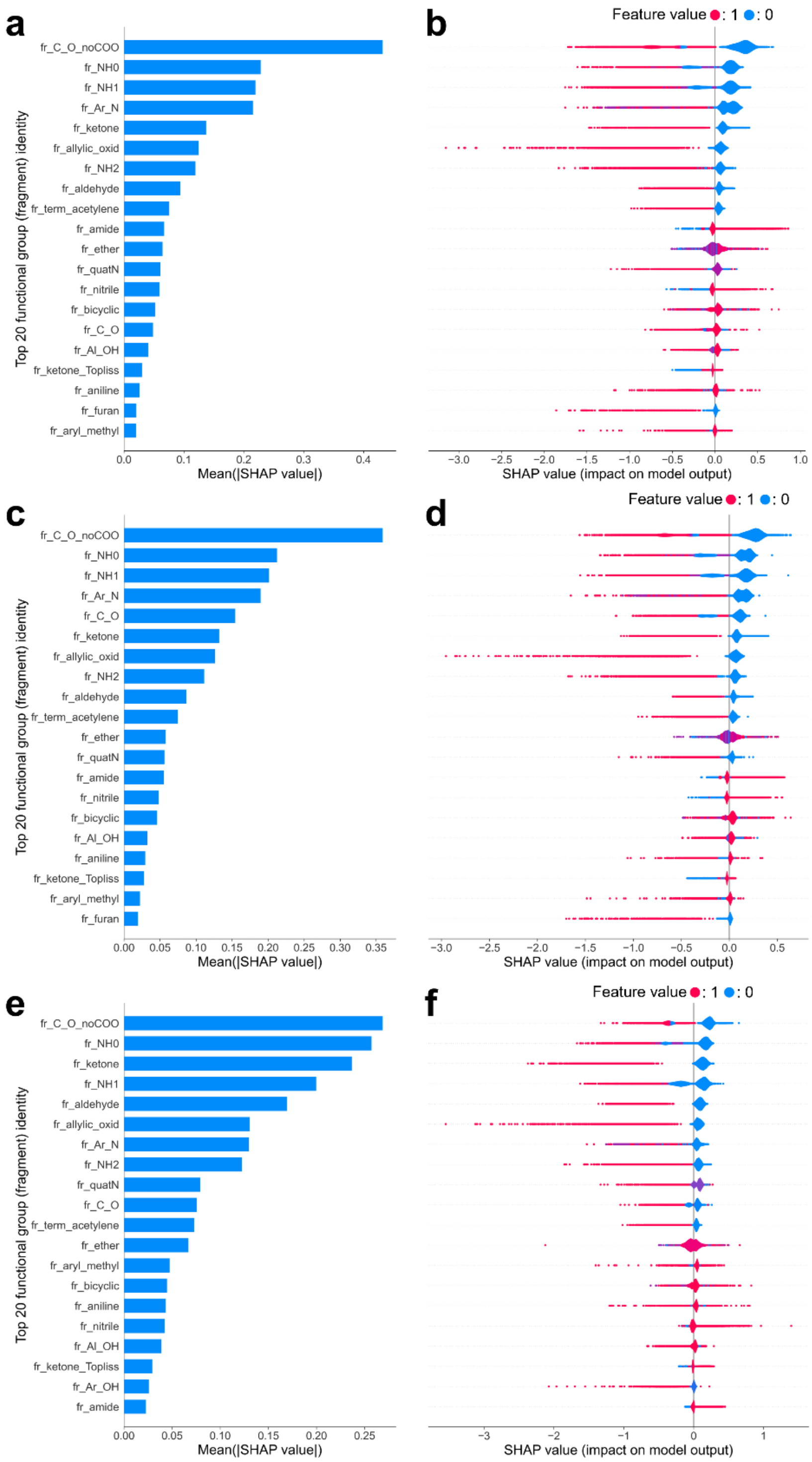


**Figure S32. Interpretability Analyses of Boosting Models: HOMO–LUMO Gap**

Mean absolute SHAP values of the top 20 functional group (fragment) descriptors contributing to HOMO–LUMO gap prediction in QM9 dataset for (a) LightGBM, (c) XGBoost, and (e) CatBoost models, along with the corresponding SHAP value distributions for individual molecules in (b), (d), and (f), respectively.

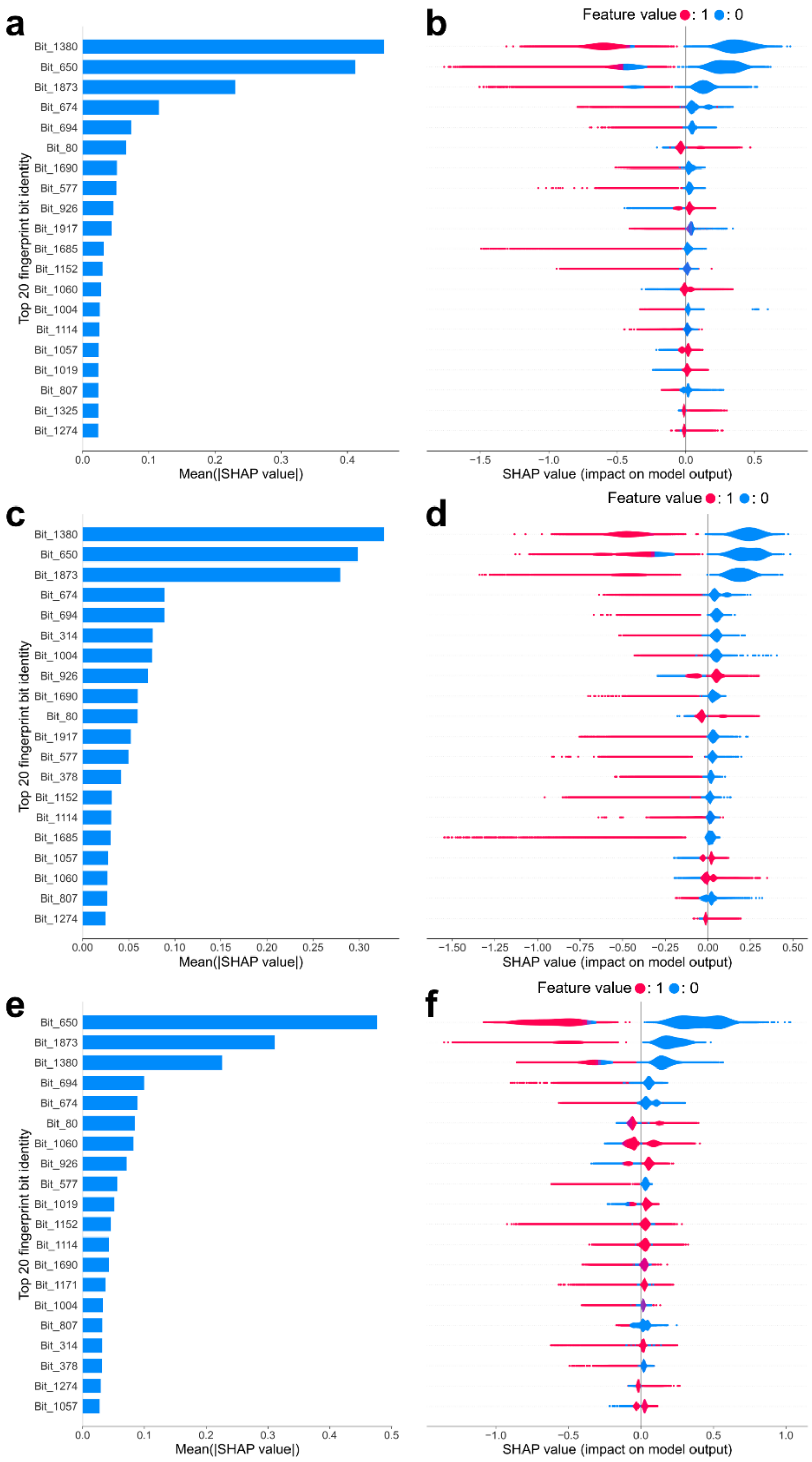


**Figure S33. Interpretability Analyses of Boosting Models: HOMO–LUMO Gap**

Mean absolute SHAP values of the top 20 Morgan fingerprint-based bit IDs contributing to HOMO–LUMO gap prediction in QM9 dataset for (a) LightGBM, (c) XGBoost, and (e) CatBoost models, along with the corresponding SHAP value distributions for individual molecules in (b), (d), and (f), respectively.

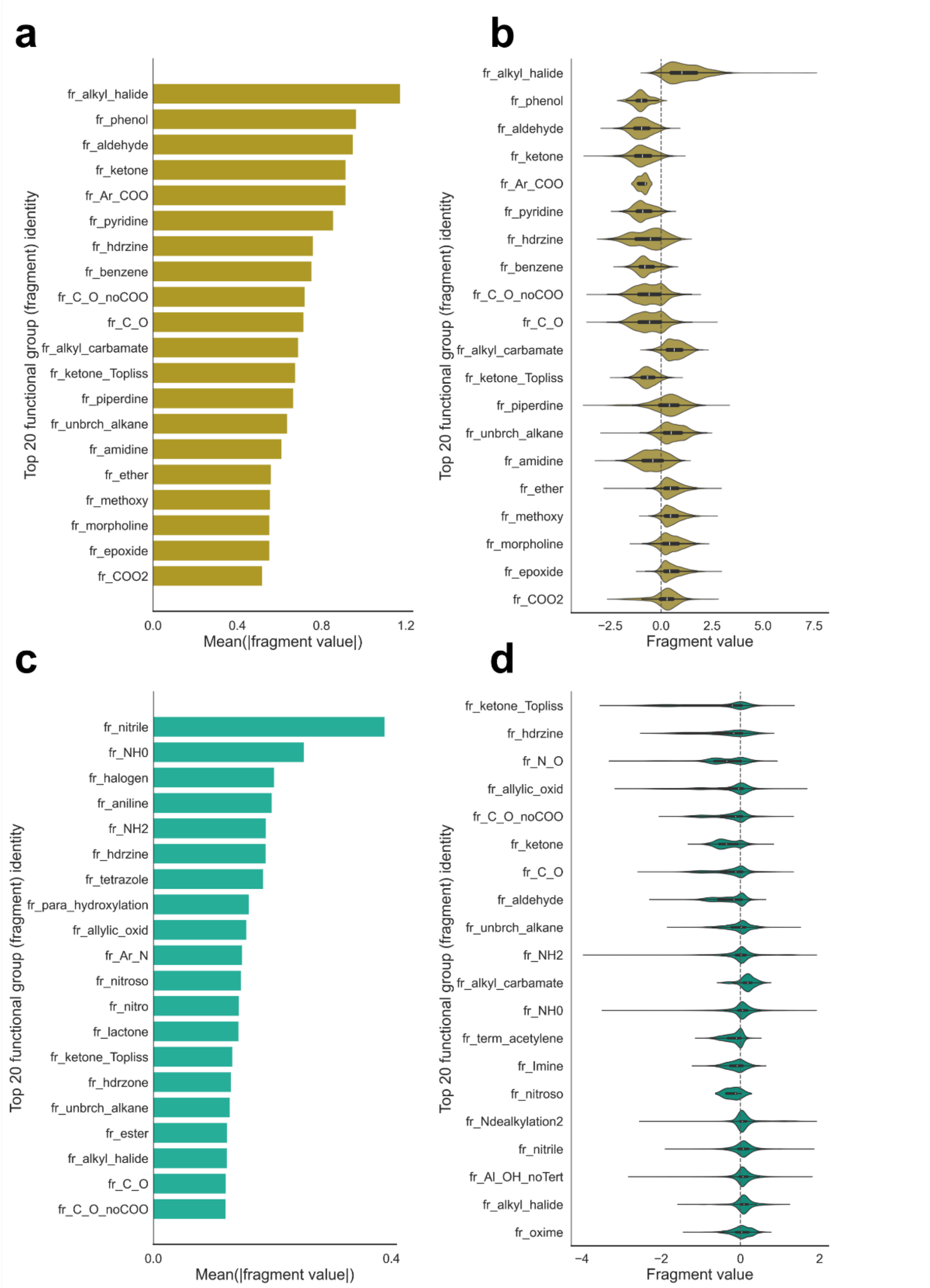


**Figure S34. Interpretability Analyses of Deep Learning Models: HOMO–LUMO Gap** Mean absolute fragment values of the top 20 functional group (fragment) descriptors contributing to HOMO–LUMO gap prediction in QM9 dataset for (a) DMPNN and (c) ChemBERTa models, along with the corresponding fragment value distributions for individual molecules in (b) and (d), respectively.

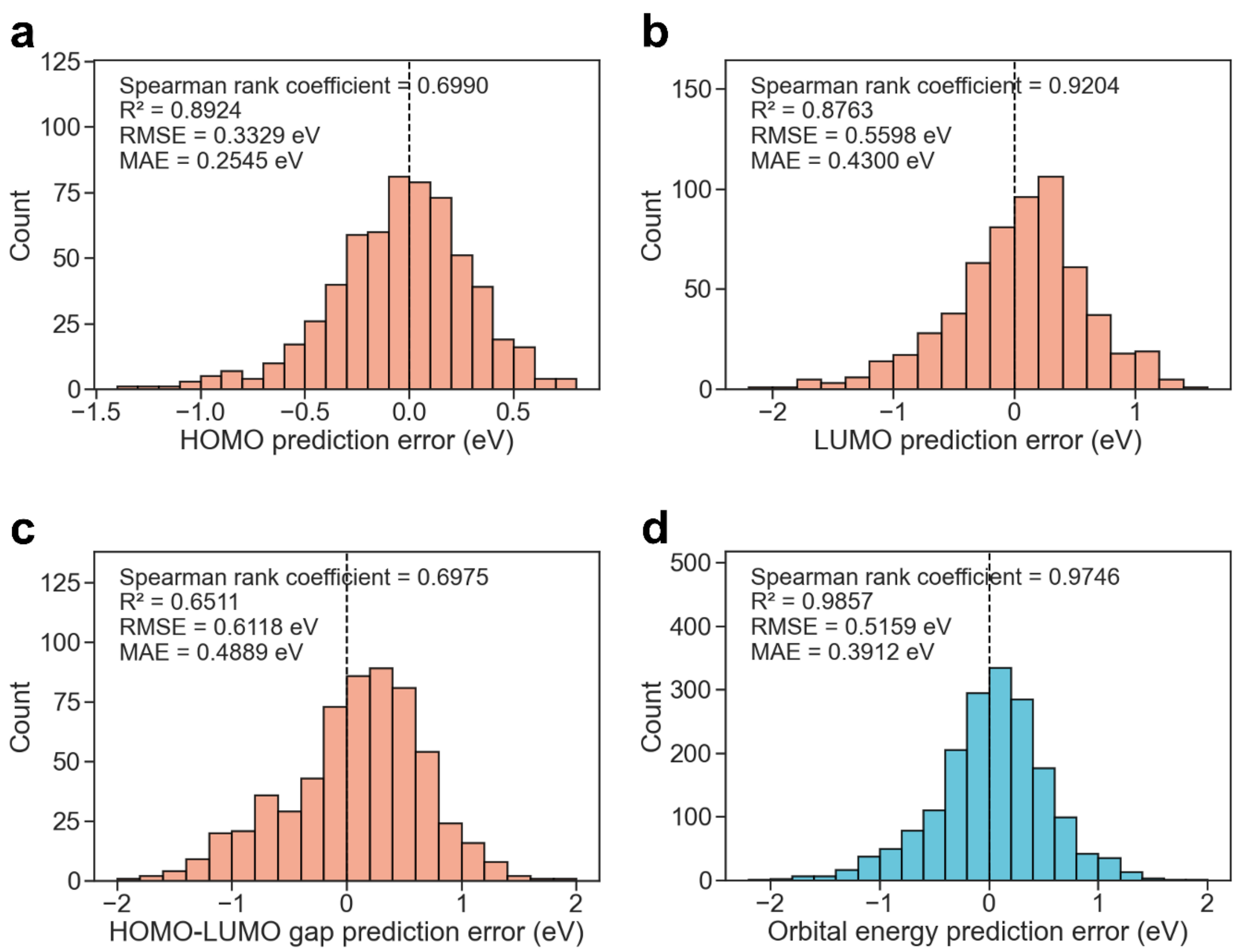


**Figure S35. Distributions of AI Prediction Errors Relative to DFT**

Histograms of AI prediction errors for (a) HOMO energy, (b) LUMO energy, and (c) HOMO–LUMO gap, defined as the difference between the AI-predicted and corresponding DFT-calculated values. (d) Aggregated distribution of the AI prediction errors across the three frontier-orbital properties. The vertical dashed line indicates zero prediction error. Evaluation metrics, including $R^2$, Spearman rank coefficient ρ, RMSE, and MAE are shown in each panel.

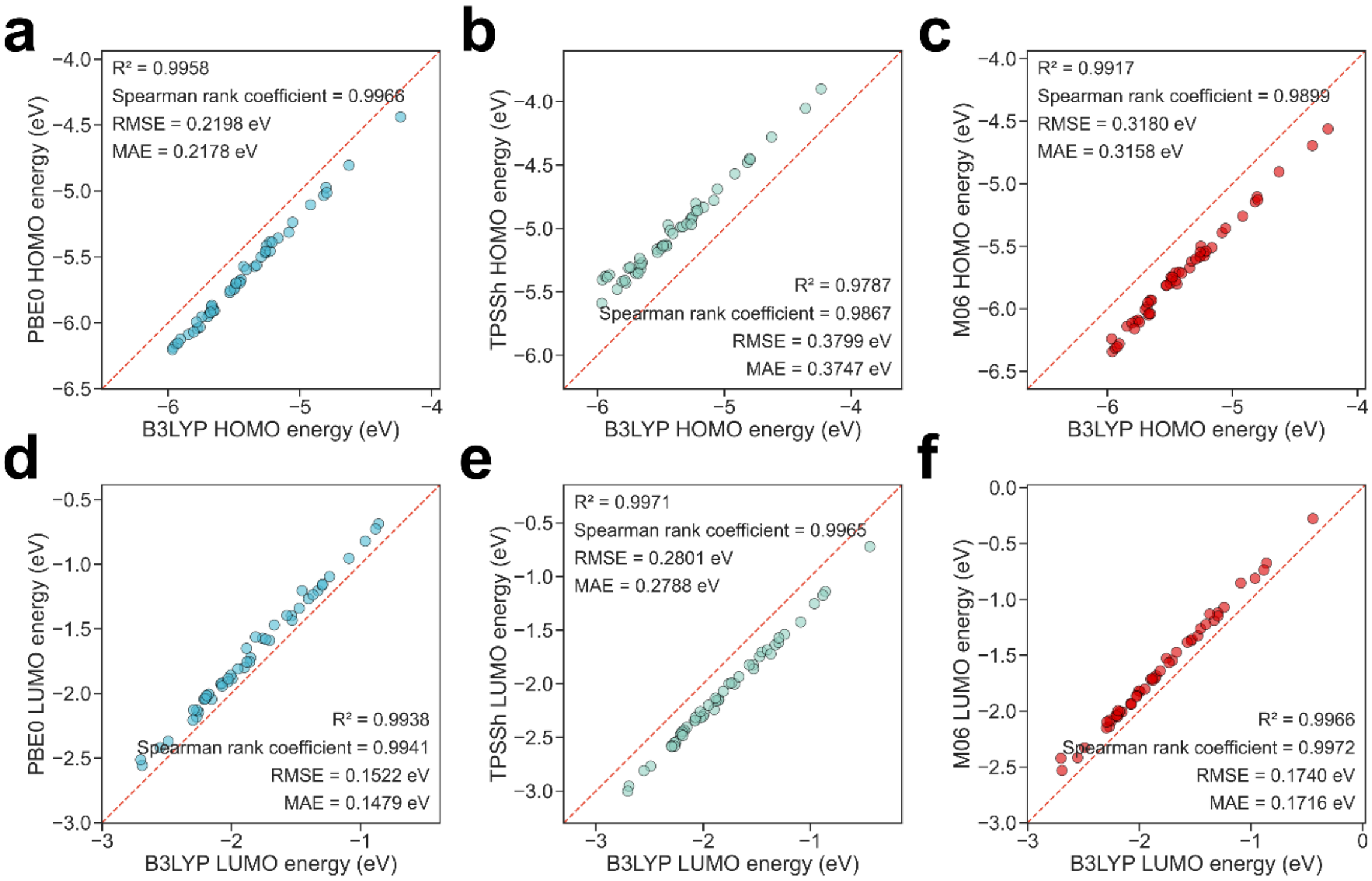


**Figure S36. DFT Functional Dependence Analysis of 50 Screened Candidates**

Comparisons of B3LYP-calculated frontier orbital energies with those calculated using three additional hybrid functionals for the 50 screened candidates. Comparisons of HOMO energies calculated using B3LYP with those calculated using (a) PBE0, (b) TPSSh, and (c) M06. Corresponding comparisons of LUMO energies for (d) PBE0, (e) TPSSh, and (f) M06. The dashed lines represent perfect agreement between the two functionals. Evaluation metrics, including $R^2$, Spearman rank coefficient $\rho$, RMSE, and MAE are shown in each panel.

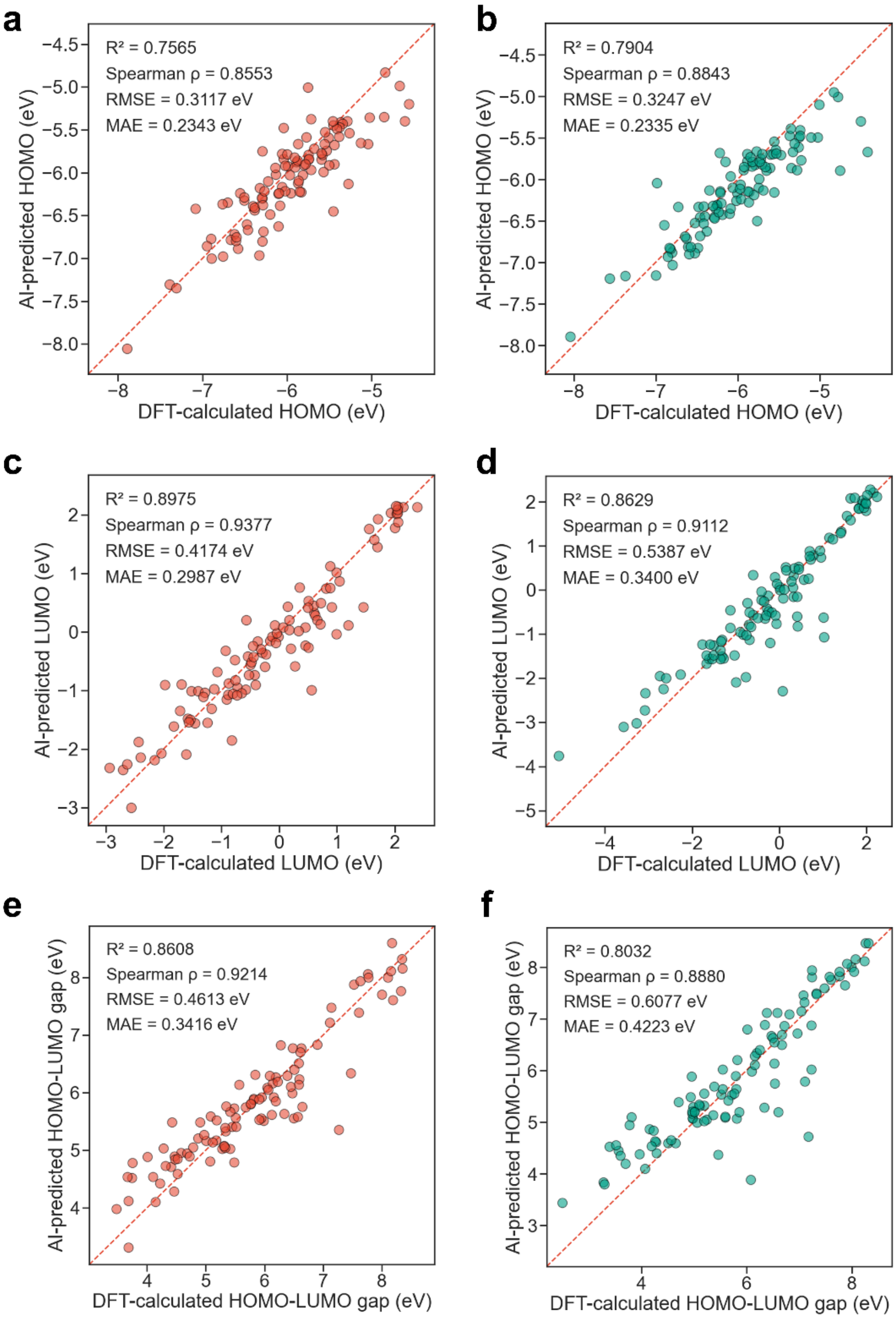


**Figure S37. Molecular-Size Dependence Analysis**

Comparisons between AI-predicted and B3LYP/6-31G(2df,p)-calculated values of (a) HOMO energy, (b) LUMO energy, and (c) HOMO–LUMO gap for 100 in-domain molecules containing up to nine heavy atoms. (d-f) Corresponding comparisons for 100 out-of-domain molecules containing 10–13 heavy atoms, comprising 26, 26, 24, and 24 molecules with 10, 11, 12, and 13 heavy atoms, respectively. The dashed lines represent perfect agreement between the two functionals. Evaluation metrics, including $R^2$, Spearman rank coefficient ρ, RMSE, and MAE are shown in each panel.

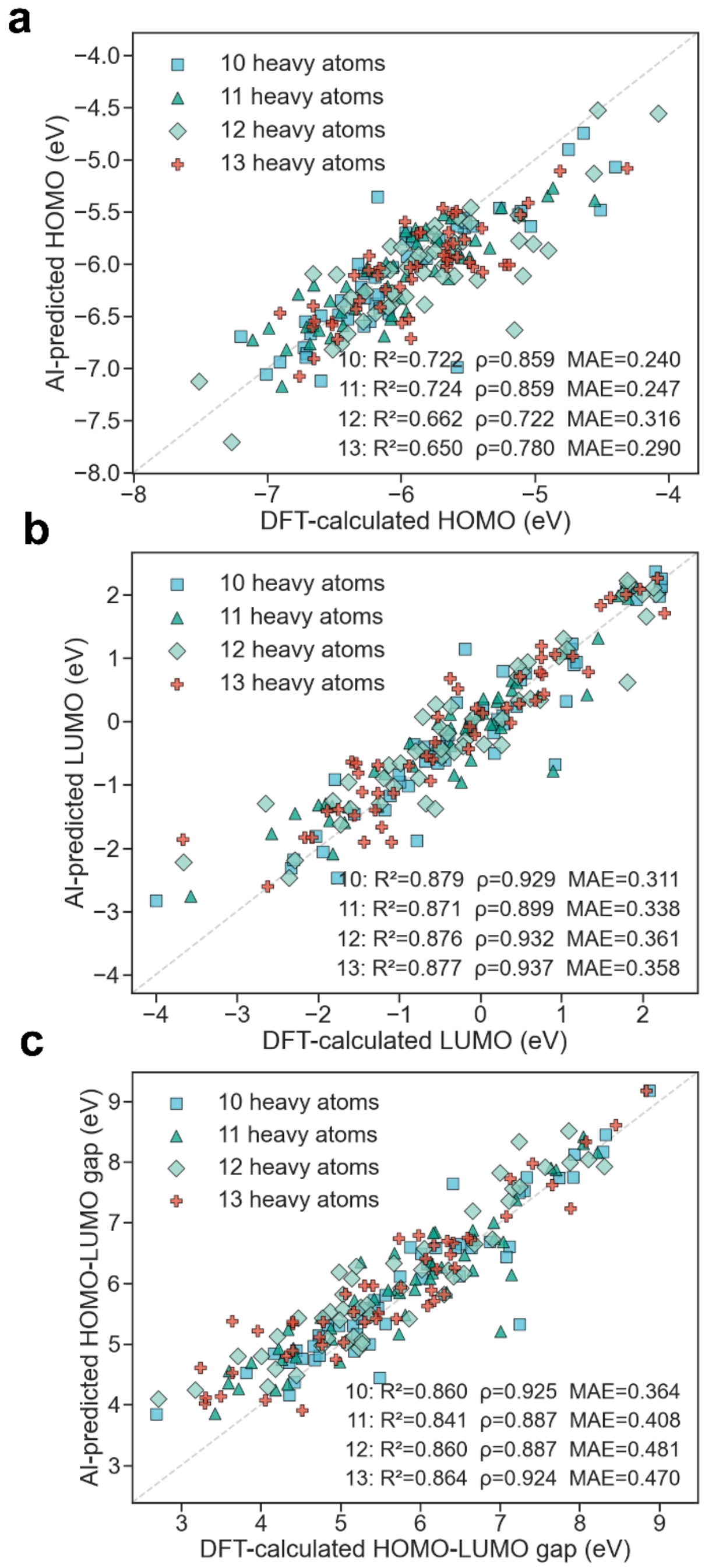


**Figure S38. Molecular-Size Dependence Analysis**

Comparisons between AI-predicted and B3LYP/6-31G(2df,p)-calculated values of (a) HOMO energy, (b) LUMO energy, and (c) HOMO–LUMO gap for 200 out-of-domain molecules containing 10–13 heavy atoms. Specifically, 50 molecules are selected at each heavy-atom count from 10 to 13, providing 200 additional validation molecules (10: square; 11: triangle; 12: diamond; 13: cross). The dashed lines represent perfect agreement between the two functionals. Evaluation metrics, including $R^2$, Spearman rank coefficient $\rho$, and MAE are shown in each panel.

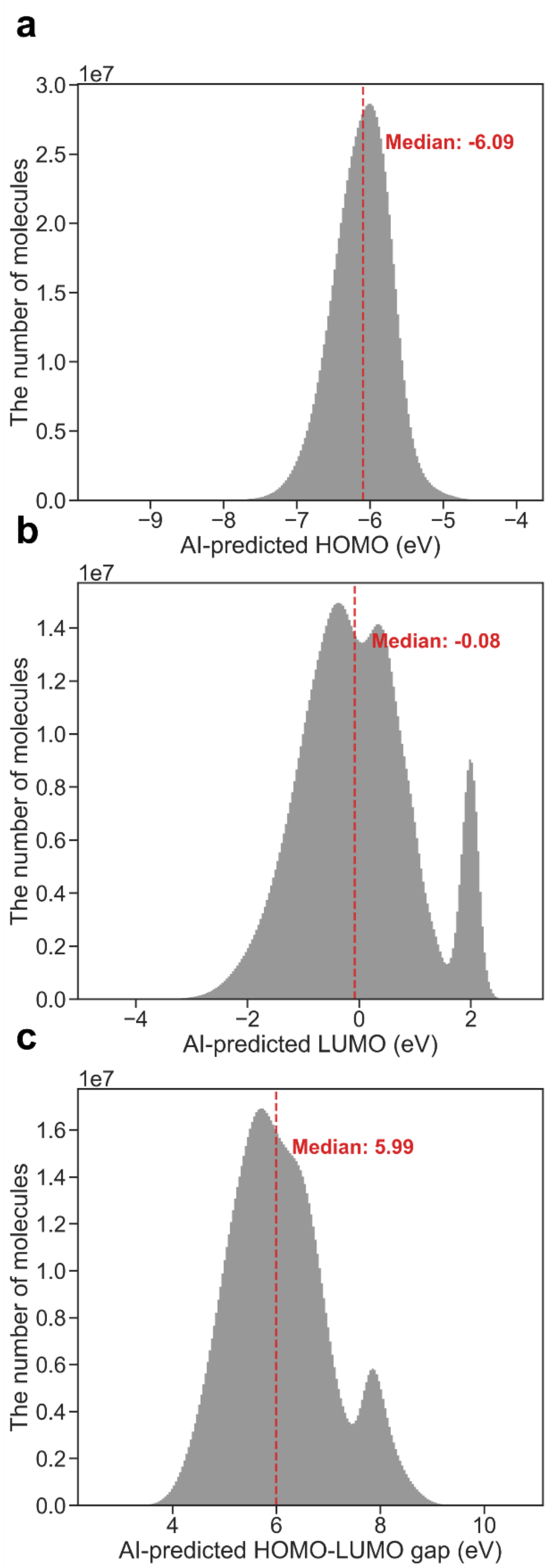


**Figure S39. AI-Based Prediction of Frontier Orbitals for GDB–13**

Distributions of AI-predicted HOMO, LUMO, and HOMO–LUMO gap values for organic molecules in the GDB–13 database. The top-performing DMPNN model, optimized using the QM9 dataset, is employed to predict these properties for organic molecules in the GDB–13 database.

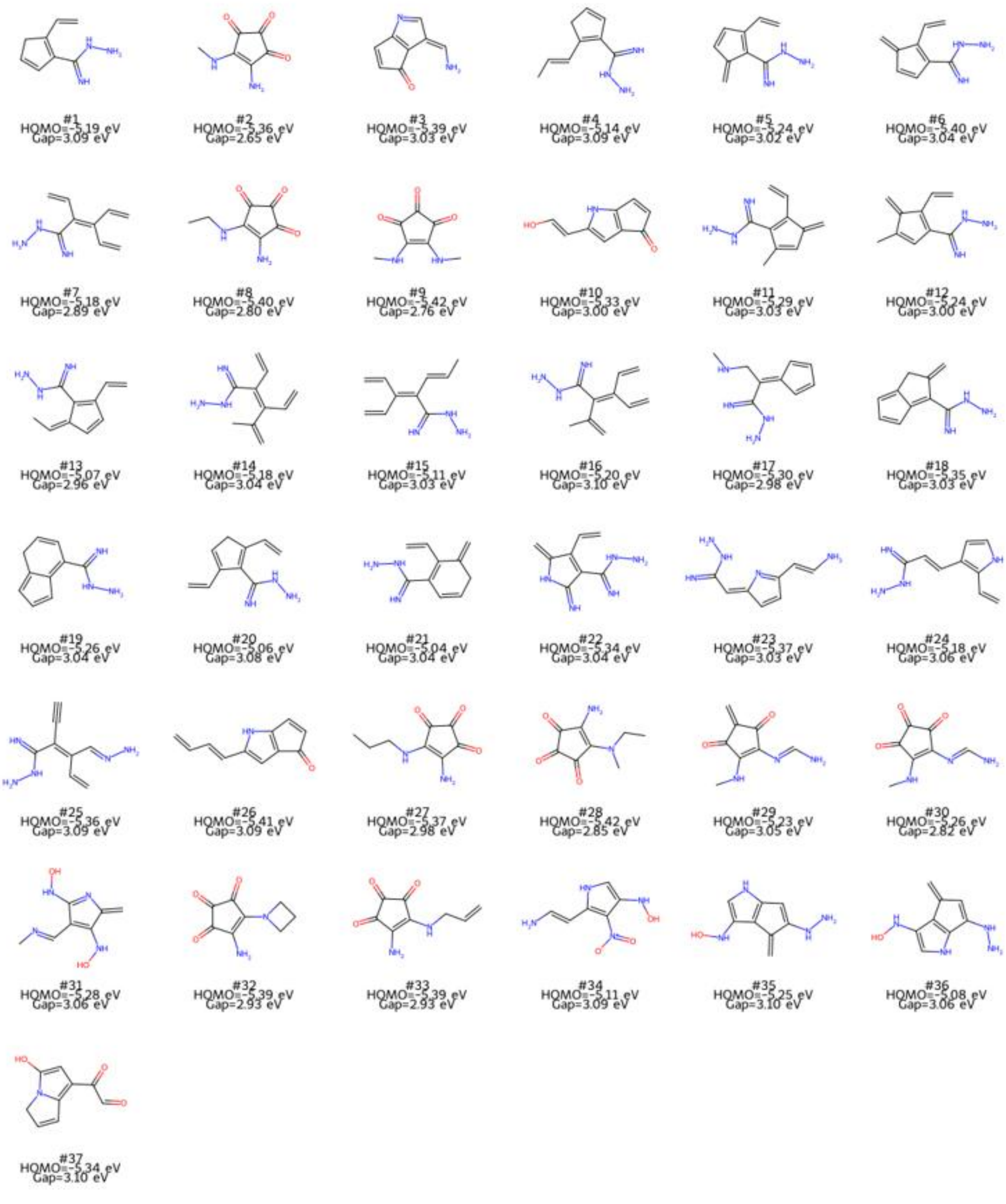


**Figure S40. 37 Donor Candidates Identified from GDB–13**

Chemical structures of 37 molecules identified through the DMPNN-based screening framework together with their AI-predicted HOMO energies and HOMO–LUMO gaps for GDB–13 database.

**Table S1. RDKit-Based Functional Group (Fragment) Descriptors**

Identity and definition of each fragment descriptor available in RDKit library (section 1).

| RDKit fragment descriptors | Definition |
|---|---|
| fr_Al_COO | Number of aliphatic carboxylic acids |
| fr_Al_OH | Number of aliphatic hydroxyl groups |
| fr_Al_OH_noTert | Number of aliphatic hydroxyl groups excluding tert-OH |
| fr_ArN | Number of N functional groups attached to aromatics |
| fr_Ar_COO | Number of Aromatic carboxylic acids |
| fr_Ar_N | Number of aromatic nitrogens |
| fr_Ar_NH | Number of aromatic amines |
| fr_Ar_OH | Number of aromatic hydroxyl groups |
| fr_COO | Number of carboxylic acids |
| fr_COO2 | Number of carboxylic acids |
| fr_C_O | Number of carbonyl O |
| fr_C_O_noCOO | Number of carbonyl O, excluding COOH |
| fr_C_S | Number of thiocarbonyl |
| fr_HOCCN | Number of C(OH)CCN-Ctert-alkyl or C(OH)CCNcyclic |
| fr_Imine | Number of Imines |
| fr_NH0 | Number of Tertiary amines |
| fr_NH1 | Number of Secondary amines |
| fr_NH2 | Number of Primary amines |
| fr_N_O | Number of hydroxylamine groups |
| fr_Ndealkylation1 | Number of XCCNR groups |
| fr_Ndealkylation2 | Number of tert-alicyclic amines (no heteroatoms, not quinine-like bridged N) |
| fr_Nhpyrrole | Number of H-pyrrole nitrogens |
| fr_SH | Number of thiol groups |
| fr_aldehyde | Number of aldehydes |
| fr_alkyl_carbamate | Number of alkyl carbamates (subject to hydrolysis) |
| fr_alkyl_halide | Number of alkyl halides |
| fr_allylic_oxid | Number of allylic oxidation sites excluding steroid dienone |
| fr_amide | Number of amides |
| fr_amidine | Number of amidine groups |
| fr_aniline | Number of anilines |

| | |
|---|---|
| fr_aryl_methyl | Number of aryl methyl sites for hydroxylation |
| fr_azide | Number of azide groups |
| fr_azo | Number of azo groups |
| fr_barbitur | Number of barbiturate groups |
| fr_benzene | Number of benzene rings |
| fr_benzodiazepine | Number of benzodiazepines with no additional fused rings |
| fr_bicyclic | Bicyclic |
| fr_diazo | Number of diazo groups |
| fr_dihydropyridine | Number of dihydropyridines |
| fr_epoxide | Number of epoxide rings |
| fr_ester | Number of esters |
| fr_ether | Number of ether oxygens (including phenoxy) |
| fr_furan | Number of furan rings |
| fr_guanido | Number of guanidine groups |
| fr_halogen | Number of halogens |

**Table S2. RDKit-Based Functional Group (Fragment) Descriptors**

Identity and SMARTS pattern of each fragment descriptor available in RDKit library (section 1).

| RDKit fragment descriptors | SMARTS patterns |
| --- | --- |
| fr_Al_COO | C-C(=O)[O;H1,-] |
| fr_Al_OH | [C&!$(C=O)]-[O&H1] |
| fr_Al_OH_noTert | [$(C-[O&X2&H1])&!$([C&X3](-[O&X2&H1])=[O&X1])&!$([C&D4]-[O&X2&H1])] |
| fr_ArN | [$(a-[N&X3&H2]),$(a-[N&H1][N&H2]),$(a-C(=[O&X1])[N&H1][N&H2]),$(a-C(=[N&H1])[N&H2])] |
| fr_Ar_COO | c-C(=O)[O;H1,-] |
| fr_Ar_N | n |
| fr_Ar_NH | [n&H1] |
| fr_Ar_OH | c[O&H1] |
| fr_COO | [#6]C(=O)[O;H1,-] |
| fr_COO2 | [C&X3](=O)[O&X1&H0&-,O&X2&H1] |
| fr_C_O | [C&X3]=[O&X1] |
| fr_C_O_noCOO | [C&!$(C-[O&H1])]=O |
| fr_C_S | C=[S&X1] |
| fr_HOCCN | [$([O&X2&H1][C&X4][C&X4&H2][N&X3&R1]),$([O&H1][C&X4][C&X4&H2][N&X3][C&X4](C)(C)C)] |
| fr_Imine | [N&v3](=C)-[#6] |
| fr_NH0 | [N&H0,n&H0] |
| fr_NH1 | [N&H1,n&H1] |
| fr_NH2 | [N&H2,n&H2] |
| fr_N_O | [N&!$(N=O)](-O)-C |
| fr_Ndealkylation1 | [$(N(-[C&H3])-C-[$(C~O),$(C-a),$(C-N),$(C=C)]),$(N(-[C&H2][C&H3])-C-[$(C~O),$(C-a),$(C-N),$(C=C)])] |
| fr_Ndealkylation2 | [$([N&R1]1(-C)CCC1),$([N&R1]1(-C)CCCC1),$([N&R1]1(-C)CCCCC1),$([N&R1]1(-C)CCCCCC1),$([N&R1]1(-C)CCCCCCC1)] |
| fr_Nhpyrrole | [n&H1] |
| fr_SH | [S&H1] |
| fr_aldehyde | [C&X3&H1](=O)[#6] |
| fr_alkyl_carbamate | C[N&H1]C(=O)OC |
| fr_alkyl_halide | [C&X4]-[Cl,Br,I,F] |

| | |
|---|---|
| fr_allylic_oxid | [$(C=C-C)&!$(C=C-C-[N,O,S])&!$(C=C-C-C-[N,O])&!$(C12=CC(=O)CCC1C1C(C3C(CCC3)CC1)CC2)] |
| fr_amide | C(=O)-N |
| fr_amidine | C(=N)(-N)-[!#7] |
| fr_aniline | c-[N&X3&!$(N=*)] |
| fr_aryl_methyl | [$(a-[C&H3]),$(a-[C&H2]-[C&H3]),$(a-[C&H2]-[C&H2]~[!N&!O]);!$(a(:a!:*):a!:*)] |
| fr_azide | [$(*-[N&X2&-]-[N&X2&+]#[N&X1]),$(*-[N&X2]=[N&X2&+]=[N&X1&-])] |
| fr_azo | [#6]-N=N-[#6] |
| fr_barbitur | C1C(=O)NC(=O)NC1=O |
| fr_benzene | c1ccccc1 |
| fr_benzodiazepine | [c&R2]12[c&R1][c&R1][c&R1][c&R1][c&R2]1[N&R1][C&R1][C&R1][N&R1]=[C&R1]2 |
| fr_bicyclic | [R2][R2] |
| fr_diazo | [N&+]#N |
| fr_dihydropyridine | [$([N&X3&H1]1-C=C-C-C=C1),$([N&v3]1=C-C-C=C-C1),$([N&v3]1=C-C=C-C-C1),$([N&X3&H1]1-C-C=C-C=C1)] |
| fr_epoxide | O1CC1 |
| fr_ester | [#6][C&X3](=O)[O&X2&H0][#6] |
| fr_ether | [O&D2]([#6])[#6] |
| fr_furan | o1cccc1 |
| fr_guanido | C(=N)(N)N |
| fr_halogen | [#9,#17,#35,#53] |

**Table S3. RDKit-Based Functional Group (Fragment) Descriptors**

Identity and definition of each fragment descriptor available in RDKit library (section 2).

| RDKit fragment descriptors | Definition |
|---|---|
| fr_hdrzine | Number of hydrazine groups |
| fr_hdrzone | Number of hydrazone groups |
| fr_imidazole | Number of imidazole rings |
| fr_imide | Number of imide groups |
| fr_isocyan | Number of isocyanates |
| fr_isothiocyan | Number of isothiocyanates |
| fr_ketone | Number of ketones |
| fr_ketone_Topliss | Number of ketones excluding diaryl, a,b-unsat. Dienones, heteroatom on Calpha |
| fr_lactam | Number of beta lactams |
| fr_lactone | Number of cyclic esters (lactones) |
| fr_methoxy | Number of methoxy groups -OCH3 |
| fr_morpholine | Number of morpholine rings |
| fr_nitrile | Number of nitriles |
| fr_nitro | Number of nitro groups |
| fr_nitro_arom | Number of nitro benzene ring substituents |
| fr_nitro_arom_nonortho | Number of non-ortho nitro benzene ring substituents |
| fr_nitroso | Number of nitroso groups, excluding NO2 |
| fr_oxazole | Number of oxazole rings |
| fr_oxime | Number of oxime groups |
| fr_para_hydroxylation | Number of para-hydroxylation sites |
| fr_phenol | Number of phenols |
| fr_phenol_noOrthoHbond | Number of phenolic OH excluding ortho intramolecular Hbond substituents |
| fr_phos_acid | Number of phosphoric acid groups |
| fr_phos_ester | Number of phosphoric ester groups |
| fr_piperdine | Number of piperdine rings |
| fr_piperzine | Number of piperzine rings |
| fr_priamide | Number of primary amides |
| fr_prisulfonamd | Number of primary sulfonamides |
| fr_pyridine | Number of pyridine rings |
| fr_quatN | Number of quaternary nitrogens |

| | |
|---|---|
| fr_sulfide | Number of thioether |
| fr_sulfonamd | Number of sulfonamides |
| fr_sulfone | Number of sulfone groups |
| fr_term_acetylene | Number of terminal acetylenes |
| fr_tetrazole | Number of tetrazole rings |
| fr_thiazole | Number of thiazole rings |
| fr_thiocyan | Number of thiocyanates |
| fr_thiophene | Number of thiophene rings |
| fr_unbrch_alkane | Number of unbranched alkanes of at least 4 members (excludes halogenated alkanes) |
| fr_urea | Number of urea groups |

**Table S4. RDKit-Based Functional Group (Fragment) Descriptors**

Identity and SMARTS pattern of each fragment descriptor available in RDKit library (section 2).

| RDKit fragment descriptors | SMARTS patterns |
|---|---|
| fr_hdrzine | [N&X3]-[N&X3] |
| fr_hdrzone | C=N-[N&X3] |
| fr_imidazole | n1cncc1 |
| fr_imide | N(-C=O)-C=O |
| fr_isocyan | N=C=O |
| fr_isothiocyan | N=C=S |
| fr_ketone | [#6][C&X3](=O)[#6] |
| fr_ketone_Topliss | [$([C&X3](=[O&X1])(C)[c,C])&!$([C&X3](=[O&X1])([C&H1]=C)[c,C])] |
| fr_lactam | N1C(=O)CC1 |
| fr_lactone | [C&R1](=O)[O&R1][C&R1] |
| fr_methoxy | [O&X2](-[#6])-[C&H3] |
| fr_morpholine | O1CCNCC1 |
| fr_nitrile | [N&X1]#[C&X2] |
| fr_nitro | [$([N&X3](=O)=O),$([N&X3&+](=O)[O&-])][!#8] |
| fr_nitro_arom | [$(c1(-[$([N&X3](=O)=O),$([N&X3&+](=O)[O&-])])ccccc1)] |
| fr_nitro_arom_nonortho | [$(c1(-[$([N&X3](=O)=O),$([N&X3&+](=O)[O&-])])ccccc1)&!$(cc-&!:*)] |
| fr_nitroso | [N&!$(N-O)]=O |
| fr_oxazole | c1ocnc1 |
| fr_oxime | [C&X3]=[N&X2]-[O&X2] |
| fr_para_hydroxylation | [$([c&H1]1[c&H1]cc(c[c&H1]1)~[$([#8,$([#8]~[H1,c,C])])]),$([c&H1]1[c&H1]cc(c[c&H1]1)~[$([#7&X3,$([#7](~[H1,c,C])~[H1,c,C])])]),$([c&H1]1[c&H1]cc(c[c&H1]1)-&!:[$([N&X3&H1,$(NC(=O)[H1,c,C])])])] |
| fr_phenol | [O&X2&H1]-c1ccccc1 |
| fr_phenol_noOrthoHbond | [$(c1(-[O&X2&H1])ccccc1)&!$(cc-&!:[C&H2]-[O&X2&H1])&!$(cc-&!:C(=O)[O;H1,-])&!$(cc-&!:C(=O)-[N&H2])] |
| fr_phos_acid | [$(P(=[O&X1])([$([O&X2&H1]),$([O&X1&-]),$([O&X2]P)])([$([O&X2&H1]),$([O&X1&-]),$([O&X2]P)])[$([O& |

| | |
|---|---|
| | X2&H1]),$([O&X1&-]),$([O&X2]P)]),$([P&+]([O&X1&-])([$([O&X2&H1]),$([O&X1&-]),$([O&X2]P)])([$([O&X2&H1]),$([O&X1&-]),$([O&X2]P)])[$([O&X2&H1]),$([O&X1&-]),$([O&X2]P)])] |
| fr_phos_ester | [$(P(=[O&X1])([O&X2][#6])([$([O&X2&H1]),$([O&X1&-]),$([O&X2][#6])])[$([O&X2&H1]),$([O&X1&-]),$([O&X2][#6]),$([O&X2]P)]),$([P&+]([O&X1&-])([O&X2][#6])([$([O&X2&H1]),$([O&X1&-]),$([O&X2][#6])])[$([O&X2&H1]),$([O&X1&-]),$([O&X2][#6]),$([O&X2]P)])] |
| fr_piperdine | N1CCCCC1 |
| fr_piperzine | N1CCNCC1 |
| fr_priamide | C(=O)-[N&H2] |
| fr_prisulfonamd | [N&H2]-S(=,-[O&X1&+0&-])(=,-[O&X1&+0&-])-[#6] |
| fr_pyridine | n1ccccc1 |
| fr_quatN | [$([N&X4&+]),$([N&X4]=*)] |
| fr_sulfide | [S&X2](-[#6])-C |
| fr_sulfonamd | N-S(=,-[O&X1;+0,-])(=,-[O&X1;+0,-])-[#6] |
| fr_sulfone | S(=,-[O&X1;+0,-])(=,-[O&X1;+0,-])(-[#6])-[#6] |
| fr_term_acetylene | C#[C&H1] |
| fr_tetrazole | c1nnnn1 |
| fr_thiazole | c1scnc1 |
| fr_thiocyan | S-C#N |
| fr_thiophene | s1cccc1 |
| fr_unbrch_alkane | [R0&D2][R0&D2][R0&D2][R0&D2] |
| fr_urea | C(=O)(-N)-N |

**Table S5. Fragment Composition of Morgan Fingerprint Bits**

A selected set of bit IDs in the Morgan fingerprint representation, along with the corresponding fragment SMILES, atom invariant/centered environment, occurrence count, and fraction (%) within each bit. **Atom-invariant label format:** *[atom symbol; radius=x; deg=y; H=z; arom=a; charge=q]*. Here, *atom symbol* is a central atom type, *radius* is the Morgan fingerprint radius (the maximum bond distance from a central atom), *deg* is the number of neighboring atoms directly bonded from a central atom, *H* is the number of hydrogen atoms attached to a central atom, *arom* indicates aromaticity (1 = yes, 0 = no), and *charge* is the formal atomic charge.

| Bit ID | Framework SMILES | Atom invariant / centered environment | Count | Fraction within bit (%) |
|---|---|---|---|---|
| 314 | O=C | [O; radius=1; deg=1; H=0; ring=0] | 19,502 | 86.8% |
| 314 | O=c | [O; radius=1; deg=1; H=0; ring=0] | 2,937 | 13.1% |
| 314 | c([NH-])([nH+]c)oc | [C; radius=2; deg=3; H=0; ring=1; arom=1] | 35 | 0.2% |
| 650 | O | [O; radius=0; deg=1; H=0; ring=0] | 49,510 | 99.6% |
| 650 | C(C)c(c)c | [C; radius=2; deg=2; H=2; ring=0] | 158 | 0.3% |
| 650 | C(=C)(N)[NH-] | [C; radius=1; deg=3; H=0; ring=1] | 48 | 0.1% |
| 674 | C | [C; radius=0; deg=2; H=0; ring=0] | 32,245 | 99.4% |
| 674 | C(#C)CC | [C; radius=2; deg=2; H=0; ring=0] | 133 | 0.4% |
| 674 | O(C)c(c)o | [O; radius=2; deg=2; H=0; ring=0] | 62 | 0.2% |
| 694 | C | [C; radius=0; deg=2; H=1; ring=0] | 18,903 | 97.5% |
| 694 | C(O)C(C)C | [C; radius=2; deg=2; H=2; ring=0] | 457 | 2.4% |
| 694 | o(nc)c(n)=O | [O; radius=2; deg=2; H=0; ring=1; arom=1] | 22 | 0.1% |
| 790 | N#C | [N; radius=1; deg=1; H=0; ring=0] | 16,567 | 99.6% |
| 790 | N(C)Cc | [N; radius=2; deg=2; H=1; | 39 | 0.2% |

| | | | | |
|---|---|---|---|---|
| | | ring=0] | | |
| 790 | C(C)(=O)C(C)=O | [C; radius=2; deg=3; H=0; ring=0] | 26 | 0.2% |
| 1114 | N | [N; radius=0; deg=2; H=1; ring=1] | 18,599 | 71.2% |
| 1114 | [9] | [N; radius=0; deg=2; H=1; ring=1; arom=1] | 7,465 | 28.6% |
| 1114 | C(#N)C(C)C | [C; radius=2; deg=2; H=0; ring=0] | 44 | 0.2% |
| 1380 | C | [C; radius=0; deg=3; H=0; ring=1] | 26,139 | 52.5% |
| 1380 | c | [C; radius=0; deg=3; H=0; ring=1; arom=1] | 21,453 | 43.1% |
| 1380 | C(C)(C)O | [C; radius=1; deg=3; H=1; ring=1] | 2,163 | 4.3% |
| 1690 | [NH+] | [N; radius=0; deg=3; H=1; ring=1; charge=+1] | 14,364 | 99.5% |
| 1690 | C(C)(C)(C)N | [C; radius=1; deg=4; H=0; ring=1] | 50 | 0.3% |
| 1690 | C(CO)Nc | [C; radius=2; deg=2; H=2; ring=0] | 29 | 0.2% |
| 1873 | c | [C; radius=0; deg=2; H=1; ring=1; arom=1] | 18,043 | 50.4% |
| 1873 | C | [C; radius=0; deg=2; H=1; ring=1] | 17,629 | 49.2% |
| 1873 | O(C)C | [O; radius=1; deg=2; H=0; ring=0] | 143 | 0.4% |

## REFERENCES


[1] RDKit: Open-source cheminformatics. https://www.rdkit.org.

[2] G. Ke, Q. Meng, T. Finley, T. Wang, W. Chen, W. Ma, Q. Ye, T. Y. Liu, *Adv. Neural Inf. Process. Syst.* **2017**, *30*.

[3] T. Chen, C. Guestrin, in *Proceedings of the 22nd acm sigkdd international conference on knowledge discovery and data mining*, **2016**, p. 785.

[4] L. Prokhorenkova, G. Gusev, A. Vorobev, A. V. Dorogush, A. Gulin, *Adv. Neural Inf. Process. Syst.* **2018**, *31*.

[5] E. Heid, K. P. Greenman, Y. Chung, S. C. Li, D. E. Graff, F. E. Vermeire, H. Wu, W. H. Green, C. J. McGill, *J. Chem. Inf. Model.* **2024**, *64*, 9.

[6] K. Yang, K. Swanson, W. Jin, C. Coley, P. Eiden, H. Gao, A. Guzman-Perez, T. Hopper, B. Kelley, M. Mathea, A. Palmer, *J. Chem. Inf. Model.* **2019**, *59*, 3370.

[7] S. Chithrananda, G. Grand, B. Ramsundar, *arXiv preprint arXiv:2010.09885* **2020**.

[8] Y. Liu, M. Ott, N. Goyal, J. Du, M. Joshi, D. Chen, O. Levy, M. Lewis, L. Zettlemoyer, V. Stoyanov, *arXiv preprint arXiv:1907.11692* **2019**.

[9] J. Gilmer, S. S. Schoenholz, P. F. Riley, O. Vinyals, G. E. Dahl, in *International conference on machine learning*, Pmlr, **2017**, p. 1263.
